# Naturally occurring variations in sequence length creates microRNA isoforms that differ in argonaute effector complex specificity

**DOI:** 10.1186/1758-907X-1-12

**Published:** 2010-06-09

**Authors:** H Alexander Ebhardt, Amber Fedynak, Richard P Fahlman

**Affiliations:** 1 Department of Biochemistry, School of Molecular and Systems Medicine, University of Alberta, Edmonton, T6G 2H7, Canada

## Abstract

**Background:**

Micro(mi)RNAs are short RNA sequences, ranging from 16 to 35 nucleotides (miRBase; http://www.mirbase.org). The majority of the identified sequences are 21 or 22 nucleotides in length. Despite the range of sequence lengths for different miRNAs, individual miRNAs were thought to have a specific sequence of a particular length. A recent report describing a longer variant of a previously identified miRNA in *Arabidopsis thaliana *prompted this investigation for variations in the length of other miRNAs.

**Results:**

In this paper, we demonstrate that a fifth of annotated *A. thaliana *miRNAs recorded in miRBase V.14 have stable miRNA isoforms that are one or two nucleotides longer than their respective recorded miRNA. Further, we demonstrate that miRNA isoforms are co-expressed and often show differential argonaute complex association. We postulate that these extensions are caused by differential cleavage of the parent precursor miRNA.

**Conclusions:**

Our systematic analysis of *A. thaliana *miRNAs reveals that miRNA length isoforms are relatively common. This finding not only has implications for miRBase and miRNA annotation, but also extends to miRNA validation experiments and miRNA localization studies. Further, we predict that miRNA isoforms are present in other plant species also.

## Background

Micro(mi)RNAs are important for gene regulation [1] and for cell fate decisions during development [2]. Aberrant levels of miRNAs are seen in various disease states [3-6]. miRNAs are transcribed from one strand of their genomic loci into a primary miRNA transcript, which folds into a characteristic bulge with stem-loop conformation [7]. In plants, the primary transcript is cleaved by a Dicer-like (DCL) RNase III enzyme, DCL1, into an approximately 19 bp duplex with a two-nucleotide (nt) overhang at either end [8]. Of the two strands forming the duplex, one strand, designated miRNA*, is typically degraded while the other is incorporated into the argonaute (AGO)-containing effector complex [9,10]. Co-immunoprecipitation experiments demonstrate an enrichment of miRNAs in AGO1, whereas AGO2 shows depletion of miRNAs compared with non-immunoprecipitated samples [11].

The biological significance of sequence length heterogeneity has been recently identified for a mature miRNA in *Arabidopsis thaliana*, in which ath-MIR168 is processed as miRNAs of 21 and 22 nucleotides in length from its two genomic loci. Vaucheret demonstrated that reducing the amount of 21 nt miRNA greatly reduces homeostasis and leads to developmental defects of the plant, especially in environmentally challenging conditions [12]. In general, it is appreciated that there is variation in the lengths of different miRNAs, as the mature miRNAs listed in miRBase http://www.mirbase.org/ are between 16 and 35 nucleotides in length [13]. In miRBase V.14 there are 209 small RNA sequences identified for in *A. thaliana*, of which 7%, 79%, 11% and 3% are 20, 21, 22 and 24 nt in length, respectively. The reason and function for this heterogeneity is unclear and we are unaware of any systematic investigation into non-uniform length distributions of individual miRNAs. Each annotated miRNA in miRBase is a single defined sequence, and there are no details on the possibility of variable sequence length. Sequence length variation may have been overlooked previously, as small variations in the sequence length might not have been thought to alter the function of individual miRNAs, as they are directed to their targets by base pairing.

Recent reports show however, that alterations in miRNA length can potentially lead to dramatic effects on miRNA function in organisms such as *A. thaliana*, in which the identity of the first 5' nucleotide of the miRNA is the major determinant for AGO protein association [11,14]. Sequence-specific AGO association has been characterized for most *A. thaliana *AGO complexes [11,14,15]. Of these, AGO1 is the major AGO in the pathway of miRNA post-transcriptional gene silencing [16-19], whereas AGO4 functions in repeat-associated silencing of RNA accumulation and in regulating loci- specific DNA methylation [20,21].

To investigate the frequency with which additional nucleotides on the 5' ends of miRNAs are observed, we queried several published *A. thaliana *small RNA datasets collected by pyrophosphate and Solexa/Iillumina http://www.illumina.com sequencing techniques. The approach of analyzing small RNA sequencing datasets has previously proven successful for the identification of post-transcriptional modifications in small RNAs [22-25]. Using similar methods, we queried all of the annotated miRNAs from *A. thaliana *(miRBase V.14) for 5' extensions of one to three nucleotides based on nucleotides present in the pre-miRNA hairpin.

The datasets investigated were from three small RNA sequencing studies including a small RNA transcriptome that responds to changing phosphate levels [26], an RNA analysis of the dicer (DCL2/DCL3/DCL4) triple mutant [27], and a study on RNAs that are co-immunoprecipitated with different AGO proteins [11]. In total, these datasets contained 51,907,309 redundant small RNA sequences.

## Results

### MiRNAs with SNE

**Figure 1:**
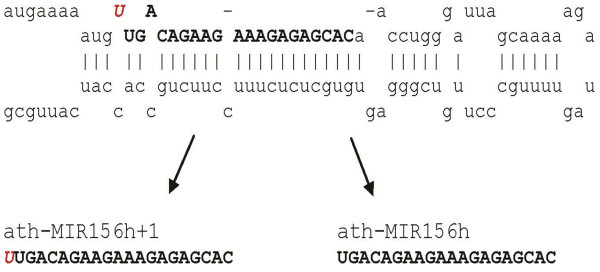
**Two mature micro(mi)RNA sequences derived from the ath-MIR156h pre-miRNA hairpin**. The primary hairpin ath-MIR156h gives rise to two mature isoforms of ath-MIR156h, which differ by one nucleotide on the 5' terminus.

For our *in silico *northern blot analysis, we queried each dataset mentioned above with each miRNA sequence for *A. thaliana *listed in miRBase V.14. In addition to the recorded mature miRNA sequence, we extended each mature miRNA at the 5' by 1, 2 and three nucleotide(s) according to the hairpin sequence of the miRNA. To our surprise, numerous miRNAs encompass a 5' single nucleotide extension (SNE) compared with the recorded mature miRNA; for example, ath-MIR156h. The SNE of ath-MIR156h is an additional 5' uridine/uracil (U) that is not reported in the annotated mature miRNA sequence [13,28,29], but is present in the parental pre-miRNA hairpin (Figure 1); the extended form of ath-MIR156h is henceforth referred to as ath-MIR156h+1. Both ath-MIR156h and ath-MIR156h+1 were present in small RNA samples, independent of genetic background, environmental effects, tissue types and sequencing technologies (see Table 1). A consistent cloning ratio of 7:3 (ath-MIR156h+1:ath-MIR156h) was observed, despite large variations in total abundance in different genetic backgrounds and tissues. One exception to the 7:3 ratio was found in small RNA cloning data originating from the plant root, in which the two miRNAs were found in a 1:1 ratio (Table 1). The high frequency of occurrence of the ath-MIR156h+1 sequence and the reproducibility between datasets suggests a biological role for this long variant of ath-MIR156h.

**Table 1:** ath-MIR156h exists in two isoforms across seven datasets

	ath-MIR156h+1, %, (*n*)	ath-MIR156h, %, (*n*)	Reference
**Col-0**	71.7 (2,445)	28.2 (962)	[27]
**SUr2a**	69.9 (25,789)	30.0 (11,102)	[27]
**S234a**	70.2 (24,472)	29.7 (10,802)	[27]
**Shoot +Pi**	75.9 (328)	24.1 (104)	[26]
**Shoot -Pi**	74.1 (413)	25.9 (144)	[26]
**Root -Pi**	54.5 (54)	45.5 (45)	[26]
**Root +Pi**	50.4 (70)	49.6 (71)	[26]

Ath-MIR156h+1 has a single extra nucleotide on the 5' terminus compared with ath-MIR156h. Both isoforms co-exist across various genetic backgrounds and under various environmental conditions. WT (Col-0), ΔRDR2 (Sur2a) and DCL2/DCL3/DCL4-triple (S234a) mutant *Arabidopsis thaliana *[27], root and shoot small RNA transcriptomes under varying phosphate levels GEO: GSE17741 [26].

**Figure 2:**
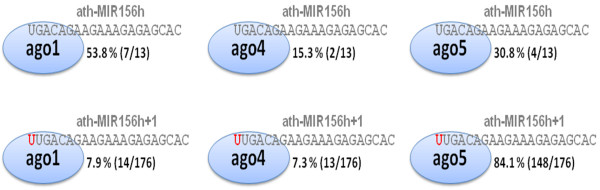
**AGO association of ath-MIR156h isoforms**. Mature miRNA ath-MIR156h resides mostly in the argonaute (AGO)1 effector complex, whereas the majority of ath-MIR156h+1 resides in the AGO5 effector complex. The cloning ratios are given in percentage and absolute numbers. Statistical analysis on the AGO association frequencies using the Freeman-Halton extension of the Fisher exact probability test [33] revealed a highly significant result (*P *= 2.5 × 10^-5^) for the observed difference in AGO association. Data originated from the AGO association datasets [11].

The distribution of ath-MIR156h+1 in various AGO complexes differs from that of the parental ath-MIR156h miRNA. AGO association was analyzed by determining the frequencies with which ath-MIR156h and ath-MIR156h+1 were identified in previously published datasets of miRNAs co-purified with AGO1, AGO2, AGO4 and AGO5 [11]. The current model for *A. thaliana *miRNAs predicts that ath-MIR156h should be mostly present in AGO1-RISC complexes, as the miRNA possesses a 5' U nucleotide. As predicted, over half of ath-MIR156h miRNAs reside in AGO1 complexes (54%), whereas the remainder are split into AGO5 (31%) and AGO4 (15%) effector complexes. No association with AGO2 was found. However, in addition to a 10-fold increased frequency of detection, ath-MIR156h+1 was detected almost exclusively in AGO5 complexes (84.1%), with few sequences detected in AGO1 (8%) and AGO4 (7%) datasets (Figure 2). This shift in association with AGOs was not initially predicted, as ath-MIR156h+1 still has a 5' U, nonetheless a shift in the frequency of AGO association was observed.

### MiRNAs with a double nucleotide extension

In our first example, we demonstrated that both ath-MIR156h and ath-MIR156h+1 coexist within the plant at constant ratios, with each miRNA isoforms showing preferential AGO association. A second class of miRNAs identified possess two additional 5' nucleotides. An example of this class is ath-MIR775, which exists as both ath-MIR775 and ath-MIR775+2; the latter has two additional 5' U nucleotides, with both of these nucleotides present in the pre-miRNA hairpin. The parental miRNA and ath-MIR775+2 were found at comparable frequencies in all the datasets (1858 and 1587 occurrences, respectively, in the AGO association database). Conversely, there was a negligible occurrence of the +1 miRNA. There are two possible explanations for this exclusive occurrence of ath-MIR775 and ath-MIR775+2. Cleavage events generating the mature miRNAs might generate the two variable length miRNAs forms (0 and +2) exclusively. Alternatively, all three lengths (0, +1 and +2) might be generated, but with only the 0 and +2 forms being stabilized and protected from degradation.

Analysis of the AGO associations of ath-MIR775 and ath-MIR755+2 revealed a difference in the identity of preferential AGO association. In more than 95% of results, the ath-MIR775 sequence was found to be associated with AGO1, whereas the ath-MIR775+2 variant was associated with AGO5 in nearly 70% of cases (Table 2).

**Table 2:** Cloning frequencies of ath-MIR775 sequences in the argonaute (AGO) association datasets

	AGO1	AGO2	AGO4	AGO5
**ath-MIR775**	1788	4	22	44
**ath-MIR775+1**	38	0	7	81
**ath-MIR775+2**	409	0	86	1092

### Not all miRNAs are heterogeneously processed

Heterogeneity in mature miRNA lengths is not the rule, as many do not exhibit detectable amounts of variable length processing. Examples include ath-MIR168b which was observed 86,634 times in the four different AGO association datasets, whereas the ath-MIR168b+1 sequence was observed only 34 times. This observed frequency is within the 3% insertion/deletion error rate of pyrophosphate sequencing [30]. To date, there have been no detailed analyses by Illumina or Solexa sequencing of the frequency of insertion and deletion errors. The presence of both types of miRNAs (variable and homogeneous lengths) suggests that the variable lengths of some miRNAs are not simply the result of 'ragged end' processing of all miRNAs, but are a specific process for a subset of miRNAs.

### Overall frequency of variable length miRNAs

**Figure 3:**
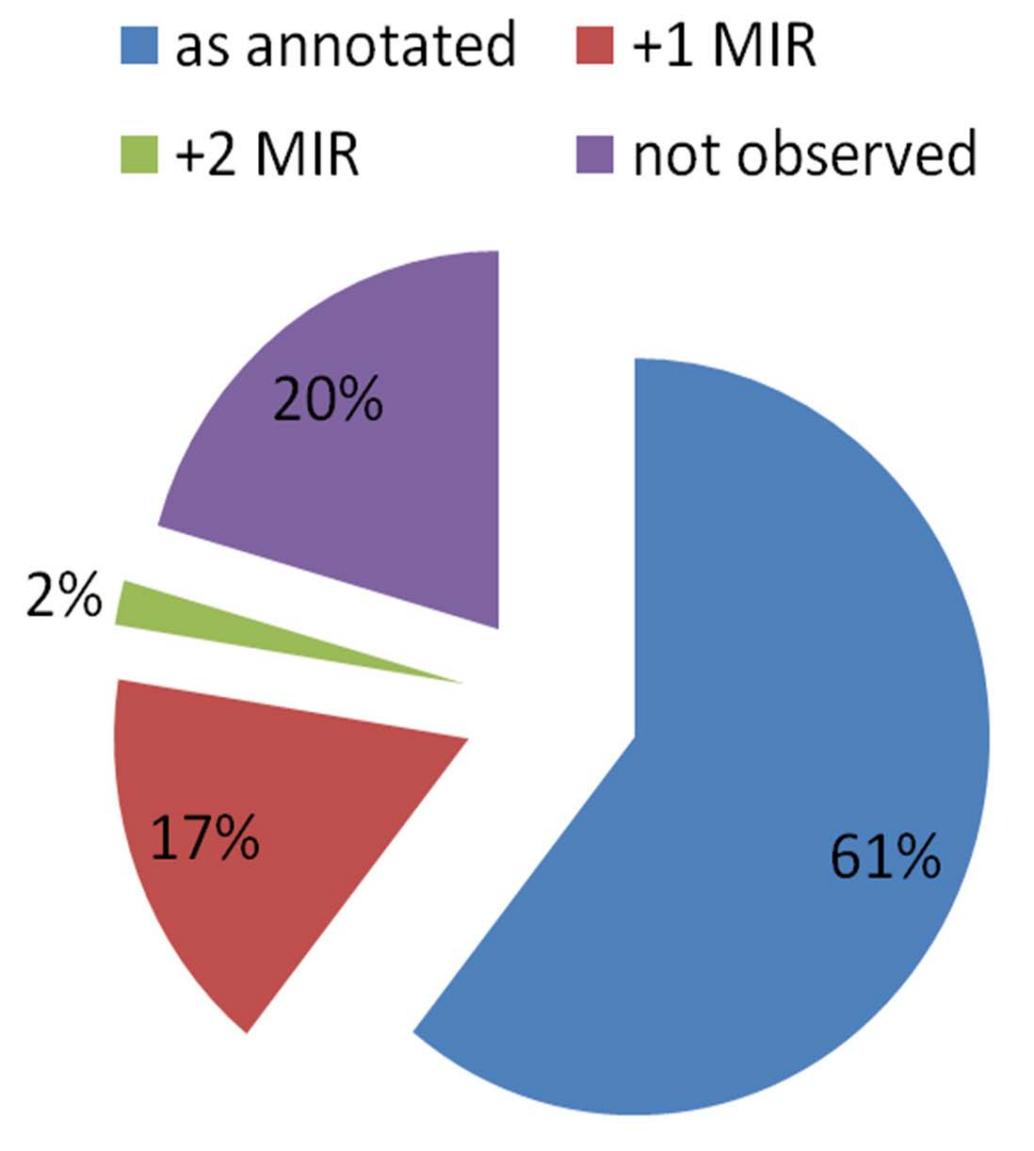
**Frequency of extra 5' nucleotides**. Several small *Arabidopsis thaliana *RNA datasets were analyzed for the occurrence of miRNAs with extra 5' nucleotides. From these datasets, 61% of the *A. thaliana *miRNAs listed in miRBase had no significant occurrence of extra nucleotides, 20% of the annotated miRNAs were not observed in the datasets at all, and 19% of miRNA had a significant proportion of either one (+1 MIR) or two (+2 MIR) nucleotides.

In addition to the two examples outlined above, we systematically queried the entire ath-MIR dataset from miRBase V.14 in an *in silico *northern blot analysis. The presence and frequency of each miRNA sequence, including the +1, +2 and +3 extended miRNA forms, was queried against the database (Figure 3; see Additional File 1). The sequence had to be present in a dataset at least six times to be counted. Of the 209 annotated miRNAs in miRBase, 166 were found in this analysis. Of the observed miRNA sequences, 35 were found to have a single nucleotide addition, and four were observed had two nucleotides added. In total, nearly 20% of the annotated miRNAs had additional 5' nucleotides. These 5' extensions were not simply misannotated miRNAs, as isoforms of various lengths co-existed. In addition to identifying the miRNAs, we examined miRNAs exhibiting length isoforms for changes in AGO association (see Additional File 2).

In addition to our presented *in silico *data, previous work using a genetic approach also suggests co-existence of miRNA and miRNA+1 and the importance of their co-expression. A recent report described and confirmed the occurrence of a long (22 nt) form of ath-miR168 [12]. In addition, experiments by Vaucheret and data from other studies also reveals evidence of long miRNA variants; for example, careful examination of previously published miRNA northern blots found the presence of double bands for some miRNAs, such as for ath-miR169, ath-miR156 and ath-miR172 [31].

## Conclusions

We have presented evidence arising from several small RNA sequencing experiments that supports the co-existence of mature miRNAs and their 5' extended forms in *A. thaliana*. Our results expand the previous genetic evidence of variability in miRNA sequence length [12] by revealing that nearly a fifth of miRNAs identified in *A. thaliana *have additional nucleotide(s) on their 5' ends. These 5' extended miRNAs are not simply misannotated, as both longer and shorter forms of the miRNAs co-exist. Additionally, we provide evidence that the 5' end variations can result in changes in the type of AGOs with which these miRNA isoforms preferentially associate. Differences in AGO associations suggest alterations in the biological functioning of the different observed forms of these miRNAs. These variable length miRNAs could essentially be considered miRNA isoforms and should be included in any annotation of miRNAs.

## Methods

A Perl script mapped each mature miRNA to their respective hairpin, recorded the hairpin sequence, then appended one, two or three nucleotide(s) to the 5' of the mature miRNA. For miRNA, the Perl script recorded five sequences: hairpin, mature miRNA, +1 miRNA, +2 miRNA and +3 miRNA. All * sequences were ignored and not used for analysis. Scripts are available online under GPLV.2 http://www.bioinformatics.org/ebbie. The output file in FASTA format was used for an *in silico *northern blot, which probed all computer-generated small RNA sequences in various datasets (GEO:GSE17741 [26], GEO:GSE5343 [32] and ath-sbs [27]) using a modified *Ebbie-(mis)match-AGO v1 *[22] script. To determine AGO complex affiliation, the computer-generated small RNAs were similarly compared against the AGO1, AGO2, AGO4 and AGO5 small RNA datasets [11] using *Ebbie-(mis)match-AGO v2*. Computation was performed on an IBM system (Model x3850; IBM Computers, Markham, ON, Canada).

## Competing interests

The authors declare that they have no competing interests.

## Authors' contributions

HAE designed experiments and analyzed data. AF wrote the Perl scripts. HAE and RPF wrote the manuscript. All authors read and approved the final manuscript.

## Supplementary Material

Additional file 1 **Validation of computer-generated miRNA isoforms**. An *in silico *northern blot was performed of all computer-generated miRNA isoforms by scanning seven different datasets for the expression of each *in silico *miRNA isoform. Click here for file

Additional file 2 **Validated miRNA isoforms and their AGO association**. All validated miRNA isoforms from Additional file 1 were scanned across various argonaute complexes. Click here for file
