# Supplementary material for: Naturally occurring variations in sequence length creates microRNA isoforms that differ in argonaute effector complex specificity
Source: Silence. 2010 Jun 9;1:12. doi: 10.1186/1758-907X-1-12 (PMC2901367; doi:10.1186/1758-907X-1-12)
Supplement: Additional file 1 — Validation of computer-generated miRNA isoforms. An in silico northern blot was performed of all computer-generated miRNA isoforms by scanning seven different datasets for the expression of each in silico miRNA isoform. [file 1758-907X-1-12-S1.PDF]

Additional File 1

Naturally Occurring Variations in Sequence Length Creates MiRNA Isoforms that Differ in Argonaute Effector Complex Specificity.

H. Alexander Ebhardt, Amber Fedynak, Richard P. Fahlman

In Silico Northern analysis to determine the frequency that all A. thaliana miRNAs and their +1, +2 and +3 isoforms are observed in 12 small RNA sequencing datasets.

For each miRNA the pre-miRNA hairpin as well as the mature miRNA and length isoforms. Values list the the number of occurances each sequence exists in a given dataset.

mature-5var.fa	Col0_raw.red.fasta	S234a_raw.red.fasta	SUr2a_raw.red.fasta	GSM442932-23482.red	GSM442933-24768.red	GSM442934-24766.red	GSM442935-23491.red	GSM121453-rdr2.red.fasta	GSM121454_rdr6.red.fasta	GSM121455_Col-0.red.fasta	GSM121456-dcl1-7.red.fasta	GSM121457_dcl234.red.fasta
>ath-MIR156a MI0000178 Arabidopsis thaliana miR156a stem-loop												
CAAGAGAAACGCAAAGAAACTGACAGAAGAGAGTGAGCACACAAAGGCAATTTGCATATCATTGCACTTGCTTCTCTTGCGTGCTCACTGCTCTTTCTGTCAGATTCCGGTGCTGATCTCTTT	0	0	0	0	0	0	0	0	0	0	0	0
>ath-miR156a MI0000178 Arabidopsis thaliana miR156a stem-loop												
TGACAGAAGAGAGTGAGCAC	27527	68907	69065	63209	21950	74179	58692	7	12	1	0	1
>+1 ath-miR156a												
CTGACAGAAGAGAGTGAGCAC	24	64	73	48	26	57	61	0	0	0	0	0
>+2 ath-miR156a												
ACTGACAGAAGAGAGTGAGCAC	12	6	2	70	32	44	28	0	0	0	0	0
>+3 ath-miR156a												
AACTGACAGAAGAGAGTGAGCAC	16	15	11	31	2	2	2	0	0	0	0	0
>ath-MIR156b MI0000179 Arabidopsis thaliana miR156b stem-loop												
GCTAGAAGAGGGAGAGATGGTGATTGAGGAATGCAACAGAGAAAACTGACAGAAGAGAGTGAGCACATGCAGGCACTGTTATGTGTCTATAACTTTGCGTGTGCGTGCTCACCTCTCTTTCTGTCAGTTGCCTATCTCTGCCTGCTTGACCTCTCTCTCTCTCTCTCTCTCTCAAATTTGGCT	0	0	0	0	0	0	0	0	0	0	0	0
>ath-miR156b MI0000179 Arabidopsis thaliana miR156b stem-loop												
TGACAGAAGAGAGTGAGCAC	27527	68907	69065	63209	21950	74179	58692	7	12	1	0	1
>+1 ath-miR156b												
CTGACAGAAGAGAGTGAGCAC	24	64	73	48	26	57	61	0	0	0	0	0
>+2 ath-miR156b												
ACTGACAGAAGAGAGTGAGCAC	12	6	2	70	32	44	28	0	0	0	0	0
>+3 ath-miR156b												
AACTGACAGAAGAGAGTGAGCAC	16	15	11	31	2	2	2	0	0	0	0	0
>ath-MIR156c MI0000180 Arabidopsis thaliana miR156c stem-loop												
CGCATAGAAACTGACAGAAGAGAGTGAGCACACAAAGGCACTTTGCATGTTCGATGCATTTGCTTCTCTTGCGTGCTCACTGCTCTATCTGTCAGATTCCGGCT	0	0	0	0	0	0	0	0	0	0	0	0
>ath-miR156c MI0000180 Arabidopsis thaliana miR156c stem-loop												
TGACAGAAGAGAGTGAGCAC	27527	68907	69065	63209	21950	74179	58692	7	12	1	0	1
>+1 ath-miR156c												
CTGACAGAAGAGAGTGAGCAC	24	64	73	48	26	57	61	0	0	0	0	0
>+2 ath-miR156c												
ACTGACAGAAGAGAGTGAGCAC	12	6	2	70	32	44	28	0	0	0	0	0
>+3 ath-miR156c												
AACTGACAGAAGAGAGTGAGCAC	16	15	11	31	2	2	2	0	0	0	0	0
>ath-MIR156d MI0000181 Arabidopsis thaliana miR156d stem-loop												
GATGGGGGAAAAGAAGTTGACAGAAGAGAGTGAGCACACAAAGGGGAAGTTGTATAAAAGTTTTGTATATGGTTGCTTTTGCGTGCTCACTCTCTTTTTGTCATAACTTCTCCTTCAT	0	0	0	0	0	0	0	0	0	0	0	0

>ath-miR156d MI0000181<br>Arabidopsis thaliana miR156d<br>stem-loop												
TGACAGAAGAGAGTGAGCAC	27527	68907	69065	63209	21950	74179	58692	7	12	1	0	1
>+1 ath-miR156d												
TTGACAGAAGAGAGTGAGCAC	6903	27882	27701	287	396	44179	35628	3	1	0	1	0
>+2 ath-miR156d												
GTTGACAGAAGAGAGTGAGCAC	3	11	7	0	0	8	4	0	0	0	0	0
>+3 ath-miR156d												
AGTTGACAGAAGAGAGTGAGCAC	0	0	1	0	0	0	0	0	0	0	0	0
>ath-MIR156e MI0000182<br>Arabidopsis thaliana miR156e<br>stem-loop												
AGGAGGTGACAGAAGAGAGTGAGCACACA<br>TGGTGGTTTCTTGCATGCTTTTTTGATTA<br>GGGTTTCATGCTTGAAGCTATGTGTGCTT<br>ACTCTCTCTCTGTCACCCCT	0	0	0	0	0	0	0	0	0	0	0	0
>ath-miR156e MI0000182<br>Arabidopsis thaliana miR156e<br>stem-loop												
TGACAGAAGAGAGTGAGCAC	27527	68907	69065	63209	21950	74179	58692	7	12	1	0	1
>+1 ath-miR156e												
GTGACAGAAGAGAGTGAGCAC	5	6	11	5	4	4	2	0	0	0	0	0
>+2 ath-miR156e												
GGTGACAGAAGAGAGTGAGCAC	0	0	0	0	0	0	0	0	0	0	0	0
>+3 ath-miR156e												
AGGTGACAGAAGAGAGTGAGCAC	0	0	0	0	0	0	0	0	0	0	0	0
>ath-MIR156f MI0000183<br>Arabidopsis thaliana miR156f<br>stem-loop												
GAGTGGTGAGGAATTGATGGTGACAGAAG<br>AGAGTGAGCACACATGGTGGCTTTCTTGC<br>ATATTTGAAGGTTCCATGCTTGAAGCTAT<br>GTGTGCTCACTCTCTATCCGTCACCCCCT<br>TCTCTCCCTCTCCCTC	0	0	0	0	0	0	0	0	0	0	0	0
>ath-miR156f MI0000183<br>Arabidopsis thaliana miR156f<br>stem-loop												
TGACAGAAGAGAGTGAGCAC	27527	68907	69065	63209	21950	74179	58692	7	12	1	0	1
>+1 ath-miR156f												
GTGACAGAAGAGAGTGAGCAC	5	6	11	5	4	4	2	0	0	0	0	0
>+2 ath-miR156f												
GGTGACAGAAGAGAGTGAGCAC	0	0	0	0	0	0	0	0	0	0	0	0
>+3 ath-miR156f												
TGGTGACAGAAGAGAGTGAGCAC	0	0	0	0	0	0	0	0	0	0	0	0
>ath-MIR156g MI0001082<br>Arabidopsis thaliana miR156g<br>stem-loop												
ATAACGAAGGCGACAGAAGAGAGTGAGCA<br>CACATGGCTCTTTTTCTAGCATGCTCATG<br>CTCGAAAGCTCTGCGTGCTTACTCTCTTC<br>TTGTCTCCTGCTCTCT	0	0	0	0	0	0	0	0	0	0	0	0
>ath-miR156g MI0001082<br>Arabidopsis thaliana miR156g<br>stem-loop												
CGACAGAAGAGAGTGAGCAC	14	43	41	2568	198	47	31	0	0	0	0	0
>+1 ath-miR156g												
GCGACAGAAGAGAGTGAGCAC	0	0	0	8	0	0	0	0	0	0	0	0
>+2 ath-miR156g												
GGCGACAGAAGAGAGTGAGCAC	0	0	0	0	0	0	0	0	0	0	0	0
>+3 ath-miR156g												
AGGCGACAGAAGAGAGTGAGCAC	0	0	0	0	0	0	0	0	0	0	0	0
>ath-MIR156h MI0001083<br>Arabidopsis thaliana miR156h<br>stem-loop												
ATGAAAAATGTTGACAGAAGAAAGAGAGC<br>ACAACCTGGGATTAGCAAAAAGATAGTTT<br>TGCCCTTGTCGGGAGTGTGCTCTCTTTCC<br>TTCTGCCACCATCATTGCG	0	0	0	0	0	0	0	0	0	0	0	0
>ath-miR156h MI0001083<br>Arabidopsis thaliana miR156h<br>stem-loop												
TGACAGAAGAAAGAGAGCAC	964	10850	11152	45	71	144	107	0	0	1	0	0
>+1 ath-miR156h												
TTGACAGAAGAAAGAGAGCAC	2451	25609	25928	55	71	414	329	0	0	0	0	1
>+2 ath-miR156h												
GTTGACAGAAGAAAGAGAGCAC	2	1	7	0	0	0	0	0	0	0	0	0
>+3 ath-miR156h												
TGTTGACAGAAGAAAGAGAGCAC	0	0	0	0	0	0	0	0	0	0	0	0
>ath-MIR157a MI0000184<br>Arabidopsis thaliana miR157a<br>stem-loop												
GTGTTGACAGAAGATAGAGAGCACAGATG<br>ATGAGATACAATTCGGAGCATGTTCTTTG<br>CATCTTACTCCTTTGTGCTCTCTAGCCTT<br>CTGTCATCACC	0	0	0	0	0	0	0	0	0	0	0	0

>ath-miR157a MI0000184<br>Arabidopsis thaliana miR157a<br>stem-loop												
TTGACAGAAGATAGAGAGCAC	18044	53707	54064	5173	2090	175454	151539	3	40	2	0	3
>+1 ath-miR157a												
GTTGACAGAAGATAGAGAGCAC	0	10	11	0	0	4	6	0	0	0	0	0
>+2 ath-miR157a												
TGTTGACAGAAGATAGAGAGCAC	0	1	0	0	0	0	1	0	0	0	0	0
>+3 ath-miR157a												
GTGTTGACAGAAGATAGAGAGCAC	0	2	2	0	2	1	0	0	0	0	0	0
>ath-MIR157b MI0000185<br>Arabidopsis thaliana miR157b<br>stem-loop												
TGGGAGGCATTGATAGTGTTGACAGAAGA<br>TAGAGAGCACAGATGATAAGATACAATTC<br>CTCGCAGCTTCTTTGCATCTTACTCCTTT<br>GTGCTCTCTAGCCTTCTGTCATCACCCGT<br>TATTTGCCATCACCCA	0	0	0	0	0	0	0	0	0	0	0	0
>ath-miR157b MI0000185<br>Arabidopsis thaliana miR157b<br>stem-loop												
TTGACAGAAGATAGAGAGCAC	18044	53707	54064	5173	2090	175454	151539	3	40	2	0	3
>+1 ath-miR157b												
GTTGACAGAAGATAGAGAGCAC	0	10	11	0	0	4	6	0	0	0	0	0
>+2 ath-miR157b												
TGTTGACAGAAGATAGAGAGCAC	0	1	0	0	0	0	1	0	0	0	0	0
>+3 ath-miR157b												
GTGTTGACAGAAGATAGAGAGCAC	0	2	2	0	2	1	0	0	0	0	0	0
>ath-MIR157c MI0000186<br>Arabidopsis thaliana miR157c<br>stem-loop												
AGGTTTGAGAGTGATGTTGGTTGTTGACA<br>GAAGATAGAGAGCACTAAGGATGACATGC<br>AAGTACATACATATATATCATCACACCGC<br>ATGTGGATGATAAAATATGTATAACAAAT<br>TCAAAGAAAGAGAGGGAGAGAAAGAGAGA<br>GAACCTGCATCTCTACTCTTTTGTGCTCT<br>CTATACTTCTGTCACCACCTTTATCTCTT<br>CTTCTCTCTAACCT	0	0	0	0	0	0	0	0	0	0	0	0
>ath-miR157c MI0000186<br>Arabidopsis thaliana miR157c<br>stem-loop												
TTGACAGAAGATAGAGAGCAC	18044	53707	54064	5173	2090	175454	151539	3	40	2	0	3
>+1 ath-miR157c												
GTTGACAGAAGATAGAGAGCAC	0	10	11	0	0	4	6	0	0	0	0	0
>+2 ath-miR157c												
TGTTGACAGAAGATAGAGAGCAC	0	1	0	0	0	0	1	0	0	0	0	0
>+3 ath-miR157c												
TTGTTGACAGAAGATAGAGAGCAC	0	0	0	0	1	0	1	0	0	0	0	0
>ath-MIR157d MI0000187<br>Arabidopsis thaliana miR157d<br>stem-loop												
GTGGAGGGTGATAGTGTGGTTGCTGACAG<br>AAGATAGAGAGCACTAAGGATGCTATGCA<br>AAACAGACACAGATATGTGTTTCTAATTG<br>TATTTCATACTTTAACCTCAAAGTTGATA<br>TAAAAAAAGAAAGAAAGATAGAAGAGCTA<br>GAAGACTATCTGCATCTCTATTCCTATGT<br>GCTCTCTATGCTTCTGTCATCACCTTTCT<br>TTCTCTATTTCTCTCTAC	0	0	0	0	0	0	0	0	0	0	0	0
>ath-miR157d MI0000187<br>Arabidopsis thaliana miR157d<br>stem-loop												
TGACAGAAGATAGAGAGCAC	4566	6962	7158	154	85	16170	12733	0	0	0	0	0
>+1 ath-miR157d												
CTGACAGAAGATAGAGAGCAC	3587	14318	14032	0	1	480	373	2	1	0	0	1
>+2 ath-miR157d												
GCTGACAGAAGATAGAGAGCAC	1	1	1	0	0	1	0	0	0	0	0	0
>+3 ath-miR157d												
TGCTGACAGAAGATAGAGAGCAC	0	0	0	0	0	0	0	0	0	0	0	0
>ath-MIR158a MI0000188<br>Arabidopsis thaliana miR158a<br>stem-loop												
ACACGTCATCTCTGTGCTTCTTTGTCTAC<br>AATTTTGGAAAAAGTGATGACGCCATTGC<br>TCTTTCCCAAATGTAGACAAAGCAATACC<br>GTGATGATGTCGT	0	0	0	0	0	0	0	0	0	0	0	0
>ath-miR158a MI0000188<br>Arabidopsis thaliana miR158a<br>stem-loop												
TCCCAAATGTAGACAAAGCA	18950	158670	159755	292620	246075	294934	310352	14	68	4	0	6
>+1 ath-miR158a	1.2%	0.9%	1.0%	1.3%	1.3%	1.6%	1.5%	7.1%	4.4%	0.0%	#DIV/0!	33.3%
TTCCCAAATGTAGACAAAGCA	236	1492	1564	3935	3178	4581	4758	1	3	0	0	2
>+2 ath-miR158a												
TTTCCCAAATGTAGACAAAGCA	0	10	7	18	20	11	24	0	0	0	0	0
>+3 ath-miR158a												
CTTTCCCAAATGTAGACAAAGCA	0	14	6	14	7	9	7	0	0	0	0	0

>ath-MIR158b MI0001084<br>Arabidopsis thaliana miR158b<br>stem-loop												
ATCTCTGTGCTTCTTTGTCTACACTTTTG<br>GAAAAGGTGATGATATCATTGCTTTTCCC<br>CAAATGTAGACAAAGCAATACCGTGAT	0	0	0	0	0	0	0	0	0	0	0	0
>ath-miR158b MI0001084<br>Arabidopsis thaliana miR158b<br>stem-loop												
CCCCAAATGTAGACAAAGCA	59	292	285	1742	1728	184	194	0	0	0	0	0
>+1 ath-miR158b												
TCCCCAAATGTAGACAAAGCA	107	295	276	4202	3743	140	197	0	1	0	0	0
>+2 ath-miR158b												
TTCCCCAAATGTAGACAAAGCA	0	0	0	4	1	0	0	0	0	0	0	0
>+3 ath-miR158b												
TTTCCCCAAATGTAGACAAAGCA	0	0	0	0	0	0	0	0	0	0	0	0
>ath-MIR159a MI0000189<br>Arabidopsis thaliana miR159a<br>stem-loop												
GTAGAGCTCCTTAAAGTTCAAACATGAGT<br>TGAGCAGGGTAAAGAAAAGCTGCTAAGCT<br>ATGGATCCCATAAGCCCTAATCCTTGTAA<br>AGTAAAAAAGGATTTGGTTATATGGATTG<br>CATATCTCAGGAGCTTTAACTTGCCCTTT<br>AATGGCTTTTACTCTTCTTTGGATTGAAG<br>GGAGCTCTAC	0	0	0	0	0	0	0	0	0	0	0	0
>ath-miR159a MI0000189<br>Arabidopsis thaliana miR159a<br>stem-loop												
TTTGGATTGAAGGGAGCTCTA	169	888	884	670	424	882	1645	340	384	257	33	611
>+1 ath-miR159a												
CTTTGGATTGAAGGGAGCTCTA	0	0	1	1	0	2	0	1	0	1	0	2
>+2 ath-miR159a												
TCTTTGGATTGAAGGGAGCTCTA	0	0	0	0	0	0	0	0	0	0	0	0
>+3 ath-miR159a												
TTCTTTGGATTGAAGGGAGCTCTA	0	0	0	0	0	0	0	0	0	0	0	0
>ath-MIR159b MI0000218<br>Arabidopsis thaliana miR159b<br>stem-loop												
GGAAGAGCTCCTTGAAGTTCAATGGAGGG<br>TTTAGCAGGGTGAAGTAAAGCTGCTAAGC<br>TATGGATCCCATAAGCCTTATCAAATTCA<br>ATATAATTGATGATAAGGTTTTTTTTATG<br>GATGCCATATCTCAGGAGCTTTCACTTAC<br>CCCTTTAATGGCTTCACTCTTCTTTGGAT<br>TGAAGGGAGCTCTTCATCTCTC	0	0	0	0	0	0	0	0	0	0	0	0
>ath-miR159b MI0000218<br>Arabidopsis thaliana miR159b<br>stem-loop												
TTTGGATTGAAGGGAGCTCTT	169	888	884	670	424	882	1645	340	384	257	33	611
>+1 ath-miR159b												
CTTTGGATTGAAGGGAGCTCTT	0	0	1	1	0	2	0	1	0	1	0	2
>+2 ath-miR159b												
TCTTTGGATTGAAGGGAGCTCTT	0	0	0	0	0	0	0	0	0	0	0	0
>+3 ath-miR159b												
TTCTTTGGATTGAAGGGAGCTCTT	0	0	0	0	0	0	0	0	0	0	0	0
>ath-MIR159c MI0001085<br>Arabidopsis thaliana miR159c<br>stem-loop												
GTGTAACAGAAGGAGCTCCCTTCCTCCAA<br>AACGAAGAGGACAAGATTTGAGGAACTAA<br>AATGCAGAATCTAAGAGTTCATGTCTTCC<br>TCATAGAGAGTGCGCGGTGTTAAAAGCTT<br>GAAGAAAGCACACTTTAAGGGGATTGCAC<br>GACCTCTTAGATTCTCCCTCTTTCTCTAC<br>ATATCATTCTCTTCTCTTCGTTTGGATTG<br>AAGGGAGCTCCTTTTCTTCTTC	0	0	0	0	0	0	0	0	0	0	0	0
>ath-miR159c MI0001085<br>Arabidopsis thaliana miR159c<br>stem-loop												
TTTGGATTGAAGGGAGCTCCT	1	1	0	1	4	1	0	0	1	0	0	1
>+1 ath-miR159c												
GTTTGGATTGAAGGGAGCTCCT	0	0	0	0	0	0	0	0	0	0	0	0
>+2 ath-miR159c												
CGTTTGGATTGAAGGGAGCTCCT	0	0	0	0	0	0	0	0	0	0	0	0
>+3 ath-miR159c												
TCGTTTGGATTGAAGGGAGCTCCT	0	0	0	0	0	0	0	0	0	0	0	0
>ath-MIR160a MI0000190<br>Arabidopsis thaliana miR160a<br>stem-loop												
GTATGCCTGGCTCCCTGTATGCCATATGC<br>TGAGCCCATCGAGTATCGATGACCTCCGT<br>GGATGGCGTATGAGGAGCCATGCATAT	0	0	0	0	0	0	0	0	0	0	0	0

>ath-miR160a MI0000190<br>Arabidopsis thaliana miR160a<br>stem-loop												
TGCCTGGCTCCCTGTATGCCA	470	236	239	1058	1312	793	793	5	11	4	1	19
>+1 ath-miR160a												
ATGCCTGGCTCCCTGTATGCCA	32	43	48	127	174	113	141	0	1	0	1	1
>+2 ath-miR160a												
TATGCCTGGCTCCCTGTATGCCA	2	0	0	0	2	0	2	0	0	0	0	0
>+3 ath-miR160a												
GTATGCCTGGCTCCCTGTATGCCA	0	0	0	0	0	0	0	0	0	0	0	0
>ath-MIR160b MI0000191<br>Arabidopsis thaliana miR160b<br>stem-loop												
GTCGTGCCTGGCTCCCTGTATGCCACAAG<br>AAAACATCGATTTAGTTTCAAAATCGATC<br>ACTAGTGGCGTACAGAGTAGTCAAGCATG<br>AC	0	0	0	0	0	0	0	0	0	0	0	0
>ath-miR160b MI0000191<br>Arabidopsis thaliana miR160b<br>stem-loop												
TGCCTGGCTCCCTGTATGCCA	470	236	239	1058	1312	793	793	5	11	4	1	19
>+1 ath-miR160b												
GTGCCTGGCTCCCTGTATGCCA	1	1	1	1	1	0	0	0	0	0	0	0
>+2 ath-miR160b												
CGTGCCTGGCTCCCTGTATGCCA	0	0	0	0	0	0	0	0	0	0	0	0
>+3 ath-miR160b												
TCGTGCCTGGCTCCCTGTATGCCA	0	0	0	0	0	0	0	0	0	0	0	0
>ath-MIR160c MI0000192<br>Arabidopsis thaliana miR160c<br>stem-loop												
GTTATGCCTGGCTCCCTGTATGCCACGAG<br>TGGATACCGATTTTGGTTTTAAAATCGGC<br>TGCCGGTGGCGTACAAGGAGTCAAGCATG<br>AC	0	0	0	0	0	0	0	0	0	0	0	0
>ath-miR160c MI0000192<br>Arabidopsis thaliana miR160c<br>stem-loop												
TGCCTGGCTCCCTGTATGCCA	470	236	239	1058	1312	793	793	5	11	4	1	19
>+1 ath-miR160c												
ATGCCTGGCTCCCTGTATGCCA	32	43	48	127	174	113	141	0	1	0	1	1
>+2 ath-miR160c												
TATGCCTGGCTCCCTGTATGCCA	2	0	0	0	2	0	2	0	0	0	0	0
>+3 ath-miR160c												
TTATGCCTGGCTCCCTGTATGCCA	0	0	0	0	0	0	0	0	0	0	0	0
>ath-MIR161 MI0000193<br>Arabidopsis thaliana miR161<br>stem-loop												
TGCTTGATCTCGGTTTTTGACCAGTTTAT<br>TGCGTCGATCAATGCATTGAAAGTGACTA<br>CATCGGGGTTCCGATTTTTTTTGTTCTTC<br>ATATGATGAAGCGGAAACAGTAATCAACC<br>CTGGTTTAGTCACTTTCACTGCATTAATC<br>AATGCATTTGTAAAAAGAGGGAAAAGCA	0	0	0	0	0	0	0	0	0	0	0	0
>ath-miR161.1 MI0000193<br>Arabidopsis thaliana miR161<br>stem-loop												
TGAAAGTGACTACATCGGGGT	509	1874	1882	328	296	686	577	22	91	13	16	37
>+1 ath-miR161.1												
TTGAAAGTGACTACATCGGGGT	3281	27280	26618	6558	5414	7777	7284	34	145	8	27	39
>+2 ath-miR161.1												
ATTGAAAGTGACTACATCGGGGT	1	2	1	7	2	0	5	0	0	0	0	0
>+3 ath-miR161.1												
CATTGAAAGTGACTACATCGGGGT	0	0	0	0	0	0	0	0	0	0	0	0
>ath-MIR161 MI0000193<br>Arabidopsis thaliana miR161<br>stem-loop												
TGCTTGATCTCGGTTTTTGACCAGTTTAT<br>TGCGTCGATCAATGCATTGAAAGTGACTA<br>CATCGGGGTTCCGATTTTTTTTGTTCTTC<br>ATATGATGAAGCGGAAACAGTAATCAACC<br>CTGGTTTAGTCACTTTCACTGCATTAATC<br>AATGCATTTGTAAAAAGAGGGAAAAGCA	0	0	0	0	0	0	0	0	0	0	0	0
>ath-miR161.2 MI0000193<br>Arabidopsis thaliana miR161<br>stem-loop												
TCAATGCATTGAAAGTGACTA	3060	6002	5775	5961	5937	7111	8189	35	23	3	5	11
>+1 ath-miR161.2												
ATCAATGCATTGAAAGTGACTA	3	3	1	3	0	2	4	0	0	0	0	0
>+2 ath-miR161.2												
GATCAATGCATTGAAAGTGACTA	0	0	0	0	0	0	0	0	0	0	0	0
>+3 ath-miR161.2												
CGATCAATGCATTGAAAGTGACTA	0	0	0	0	0	0	0	0	0	0	0	0
>ath-MIR162a MI0000194<br>Arabidopsis thaliana miR162a<br>stem-loop												

TAGTTGGAAGAAGAGTGAGAGTCGCTGGAGGCAGCGGTTCATCGATCTCTTCCTGTGAACACATTAAAAATGTAAAAGCATGAATAGATCGATAAACCTCTGCATCCAGCGTTTGCCTCTTGTATCTTTCTTATTGACTT	0	0	0	0	0	0	0	0	0	0	0	0
>ath-miR162a MI0000194 Arabidopsis thaliana miR162a stem-loop												
TCGATAAACCTCTGCATCCAG	274	1759	1582	548	471	1680	1056	5	17	4	2	23
>+1 ath-miR162a												
ATCGATAAACCTCTGCATCCAG	0	5	6	5	3	6	6	0	0	0	0	1
>+2 ath-miR162a												
GATCGATAAACCTCTGCATCCAG	0	0	0	0	0	0	0	0	0	0	0	0
>+3 ath-miR162a												
AGATCGATAAACCTCTGCATCCAG	0	0	0	0	0	0	0	0	0	0	0	0
>ath-MIR162b MI0000195 Arabidopsis thaliana miR162b stem-loop												
GAGTGAAGTCGCTGGAGGCAGCGGTTCATCGATCAATTCCTGTGAATATTTATTTTTGTTTACAAAAGCAAGAATCGATCGATAAACCTCTGCATCCAGCGCTGCTTGCTC	0	0	0	0	0	0	0	0	0	0	0	0
>ath-miR162b MI0000195 Arabidopsis thaliana miR162b stem-loop												
TCGATAAACCTCTGCATCCAG	274	1759	1582	548	471	1680	1056	5	17	4	2	23
>+1 ath-miR162b												
ATCGATAAACCTCTGCATCCAG	0	5	6	5	3	6	6	0	0	0	0	1
>+2 ath-miR162b												
GATCGATAAACCTCTGCATCCAG	0	0	0	0	0	0	0	0	0	0	0	0
>+3 ath-miR162b												
CGATCGATAAACCTCTGCATCCAG	0	0	0	0	0	0	0	0	0	0	0	0
>ath-MIR163 MI0000196 Arabidopsis thaliana miR163 stem-loop												
ACCCGGTGGATAAAATCGAGTTCCAACCTCTTCAACGACAACGATTTCAACACTCTCTTCCAGGAACAACTTCCTCCAGGCAGATGATACTAAAGTGCTGGAGTTCCCGGTTCCTGAGAGTGAGTCCATATCAAAATGCGCATTCGTTATCACTTGGTTGAACCCATTTGGGGATTTAAATTTGGAGGTGAAATGGAACGCGTAATTGATGACTCCTACGTGGAACCTCTTCTTAGGAAGAGCACGGTCGAAGAAGTAACTGCGCAGTGCTTAAATCGTAGATGCTAAAGTCGTTGAAGAGGACTTGGAACTTCGATATTATCCCCCGTGT	0	0	0	0	0	0	0	0	0	0	0	0
>ath-miR163 MI0000196 Arabidopsis thaliana miR163 stem-loop												
TTGAAGAGGACTTGGAACTTCGAT	89	176	160	55	39	153	137	208	88	51	0	211
>+1 ath-miR163												
GTTGAAGAGGACTTGGAACTTCGAT	0	0	0	0	0	0	0	0	0	0	0	0
>+2 ath-miR163												
CGTTGAAGAGGACTTGGAACTTCGAT	0	0	0	0	0	0	0	0	0	0	0	0
>+3 ath-miR163												
TCGTTGAAGAGGACTTGGAACTTCGAT	0	0	0	0	0	0	0	0	0	0	0	0
>ath-MIR164a MI0000197 Arabidopsis thaliana miR164a stem-loop												
GGGTGAGAATCTCCATGTTGGAGAAGCAGGGCACGTGCAAACCAACAAACACGAAATCCGTCTCATTTGCTTATTTGCACGTACTTAACTTCTCCAACATGAGCTCTTCACCC	0	0	0	0	0	0	0	0	0	0	0	0
>ath-miR164a MI0000197 Arabidopsis thaliana miR164a stem-loop												
TGGAGAAGCAGGGCACGTGCA	1999	14530	13988	4188	2833	6025	4340	6	2	2	0	12
>+1 ath-miR164a												
TTGGAGAAGCAGGGCACGTGCA	1	3	3	0	0	0	0	0	0	0	0	0
>+2 ath-miR164a												
GTTGGAGAAGCAGGGCACGTGCA	0	0	0	0	0	0	0	0	0	0	0	0
>+3 ath-miR164a												
TGTTGGAGAAGCAGGGCACGTGCA	0	0	0	0	0	0	0	0	0	0	0	0
>ath-MIR164b MI0000198 Arabidopsis thaliana miR164b stem-loop												
GATGGAGAAGCAGGGCACGTGCATTACTAGCTCATATATACACTCTCACCACAAATGCGTGTATATATGCGGAATTTTGTGATATAGATGTGTGTGTGTGTTGAGTGTGATGATATGGATGAGTTAGTTCTTCATGTGCCCATCTTCACCATC	0	0	0	0	0	0	0	0	0	0	0	0
>ath-miR164b MI0000198 Arabidopsis thaliana miR164b stem-loop												
TGGAGAAGCAGGGCACGTGCA	1999	14530	13988	4188	2833	6025	4340	6	2	2	0	12

>+1 ath-miR164b												
ATGGAGAAGCAGGGCACGTGCA	0	1	0	1	1	0	1	0	0	0	0	0
>+2 ath-miR164b												
GATGGAGAAGCAGGGCACGTGCA	0	0	0	0	0	0	0	0	0	0	0	0
>ath-MIR164c MI0001087<br>Arabidopsis thaliana miR164c<br>stem-loop												
TAACACTTGATGGAGAAGCAGGGCACGTG<br>CGAACACAAATGAAATCGATCGGTACTTG<br>TTGATCATATTTTCGCACGTGTTCTACTA<br>CTCCAACACGTGTCT	0	0	0	0	0	0	0	0	0	0	0	0
>ath-miR164c MI0001087<br>Arabidopsis thaliana miR164c<br>stem-loop												
TGGAGAAGCAGGGCACGTGCG	1999	14530	13988	4188	2833	6025	4340	6	2	2	0	12
>+1 ath-miR164c												
ATGGAGAAGCAGGGCACGTGCG	0	1	0	1	1	0	1	0	0	0	0	0
>+2 ath-miR164c												
GATGGAGAAGCAGGGCACGTGCG	0	0	0	0	0	0	0	0	0	0	0	0
>+3 ath-miR164c												
TGATGGAGAAGCAGGGCACGTGCG	0	0	0	0	0	0	0	0	0	0	0	0
>ath-MIR165a MI0000199<br>Arabidopsis thaliana miR165a<br>stem-loop												
GTTGAGGGGAATGTTGTCTGGATCGAGGA<br>TATTATAGATATATACATGTGTATGTTAA<br>TGATTCAAGTGATCATAGAGAGTATCCTC<br>GGACCAGGCTTCATCCCCCCCAAC	0	0	0	0	0	0	0	0	0	0	0	0
>ath-miR165a MI0000199<br>Arabidopsis thaliana miR165a<br>stem-loop												
TCGGACCAGGCTTCATCCCCC	8416	11026	10580	23963	24528	6250	3691	28	37	9	5	54
>+1 ath-miR165a	0.3%	0.4%	0.4%	0.9%	1.1%	0.3%	0.7%	0.0%	0.0%	11.1%	0.0%	3.7%
CTCGGACCAGGCTTCATCCCCC	25	46	46	218	275	18	26	0	0	1	0	2
>+2 ath-miR165a												
CCTCGGACCAGGCTTCATCCCCC	1	0	0	1	1	0	0	0	0	0	0	0
>+3 ath-miR165a												
TCCTCGGACCAGGCTTCATCCCCC	4	0	0	13	5	1	1	0	0	0	0	0
>ath-MIR165b MI0000200<br>Arabidopsis thaliana miR165b<br>stem-loop												
TGAAGAGGCTATTTCTGTTGTGGGGAATG<br>TTGTTTGGATCGAGGATATCATAAACGCA<br>TACACATGTTTATATGTTATGATGCATTA<br>TATGACTGATGTAATGTACATATATATAC<br>ATACATGCCACATGGTATCGTCGGACCAG<br>GCTTCATCCCCCTCAACATGTTAATTGCC<br>TTCAATCA	0	0	0	0	0	0	0	0	0	0	0	0
>ath-miR165b MI0000200<br>Arabidopsis thaliana miR165b<br>stem-loop												
TCGGACCAGGCTTCATCCCCC	8416	11026	10580	23963	24528	6250	3691	28	37	9	5	54
>+1 ath-miR165b												
GTCGGACCAGGCTTCATCCCCC	0	1	0	0	0	0	0	0	0	0	0	0
>+2 ath-miR165b												
CGTCGGACCAGGCTTCATCCCCC	0	0	0	0	0	0	0	0	0	0	0	0
>+3 ath-miR165b												
TCGTCGGACCAGGCTTCATCCCCC	0	0	0	0	0	0	0	0	0	0	0	0
>ath-MIR166a MI0000201<br>Arabidopsis thaliana miR166a<br>stem-loop												
AGGGGCTTTCTCTTTTGAGGGGACTGTTG<br>TCTGGCTCGAGGACTCTGGCTCGCTCTAT<br>TCATGTTGGATCTCTTTCGATCTAACAAT<br>CGAATTGAACCTTCAGATTTCAGATTTGA<br>TTAGGGTTTTAGCGTCTTCGGACCAGGCT<br>TCATTCCCCCCAATTGTTGCTCCCT	0	0	0	0	0	0	0	0	0	0	0	0
>ath-miR166a MI0000201<br>Arabidopsis thaliana miR166a<br>stem-loop												
TCGGACCAGGCTTCATTCCCC	60640	67566	63698	96464	99602	77516	41568	166	263	131	15	427
>+1 ath-miR166a	0.9%	4.2%	4.1%	6.1%	4.7%	3.3%	3.8%	6.0%	1.5%	7.6%	0.0%	5.6%
TTCGGACCAGGCTTCATTCCCC	550	2855	2619	5900	4662	2582	1578	10	4	10	0	24
>+2 ath-miR166a												
CTTCGGACCAGGCTTCATTCCCC	1	0	2	10	5	1	2	0	0	0	0	0
>+3 ath-miR166a												
TCTTCGGACCAGGCTTCATTCCCC	1	0	0	1	2	1	3	0	0	0	0	0
>ath-MIR166b MI0000202<br>Arabidopsis thaliana miR166b<br>stem-loop												
TTGAGGGGACTGTTGTCTGGCTCGAGGAC<br>TCTTATTCTAATACAATCTCATTTGAATA<br>CATTCAGATCTGATGATTGATTAGGGTTT<br>TAGTGTCGTCGGACCAGGCTTCATTCCCC<br>CCAA	0	0	0	0	0	0	0	0	0	0	0	0

>ath-miR166b MI0000202<br>Arabidopsis thaliana miR166b<br>stem-loop												
TCGGACCAGGCTTCATTCCCC	60640	67566	63698	96464	99602	77516	41568	166	263	131	15	427
>+1 ath-miR166b	0.0%	0.0%	0.0%	0.0%	0.0%	0.0%	0.0%	3.0%	0.4%	0.8%	0.0%	2.1%
GTCGGACCAGGCTTCATTCCCC	16	12	27	29	27	18	7	5	1	1	0	9
>+2 ath-miR166b												
CGTCGGACCAGGCTTCATTCCCC	0	0	0	1	3	3	1	0	0	0	0	0
>+3 ath-miR166b												
TCGTCGGACCAGGCTTCATTCCCC	0	0	0	0	0	0	0	0	0	0	0	0
>ath-MIR166c MI0000203<br>Arabidopsis thaliana miR166c<br>stem-loop												
GCGATTTAGTGTTGAGAGGATTGTTGTCT<br>GGCTCGAGGTCATGAAGAAGAGAATCACT<br>CGAATTAATTTGGAAGAACAAATTAAGAA<br>AACCCTAGATGATTCTCGGACCAGGCTTC<br>ATTCCCCCTAACCTACTTATCGC	0	0	0	0	0	0	0	0	0	0	0	0
>ath-miR166c MI0000203<br>Arabidopsis thaliana miR166c<br>stem-loop												
TCGGACCAGGCTTCATTCCCC	60640	67566	63698	96464	99602	77516	41568	166	263	131	15	427
>+1 ath-miR166c	0.0%	0.0%	0.0%	0.3%	0.3%	0.1%	0.1%	0.0%	0.0%	0.0%	0.0%	0.0%
CTCGGACCAGGCTTCATTCCCC	15	24	23	277	330	69	41	0	0	0	0	0
>+2 ath-miR166c												
TCTCGGACCAGGCTTCATTCCCC	0	0	1	3	0	0	0	0	0	0	0	0
>+3 ath-miR166c												
TTCTCGGACCAGGCTTCATTCCCC	0	0	0	0	0	0	0	0	0	0	0	0
>ath-MIR166d MI0000204<br>Arabidopsis thaliana miR166d<br>stem-loop												
GGTTGAGAGGAATATTGTCTGGCTCGAGG<br>TCATGAAGAAGATCGGTAGATTGATTCAT<br>TTTAAAGAGTGAAATCCCTAAATGATTCT<br>CGGACCAGGCTTCATTCCCCCCAACC	0	0	0	0	0	0	0	0	0	0	0	0
>ath-miR166d MI0000204<br>Arabidopsis thaliana miR166d<br>stem-loop												
TCGGACCAGGCTTCATTCCCC	60640	67566	63698	96464	99602	77516	41568	166	263	131	15	427
>+1 ath-miR166d	0.0%	0.0%	0.0%	0.3%	0.3%	0.1%	0.1%	0.0%	0.0%	0.0%	0.0%	0.0%
CTCGGACCAGGCTTCATTCCCC	15	24	23	277	330	69	41	0	0	0	0	0
>+2 ath-miR166d												
TCTCGGACCAGGCTTCATTCCCC	0	0	1	3	0	0	0	0	0	0	0	0
>+3 ath-miR166d												
TTCTCGGACCAGGCTTCATTCCCC	0	0	0	0	0	0	0	0	0	0	0	0
>ath-MIR166e MI0000205<br>Arabidopsis thaliana miR166e<br>stem-loop												
TTGAGGGGAATGTTGTCTGGCACGAGGCC<br>CTTAACTTAGATCTATATTTGATTATATA<br>TATATGTCTCTTCTTTATTCATTAGTCTA<br>TACATGAATGATCATTTTACGGTTAATGA<br>CGTCGGACCAGGCTTCATTCCCCTCAA	0	0	0	0	0	0	0	0	0	0	0	0
>ath-miR166e MI0000205<br>Arabidopsis thaliana miR166e<br>stem-loop												
TCGGACCAGGCTTCATTCCCC	60640	67566	63698	96464	99602	77516	41568	166	263	131	15	427
>+1 ath-miR166e												
GTCGGACCAGGCTTCATTCCCC	16	12	27	29	27	18	7	5	1	1	0	9
>+2 ath-miR166e												
CGTCGGACCAGGCTTCATTCCCC	0	0	0	1	3	3	1	0	0	0	0	0
>+3 ath-miR166e												
ACGTCGGACCAGGCTTCATTCCCC	0	0	0	0	0	0	0	0	0	0	0	0
>ath-MIR166f MI0000206<br>Arabidopsis thaliana miR166f<br>stem-loop												
AAGTTCAGGTGAATGATGCCTGGCTCGAG<br>ACCATTCAATCTCATGATCTCATGATTAT<br>AACGATGATGATGATGATGTCGGACCAGG<br>CTTCATTCCCCTCAACTT	0	0	0	0	0	0	0	0	0	0	0	0
>ath-miR166f MI0000206<br>Arabidopsis thaliana miR166f<br>stem-loop												
TCGGACCAGGCTTCATTCCCC	60640	67566	63698	96464	99602	77516	41568	166	263	131	15	427
>+1 ath-miR166f	0.0%	0.0%	0.0%	0.0%	0.0%	0.0%	0.0%	3.0%	0.4%	0.8%	0.0%	2.1%
GTCGGACCAGGCTTCATTCCCC	16	12	27	29	27	18	7	5	1	1	0	9
>+2 ath-miR166f												
TGTCGGACCAGGCTTCATTCCCC	0	0	0	0	0	0	0	0	0	0	0	0
>+3 ath-miR166f												
ATGTCGGACCAGGCTTCATTCCCC	0	1	1	0	0	1	0	0	0	0	0	0
>ath-MIR166g MI0000207<br>Arabidopsis thaliana miR166g<br>stem-loop												

GCGATTTAGGGTTTAGAGGAATGTTGTTT<br>GGCTCGAGGTCATGGAGAGTAATTCGTTA<br>ACCCAACTCAAAACTCTAAATGATTCTCG<br>GACCAGGCTTCATTCCCCTCAACCTATTT<br>TATCGC	0	0	0	0	0	0	0	0	0	0	0	0
>ath-miR166g MI0000207<br>Arabidopsis thaliana miR166g<br>stem-loop												
TCGGACCAGGCTTCATTCCCC	60640	67566	63698	96464	99602	77516	41568	166	263	131	15	427
>+1 ath-miR166g	0.0%	0.0%	0.0%	0.3%	0.3%	0.1%	0.1%	0.0%	0.0%	0.0%	0.0%	0.0%
CTCGGACCAGGCTTCATTCCCC	15	24	23	277	330	69	41	0	0	0	0	0
>+2 ath-miR166g												
TCTCGGACCAGGCTTCATTCCCC	0	0	1	3	0	0	0	0	0	0	0	0
>+3 ath-miR166g												
TTCTCGGACCAGGCTTCATTCCCC	0	0	0	0	0	0	0	0	0	0	0	0
>ath-MIR167a MI0000208<br>Arabidopsis thaliana miR167a<br>stem-loop												
TGGTGCACCGGCATCTGATGAAGCTGCCA<br>GCATGATCTAATTAGCTTTCTTTATCCTT<br>TGTTGTGTTTCATGACGATGGTTAAGAGA<br>TCAGTCTCGATTAGATCATGTTCGCAGTT<br>TCACCCGTTGACTGTCGCACCC	0	0	0	0	0	0	0	0	0	0	0	0
>ath-miR167a MI0000208<br>Arabidopsis thaliana miR167a<br>stem-loop												
TGAAGCTGCCAGCATGATCTA	73752	217371	208400	22500	13196	134926	99827	134	1131	173	2	332
>+1 ath-miR167a												
ATGAAGCTGCCAGCATGATCTA	12	5	7	6	3	13	9	0	0	0	0	0
>+2 ath-miR167a												
GATGAAGCTGCCAGCATGATCTA	0	0	0	0	0	1	0	0	0	0	0	0
>+3 ath-miR167a												
TGATGAAGCTGCCAGCATGATCTA	0	0	0	0	0	0	0	0	0	0	0	0
>ath-MIR167b MI0000209<br>Arabidopsis thaliana miR167b<br>stem-loop												
GGGAACAAGTGAAGCTGCCAGCATGATCT<br>ATCTTTGGTTAAGAGATGAATGTGGAAAC<br>ATATTGCTTAAACCCAAGCTAGGTCATGC<br>TCTGACAGCCTCACTCCTTCCT	0	0	0	0	0	0	0	0	0	0	0	0
>ath-miR167b MI0000209<br>Arabidopsis thaliana miR167b<br>stem-loop												
TGAAGCTGCCAGCATGATCTA	73752	217371	208400	22500	13196	134926	99827	134	1131	173	2	332
>+1 ath-miR167b												
GTGAAGCTGCCAGCATGATCTA	2	0	0	0	0	0	0	0	1	0	0	0
>+2 ath-miR167b												
AGTGAAGCTGCCAGCATGATCTA	1	0	0	0	0	0	0	0	0	0	0	0
>+3 ath-miR167b												
AAGTGAAGCTGCCAGCATGATCTA	0	0	0	0	0	0	0	0	0	0	0	0
>ath-MIR167c MI0001088<br>Arabidopsis thaliana miR167c<br>stem-loop												
CCAGTAGCAGTTAAGCTGCCAGCATGATC<br>TTGTCTTCCTCTCTTAGGTTTCATATATA<br>GTTAATAAATATTTTATATATTTCTTGTT<br>CTTACAAGATTATATGATCATAGCTTAGA<br>GAGAGAGAGAGACTAGGTCATGCTGGTAG<br>TTTCACCTGCTAATG	0	0	0	0	0	0	0	0	0	0	0	0
>ath-miR167c MI0001088<br>Arabidopsis thaliana miR167c<br>stem-loop												
TAAGCTGCCAGCATGATCTTG	2	19	11	1052	677	15	9	0	2	0	0	0
>+1 ath-miR167c	0.0%	5.3%	9.1%	4.3%	3.8%	0.0%	0.0%	#####	0.0%	#####	#DIV/0!	#####
TTAAGCTGCCAGCATGATCTTG	0	1	1	45	26	0	0	0	0	0	0	0
>+2 ath-miR167c												
GTTAAGCTGCCAGCATGATCTTG	0	0	0	0	0	0	0	0	0	0	0	0
>+3 ath-miR167c												
AGTTAAGCTGCCAGCATGATCTTG	0	0	0	0	0	0	0	0	0	0	0	0
>ath-MIR167d MI0000975<br>Arabidopsis thaliana miR167d<br>stem-loop												
TGTTGGTTTTTAGAAGCTGAAGCTGCCAG<br>CATGATCTGGTAATCGCTACATACGACAT<br>ACACACATCACTAAACTTCTTTATAATTT<br>ATGCACACACATACAGCTCTTAATGGCCA<br>CAACTCAAAGTTATAATTAGTGCATGATC<br>TCTAGTTATTTGACTGCTTTTAATATATG<br>TTTATGGATTCACGCATGTGTGTGTATGT<br>ACATAATTTACATGCATGCACTTTGTGTA<br>TGGTACACATCAATTTGAACCCGTTCAAA<br>ATTCTGTTTTTATTAGTATATATATAGAT<br>GTATGTGGTGTGTGTGTCAGTGTGTGTGT<br>GTGTTTATAGATAGTAGTACTAGGTCATC<br>CTGCAGCTTCAGTCACTAAATCACCAACA	0	0	0	0	0	0	0	0	0	0	0	0

>ath-miR167d MI0000975<br>Arabidopsis thaliana miR167d<br>stem-loop												
TGAAGCTGCCAGCATGATCTGG	115	181	180	56	51	1780	2210	0	19	0	0	0
>+1 ath-miR167d												
CTGAAGCTGCCAGCATGATCTGG	0	0	1	0	1	9	22	0	0	0	0	0
>+2 ath-miR167d												
GCTGAAGCTGCCAGCATGATCTGG	0	0	0	0	0	0	0	0	0	0	0	0
>+3 ath-miR167d												
AGCTGAAGCTGCCAGCATGATCTGG	0	0	0	0	0	0	0	0	0	0	0	0
>ath-MIR168a MI0000210<br>Arabidopsis thaliana miR168a<br>stem-loop												
CACCATCGGGCTCGGATTCGCTTGGTGCA<br>GGTCGGGAACCAATTCGGCTGACACAGCC<br>TCGTGACTTTTAAACCTTTATTGGTTTGT<br>GAGCAGGGATTGGATCCCGCCTTGCATCA<br>ACTGAATCGGATCCTCGAGGTG	0	0	0	0	0	0	0	0	0	0	0	0
>ath-miR168a MI0000210<br>Arabidopsis thaliana miR168a<br>stem-loop												
TCGCTTGGTGCAGGTCGGGAA	9827	35012	33061	5537	5207	22445	21678	3	17	9	36	38
>+1 ath-miR168a	0.0%	0.0%	0.0%	0.0%	0.1%	0.0%	0.0%	0.0%	0.0%	0.0%	0.0%	0.0%
TTCGCTTGGTGCAGGTCGGGAA	1	3	4	1	3	1	5	0	0	0	0	0
>+2 ath-miR168a												
ATTCGCTTGGTGCAGGTCGGGAA	0	0	0	0	0	0	1	0	0	0	0	0
>+3 ath-miR168a												
GATTCGCTTGGTGCAGGTCGGGAA	0	0	0	0	0	0	0	0	0	0	0	0
>ath-MIR168b MI0000211<br>Arabidopsis thaliana miR168b<br>stem-loop												
TTACCGGCGGTCTCGGATTCGCTTGGTGC<br>AGGTCGGGAACTGATTGGCTGACACCGAC<br>ACGTGTCTTGTCATGGTTGGTTTGTGAGC<br>TCCCGTCTTGTATCAACTGAATCGGAGTC<br>CGAGGTGA	0	0	0	0	0	0	0	0	0	0	0	0
>ath-miR168b MI0000211<br>Arabidopsis thaliana miR168b<br>stem-loop												
TCGCTTGGTGCAGGTCGGGAA	9827	35012	33061	5537	5207	22445	21678	3	17	9	36	38
>+1 ath-miR168b												
TTCGCTTGGTGCAGGTCGGGAA	1	3	4	1	3	1	5	0	0	0	0	0
>+2 ath-miR168b												
ATTCGCTTGGTGCAGGTCGGGAA	0	0	0	0	0	0	1	0	0	0	0	0
>+3 ath-miR168b												
GATTCGCTTGGTGCAGGTCGGGAA	0	0	0	0	0	0	0	0	0	0	0	0
>ath-MIR169a MI0000212<br>Arabidopsis thaliana miR169a<br>stem-loop												
GTGACGAAAGTAGTGTGCAGCCAAGGATG<br>ACTTGCCGATTTAAATGATCTTTCTTTAT<br>ACTCTATTAAGACAATTTAGTTTCAAACT<br>TTTTTTTTTTTTTTTTTTTGAAGGATTCA<br>GGAAGAAATTAGGATATATTATTCCGTAT<br>AAAATACAAGATATATAAAACCAAAAAGA<br>AAAAGTAACATGATCGGCAAGTTGTCCTT<br>GGCTACACGTTACTTTGTGTCGC	0	0	0	0	0	0	0	0	0	0	0	0
>ath-miR169a MI0000212<br>Arabidopsis thaliana miR169a<br>stem-loop												
CAGCCAAGGATGACTTGCCGA	105	204	185	238	1030	556	982	50	266	20	0	11
>+1 ath-miR169a	5.7%	0.0%	2.2%	0.8%	2.4%	0.0%	0.9%	2.0%	1.1%	5.0%	#DIV/0!	0.0%
GCAGCCAAGGATGACTTGCCGA	6	0	4	2	25	0	9	1	3	1	0	0
>+2 ath-miR169a												
TGCAGCCAAGGATGACTTGCCGA	0	0	0	0	0	1	0	0	0	0	0	0
>+3 ath-miR169a												
GTGCAGCCAAGGATGACTTGCCGA	0	0	0	0	0	0	0	0	0	0	0	0
>ath-MIR169b MI0000976<br>Arabidopsis thaliana miR169b<br>stem-loop												
CCCAACGGAGTAGAATTGCATGAAGTGGA<br>GTAGAGTATAATGCAGCCAAGGATGACTT<br>GCCGGAACGTTGTTAACCATGCATATGAA<br>TAATGTGATGATTAATTATGTGATGAACA<br>TATTTCTGGCAAGTTGTCCTTCGGCTACA<br>TTTTGCTCTCTTCTTCTCATGCAAACTTT<br>CCTTGGG	0	0	0	0	0	0	0	0	0	0	0	0
>ath-miR169b MI0000976<br>Arabidopsis thaliana miR169b<br>stem-loop												
CAGCCAAGGATGACTTGCCGG	105	204	185	238	1030	556	982	50	266	20	0	11
>+1 ath-miR169b	5.7%	0.0%	2.2%	0.8%	2.4%	0.0%	0.9%	2.0%	1.1%	5.0%	#DIV/0!	0.0%
GCAGCCAAGGATGACTTGCCGG	6	0	4	2	25	0	9	1	3	1	0	0
>+2 ath-miR169b												
TGCAGCCAAGGATGACTTGCCGG	0	0	0	0	0	1	0	0	0	0	0	0
>+3 ath-miR169b												
ATGCAGCCAAGGATGACTTGCCGG	0	0	0	0	0	0	0	0	0	0	0	0

>ath-MIR169c MI0000977 Arabidopsis thaliana miR169c stem-loop												
TCGTCCATTATGAGTATTAATTATGGTTA<br>GGGAATCTTACAGAATGAAAATGAAGGTG<br>TGAATGGATTGTCTCATCTAAAGCCTTGA<br>ATGTGGGAAAAAGGCCATTGTTGTTCAGC<br>CAAGGATGACTTGCCGGTAGCTTGTATTA<br>TGATTACTCTATATTCGATTTATATTATG<br>GAGATGATGGTTTATATATATTTACTTAT<br>CTACATAGTTTTAGTTGATTTTTTTTCGT<br>ACGTAATATATAATACGAAAAAGTATTTACT<br>TATTTATATATGTGTGTTGGGGCAAGAAG<br>TGTAACCAAGCTAGCCCGGCAAGTCATCT<br>ATGGCTATGCAACTGTCTCTTCCTCTCAT<br>TCTAGGCTTACGATGACACGTAAAAAATC<br>CCAAATATCACTAATATGATATGAATATG<br>GATGA	0	0	0	0	0	0	0	0	0	0	0	0
>ath-miR169c MI0000977 Arabidopsis thaliana miR169c stem-loop												
CAGCCAAGGATGACTTGCCGG	105	204	185	238	1030	556	982	50	266	20	0	11
>+1 ath-miR169c												
TCAGCCAAGGATGACTTGCCGG	0	0	0	0	1	0	0	0	0	0	0	0
>+2 ath-miR169c												
TTCAGCCAAGGATGACTTGCCGG	0	0	0	0	0	0	0	0	0	0	0	0
>+3 ath-miR169c												
GTTCAGCCAAGGATGACTTGCCGG	0	0	0	0	0	0	0	0	0	0	0	0
>ath-MIR169d MI0000978 Arabidopsis thaliana miR169d stem-loop												
GTATCATAGAGTCTTGCATGGAAAAATTA<br>AAGAATGAGATTGAGCCAAGGATGACTTG<br>CCGATGTTATCAACAAATCTTAACTGATT<br>TTGGTGTCCGGCAAGTTGACCTTGGCTCT<br>GTTTCCTTCTTTTCTTTTCAATGTCAAAC<br>TCTAGATAT	0	0	0	0	0	0	0	0	0	0	0	0
>ath-miR169d MI0000978 Arabidopsis thaliana miR169d stem-loop												
TGAGCCAAGGATGACTTGCCG	1169	518	476	170	752	278	366	8	4	3	2	4
>+1 ath-miR169d												
TTGAGCCAAGGATGACTTGCCG	0	0	1	0	3	0	0	0	0	0	0	0
>+2 ath-miR169d												
ATTGAGCCAAGGATGACTTGCCG	0	0	0	0	0	1	0	0	0	0	0	0
>+3 ath-miR169d												
GATTGAGCCAAGGATGACTTGCCG	0	0	0	0	0	0	0	0	0	0	0	0
>ath-MIR169e MI0000979 Arabidopsis thaliana miR169e stem-loop												
TGATGATGATGATGAGTCACTAATTAATT<br>GTATCATAGAGTCTTGCATGGAAAAATAG<br>AAAATGAGATTGAGCCAAGGATGACTTGC<br>CGATTTTCTCAACGAATCTTACTGATTAT<br>GGTATCCGGCAAGTTGACTTTGGCTCTGT<br>TTCCTTCCCTTCTTTTCGATGTCAAACTC<br>TAGATACCTAACCACATATCATATATATC<br>ATCATCATTCATCA	0	0	0	0	0	0	0	0	0	0	0	0
>ath-miR169e MI0000979 Arabidopsis thaliana miR169e stem-loop												
TGAGCCAAGGATGACTTGCCG	1169	518	476	170	752	278	366	8	4	3	2	4
>+1 ath-miR169e												
TTGAGCCAAGGATGACTTGCCG	0	0	1	0	3	0	0	0	0	0	0	0
>+2 ath-miR169e												
ATTGAGCCAAGGATGACTTGCCG	0	0	0	0	0	1	0	0	0	0	0	0
>+3 ath-miR169e												
GATTGAGCCAAGGATGACTTGCCG	0	0	0	0	0	0	0	0	0	0	0	0
>ath-MIR169f MI0000980 Arabidopsis thaliana miR169f stem-loop												
GGGTCTTGCATGAAGGAATAACGAATGGA<br>ATTGAGCCAAGGATGACTTGCCGGTTTAA<br>ACCCAACCGGTTTATGACCATTGATTTGG<br>TCTCATTCACAATCTGTTGATTCGTGTCT<br>GGCAAGTTGACCTTGGCTCTGCTTCGTTC<br>TCTATTCTTCCATGTTAGATTC	0	0	0	0	0	0	0	0	0	0	0	0
>ath-miR169f MI0000980 Arabidopsis thaliana miR169f stem-loop												
TGAGCCAAGGATGACTTGCCG	1169	518	476	170	752	278	366	8	4	3	2	4
>+1 ath-miR169f												
TTGAGCCAAGGATGACTTGCCG	0	0	1	0	3	0	0	0	0	0	0	0
>+2 ath-miR169f												
ATTGAGCCAAGGATGACTTGCCG	0	0	0	0	0	1	0	0	0	0	0	0
>+3 ath-miR169f												
AATTGAGCCAAGGATGACTTGCCG	0	0	0	0	0	0	0	0	0	0	0	0

>ath-MIR169g MI0000981 Arabidopsis thaliana miR169g stem-loop												
TGCCTATAAATACCTTCATCACGAGTATGACAAGATCACAAGACAAGAAAAGAAAGGTAGAGAAAACATGATAATGATGATTACGATGATGAGAGTCTCTAGTTGTATCAGAGGGTCTTGCATGGAAGAATAGAGAATGAGGTTGAGCCAAGGATGACTTGCCGGGTTTTTTTACCAATGAATCTAATTAACTGATTCTGGTGTCCGGCAAGTTGACCTTGGCTCTGTTTCCTTCTCTTCTTTTGGATGTCAGACTCCAAGATATCTATCATCATGAATCGTGATCAAACTTTGTAATTTCATTGAAATGTGTTTTTCTTGATGCGAATTTTTTGGCTTACGGTTTTTCGATTTGAATGATCAGATTTTTGTTTTTGCA	0	0	0	0	0	0	0	0	0	0	0	0
>ath-miR169g MI0000981 Arabidopsis thaliana miR169g stem-loop												
TGAGCCAAGGATGACTTGCCG	1169	518	476	170	752	278	366	8	4	3	2	4
>+1 ath-miR169g												
TTGAGCCAAGGATGACTTGCCG	0	0	1	0	3	0	0	0	0	0	0	0
>+2 ath-miR169g												
GTTGAGCCAAGGATGACTTGCCG	0	0	0	0	0	0	0	0	0	0	0	0
>+3 ath-miR169g												
GGTTGAGCCAAGGATGACTTGCCG	0	0	0	0	0	0	0	0	0	0	0	0
>ath-MIR169h MI0000982 Arabidopsis thaliana miR169h stem-loop												
TCATATAAGAGAAAATGGTGACATGAAGAATGAGAACTTGTGTGGTAGCCAAGGATGACTTGCCTGCGTTTTAGACCATATATATCAAAGACTCACTCGATCGATAGTCTTAGAGTTGGTTGGTCGTCAGGCAGTCTCCTTGGCTATTCAAACAATTCTCATTCTCTTCATTCACATTTCTCTTTTTTGG	0	0	0	0	0	0	0	0	0	0	0	0
>ath-miR169h MI0000982 Arabidopsis thaliana miR169h stem-loop												
TAGCCAAGGATGACTTGCCTG	49	41	44	95	331	140	347	204	246	100	0	6
>+1 ath-miR169h												
GTAGCCAAGGATGACTTGCCTG	0	0	0	1	4	1	0	0	0	0	0	0
>+2 ath-miR169h												
GGTAGCCAAGGATGACTTGCCTG	0	1	0	1	6	0	1	2	0	0	0	0
>+3 ath-miR169h												
TGGTAGCCAAGGATGACTTGCCTG	0	0	0	0	0	0	0	0	0	0	0	0
>ath-MIR169i MI0000983 Arabidopsis thaliana miR169i stem-loop												
GAAGGAGATGTCAAAGATGAATAGAAGAATCATATTTGGTAGCCAAGGATGACTTGCCTGACTCTTTGTGTAAAATGTTTAGTGTCTTGTTTGAAGTCACTATAAGTTGTATCAAGCAATGACCATTTTGCTTATAAAAAAGATATCAGGCAGTCTCCTTGGCTATCCTTATATGTTCTTCTCTTTCATCTCAGACATTCACCTTC	0	0	0	0	0	0	0	0	0	0	0	0
>ath-miR169i MI0000983 Arabidopsis thaliana miR169i stem-loop												
TAGCCAAGGATGACTTGCCTG	49	41	44	95	331	140	347	204	246	100	0	6
>+1 ath-miR169i												
GTAGCCAAGGATGACTTGCCTG	0	0	0	1	4	1	0	0	0	0	0	0
>+2 ath-miR169i												
GGTAGCCAAGGATGACTTGCCTG	0	1	0	1	6	0	1	2	0	0	0	0
>+3 ath-miR169i												
TGGTAGCCAAGGATGACTTGCCTG	0	0	0	0	0	0	0	0	0	0	0	0
>ath-MIR169j MI0000984 Arabidopsis thaliana miR169j stem-loop												
GAGTATAATGAGGAAGAGAGGTCTAACATGGCGAAAAGAGTCATGTTTAGTAGCCAAGGATGACTTGCCTGATCTTTTTCACCTCCATGATTCAATTTGTAATTCATGGGTTTTGGATTATTATACATTCAAAAGTATAATAATTTGAAATCATGTTGAATCTTGCGGGTTAGGTTTCAGGCAGTCTCCTTGGCTATCTTGACATGCTTTTTTCATTCACG	0	0	0	0	0	0	0	0	0	0	0	0
>ath-miR169j MI0000984 Arabidopsis thaliana miR169j stem-loop												
TAGCCAAGGATGACTTGCCTG	49	41	44	95	331	140	347	204	246	100	0	6
>+1 ath-miR169j												
GTAGCCAAGGATGACTTGCCTG	0	0	0	1	4	1	0	0	0	0	0	0
>+2 ath-miR169j												
AGTAGCCAAGGATGACTTGCCTG	0	0	0	0	1	0	0	0	0	0	0	0
>+3 ath-miR169j												

TAGTAGCCAAGGATGACTTGCCTG	0	0	0	0	0	0	0	0	0	0	0	0
>ath-MIR169k MI0000985 Arabidopsis thaliana miR169k stem-loop												
TTAGAAGGAGATGTCAAAGATGAATAGGAGAACAATATTTGGTAGCCAAGGATGACTTGCCTGCTTCTCTGAACAAAATGGTCGATGTCATGTTTTGAAGTGACTATAAGTTATACCAAGAAATGACCATTTTGTTTATAAATAGACATCAGGCAGTCTCCTTGGCTATCCTTATATGTTCTTCTTTCTCAACTCAGATATTTACCTTCATCC	0	0	0	0	0	0	0	0	0	0	0	0
>ath-miR169k MI0000985 Arabidopsis thaliana miR169k stem-loop												
TAGCCAAGGATGACTTGCCTG	49	41	44	95	331	140	347	204	246	100	0	6
>+1 ath-miR169k												
GTAGCCAAGGATGACTTGCCTG	0	0	0	1	4	1	0	0	0	0	0	0
>+2 ath-miR169k												
GGTAGCCAAGGATGACTTGCCTG	0	1	0	1	6	0	1	2	0	0	0	0
>+3 ath-miR169k												
TGGTAGCCAAGGATGACTTGCCTG	0	0	0	0	0	0	0	0	0	0	0	0
>ath-MIR169l MI0000986 Arabidopsis thaliana miR169l stem-loop												
ATGAAGAAGAGAGGTCTAATATGGCGAAAAGAGTCATGTTTAATAGCCAAGGATGACTTGCCTGATCTTTTTCACCTCCATGATTCAATTTTAAGTTCGTGGATTTTGGATTATTATGCGTTTAAAAGGTATAATAATTTGAGATCATGTTGAATCTTGCGGGTTAGGTTTCAGGCAGTCTCTTTGGCTATCTTGACATGCTTTCTTCATC	0	0	0	0	0	0	0	0	0	0	0	0
>ath-miR169l MI0000986 Arabidopsis thaliana miR169l stem-loop												
TAGCCAAGGATGACTTGCCTG	49	41	44	95	331	140	347	204	246	100	0	6
>+1 ath-miR169l												
ATAGCCAAGGATGACTTGCCTG	0	0	0	0	1	0	0	0	0	0	0	0
>+2 ath-miR169l												
AATAGCCAAGGATGACTTGCCTG	1	0	0	0	4	3	8	0	0	0	0	0
>+3 ath-miR169l												
TAATAGCCAAGGATGACTTGCCTG	0	0	0	0	0	0	0	0	0	0	0	0
>ath-MIR169m MI0000987 Arabidopsis thaliana miR169m stem-loop												
TAGAAGGAGAAGTCAAAGATGAATAGAAGAATCATATTTGGTAGCCAAGGATGACTTGCCTGTTTCTTTGAGTAAAATGGGTTAGTGTCATGTTTGACAAGTGACTATAAGTTATATCAAGCAATGACCATTTTACTCATCAAAAGACATCAGGCAGTCTCCTTGGCTATCCTTATATGTTCTTCTCTCTCATCTCAGACGTTTACCTTCAT	0	0	0	0	0	0	0	0	0	0	0	0
>ath-miR169m MI0000987 Arabidopsis thaliana miR169m stem-loop												
TAGCCAAGGATGACTTGCCTG	49	41	44	95	331	140	347	204	246	100	0	6
>+1 ath-miR169m												
GTAGCCAAGGATGACTTGCCTG	0	0	0	1	4	1	0	0	0	0	0	0
>+2 ath-miR169m												
GGTAGCCAAGGATGACTTGCCTG	0	1	0	1	6	0	1	2	0	0	0	0
>+3 ath-miR169m												
TGGTAGCCAAGGATGACTTGCCTG	0	0	0	0	0	0	0	0	0	0	0	0
>ath-MIR169n MI0000988 Arabidopsis thaliana miR169n stem-loop												
GATGAAGAAGAGAGGTCTAACATGGCGGAAAGCGTCATGTTTAGTAGCCAAGGATGACTTGCCTGATCTTTTTCGCCTCCACGATTCAATTTCAAATTCATGCATTTTGGATTATTATACCTTTTAAAGTATAATAGGTCAAATATCATGTTGAATCTTGCGGGTTAGGTTTCAGGCAGTCTCTTTGGCTATCTTGACATGCTTTTTCCATCCAT	0	0	0	0	0	0	0	0	0	0	0	0
>ath-miR169n MI0000988 Arabidopsis thaliana miR169n stem-loop												
TAGCCAAGGATGACTTGCCTG	49	41	44	95	331	140	347	204	246	100	0	6
>+1 ath-miR169n												
GTAGCCAAGGATGACTTGCCTG	0	0	0	1	4	1	0	0	0	0	0	0
>+2 ath-miR169n												
AGTAGCCAAGGATGACTTGCCTG	0	0	0	0	1	0	0	0	0	0	0	0
>+3 ath-miR169n												
TAGTAGCCAAGGATGACTTGCCTG	0	0	0	0	0	0	0	0	0	0	0	0
>ath-MIR170 MI0000213 Arabidopsis thaliana miR170 stem-loop												

CGAGAGAGTCCCTCTGATATTGGCCTGGTTCACTCAGATTCTCTTTTACTAACTCATCTGATTGAGCCGTGTCAATATCTCAGTCCTCTCTCG	0	0	0	0	0	0	0	0	0	0	0	0
>ath-miR170 MI0000213 Arabidopsis thaliana miR170 stem-loop												
TGATTGAGCCGTGTCAATATC	44	9	11	2	0	25	17	94	115	52	0	62
>+1 ath-miR170												
CTGATTGAGCCGTGTCAATATC	0	0	0	0	0	0	0	0	0	0	0	0
>+2 ath-miR170												
TCTGATTGAGCCGTGTCAATATC	0	0	0	0	0	0	0	0	0	0	0	0
>+3 ath-miR170												
ATCTGATTGAGCCGTGTCAATATC	0	0	0	0	0	0	0	0	0	0	0	0
>ath-MIR171a MI0000214 Arabidopsis thaliana miR171a stem-loop												
ATGAGAGAGTCCCTTTGATATTGGCCTGGTTCACTCAGATCTTACCTGACCACACACGTAGATATACATTATTCTCTCTAGATTATCTGATTGAGCCGCGCCAATATCTCAGTACTCTCTCGT	0	0	0	0	0	0	0	0	0	0	0	0
>ath-miR171a MI0000214 Arabidopsis thaliana miR171a stem-loop												
TGATTGAGCCGCGCCAATATC	1197	597	590	0	1	246	254	68	19	74	2	72
>+1 ath-miR171a												
CTGATTGAGCCGCGCCAATATC	0	0	0	0	0	0	0	0	0	0	0	0
>+2 ath-miR171a												
TCTGATTGAGCCGCGCCAATATC	1	0	0	0	0	0	0	0	0	0	0	0
>+3 ath-miR171a												
ATCTGATTGAGCCGCGCCAATATC	0	0	0	0	0	0	0	0	0	0	0	0
>ath-MIR171b MI0000989 Arabidopsis thaliana miR171b stem-loop												
TGCAAGGTAACGCGAGATATTAGTGCGGTTCAATCAAATAGTCGTCCTCTTAACTCATGGAGAACGGTGTTGTTCGATTGAGCCGTGCCAATATCACGCGGTAAACCAAAAATGGCA	0	0	0	0	0	0	0	0	0	0	0	0
>ath-miR171b MI0000989 Arabidopsis thaliana miR171b stem-loop												
TTGAGCCGTGCCAATATCACG	20	128	103	110	58	88	98	23	7	3	8	26
>+1 ath-miR171b												
ATTGAGCCGTGCCAATATCACG	0	1	0	0	0	0	0	0	0	0	0	0
>+2 ath-miR171b												
GATTGAGCCGTGCCAATATCACG	0	0	0	0	0	0	0	0	0	0	0	0
>+3 ath-miR171b												
CGATTGAGCCGTGCCAATATCACG	0	0	0	0	0	0	0	0	0	0	0	0
>ath-MIR171c MI0000990 Arabidopsis thaliana miR171c stem-loop												
TGAGCGCACTATCGGACATCAAATACGAGATATTGGTGCGGTTCAATCAGAAAACCGTACTCTTTTGTTTTAAAGATCGGTTTATTTGATTGAGCCGTGCCAATATCACGCGTTTA	0	0	0	0	0	0	0	0	0	0	0	0
>ath-miR171c MI0000990 Arabidopsis thaliana miR171c stem-loop												
TTGAGCCGTGCCAATATCACG	20	128	103	110	58	88	98	23	7	3	8	26
>+1 ath-miR171c												
ATTGAGCCGTGCCAATATCACG	0	1	0	0	0	0	0	0	0	0	0	0
>+2 ath-miR171c												
GATTGAGCCGTGCCAATATCACG	0	0	0	0	0	0	0	0	0	0	0	0
>+3 ath-miR171c												
TGATTGAGCCGTGCCAATATCACG	0	0	0	0	0	0	0	0	0	0	0	0
>ath-MIR172a MI0000215 Arabidopsis thaliana miR172a stem-loop												
TGCTGTGGCATCATCAAGATTCACATCTGTTGATGGACGGTGGTGATTCACTCTCCACAAAGTTCTCTATGAAAATGAGAATCTTGATGATGCTGCATCGGC	0	0	0	0	0	0	0	0	0	0	0	0
>ath-miR172a MI0000215 Arabidopsis thaliana miR172a stem-loop												
AGAATCTTGATGATGCTGCAT	6746	74873	72944	9200	3520	15534	11667	2255	352	552	32	1981
>+1 ath-miR172a												
GAGAATCTTGATGATGCTGCAT	21	76	70	19	4	23	20	7	0	0	0	3
>+2 ath-miR172a												
TGAGAATCTTGATGATGCTGCAT	0	1	1	0	1	1	0	0	0	0	0	0
>+3 ath-miR172a												
ATGAGAATCTTGATGATGCTGCAT	0	0	0	0	0	0	0	0	0	0	0	0
>ath-MIR172b MI0000216 Arabidopsis thaliana miR172b stem-loop												

AGGCGCAGCACCATTAAGATTCACATGGA<br>AATTGATAAATACCCTAAATTAGGGTTTT<br>GATATGTATATGAGAATCTTGATGATGCT<br>GCATCAAC	0	0	0	0	0	0	0	0	0	0	0	0
>ath-miR172b MI0000216<br>Arabidopsis thaliana miR172b<br>stem-loop												
AGAATCTTGATGATGCTGCAT	6746	74873	72944	9200	3520	15534	11667	2255	352	552	32	1981
>+1 ath-miR172b												
GAGAATCTTGATGATGCTGCAT	21	76	70	19	4	23	20	7	0	0	0	3
>+2 ath-miR172b												
TGAGAATCTTGATGATGCTGCAT	0	1	1	0	1	1	0	0	0	0	0	0
>+3 ath-miR172b												
ATGAGAATCTTGATGATGCTGCAT	0	0	0	0	0	0	0	0	0	0	0	0
>ath-MIR172c MI0000991<br>Arabidopsis thaliana miR172c<br>stem-loop												
AGCTACTGTTCGCTGTTGGAGCATCATCA<br>AGATTCACAAATCATCAAGTATTCGTGTA<br>AATAAACCCATTTATGATTAGATTTTTGA<br>TGTATGTATGAGAATCTTGATGATGCTGC<br>AGCTGCAATCAGTGGCT	0	0	0	0	0	0	0	0	0	0	0	0
>ath-miR172c MI0000991<br>Arabidopsis thaliana miR172c<br>stem-loop												
AGAATCTTGATGATGCTGCAG	6746	74873	72944	9200	3520	15534	11667	2255	352	552	32	1981
>+1 ath-miR172c												
GAGAATCTTGATGATGCTGCAG	21	76	70	19	4	23	20	7	0	0	0	3
>+2 ath-miR172c												
TGAGAATCTTGATGATGCTGCAG	0	1	1	0	1	1	0	0	0	0	0	0
>+3 ath-miR172c												
ATGAGAATCTTGATGATGCTGCAG	0	0	0	0	0	0	0	0	0	0	0	0
>ath-MIR172d MI0000992<br>Arabidopsis thaliana miR172d<br>stem-loop												
AGTCATTGTTTGCTATTGCAACATCTTCA<br>AGATTCAGAAATCAGATTCTCTTATGGGT<br>TTTCTTTTGAGCCTTTATTTTTTGGTTTG<br>AGAATCTTGATGATGCTGCAGCGGCAATT<br>AAATGGCT	0	0	0	0	0	0	0	0	0	0	0	0
>ath-miR172d MI0000992<br>Arabidopsis thaliana miR172d<br>stem-loop												
AGAATCTTGATGATGCTGCAG	6746	74873	72944	9200	3520	15534	11667	2255	352	552	32	1981
>+1 ath-miR172d												
GAGAATCTTGATGATGCTGCAG	21	76	70	19	4	23	20	7	0	0	0	3
>+2 ath-miR172d												
TGAGAATCTTGATGATGCTGCAG	0	1	1	0	1	1	0	0	0	0	0	0
>+3 ath-miR172d												
TTGAGAATCTTGATGATGCTGCAG	0	0	0	0	0	0	1	0	0	0	0	0
>ath-MIR172e MI0001089<br>Arabidopsis thaliana miR172e<br>stem-loop												
GTAGTCGCAGATGCAGCACCATTAAGATT<br>CACAAGAGATGTGGTTCCCTTTGCTTTCG<br>CCTCTCGATCCGCAGAAAAGGGTTCCTTA<br>TCGAGTGGGAATCTTGATGATGCTGCATC<br>AGCAAATAC	0	0	0	0	0	0	0	0	0	0	0	0
>ath-miR172e MI0001089<br>Arabidopsis thaliana miR172e<br>stem-loop												
GGAATCTTGATGATGCTGCAT	27	296	274	275	87	32	15	44	3	23	1	68
>+1 ath-miR172e	3.7%	3.7%	2.2%	3.6%	4.6%	3.1%	13.3%	0.0%	0.0%	0.0%	0.0%	0.0%
GGGAATCTTGATGATGCTGCAT	1	11	6	10	4	1	2	0	0	0	0	0
>+2 ath-miR172e												
TGGGAATCTTGATGATGCTGCAT	0	0	1	0	1	0	0	0	0	0	0	0
>+3 ath-miR172e												
GTGGGAATCTTGATGATGCTGCAT	0	0	0	0	1	0	0	0	0	0	0	0
>ath-MIR173 MI0000217<br>Arabidopsis thaliana miR173<br>stem-loop												
TAAGTACTTTCGCTTGCAGAGAGAAATCA<br>CAGTGGTCAAAAAAGTTGTAGTTTTCTTA<br>AAGTCTCTTTCCTCTGTGATTCTCTGTGT<br>AAGCGAAAGAGCTTG	0	0	0	0	0	0	0	0	0	0	0	0
>ath-miR173 MI0000217<br>Arabidopsis thaliana miR173<br>stem-loop												
TTCGCTTGCAGAGAGAAATCAC	713	6141	6329	6777	4705	3430	3617	41	44	8	0	19
>+1 ath-miR173												
TTTCGCTTGCAGAGAGAAATCAC	0	0	0	0	0	0	0	3	0	0	0	2
>+2 ath-miR173												
CTTTCGCTTGCAGAGAGAAATCAC	0	0	0	0	0	0	0	0	0	0	0	0
>+3 ath-miR173												
ACTTTCGCTTGCAGAGAGAAATCAC	0	0	0	0	0	0	0	0	0	0	0	0
>ath-MIR1886 MI0008303<br>Arabidopsis thaliana miR1886<br>stem-loop												

GAGTGAGAGAAGTGAGATGAAATCTTTGA<br>TTGGAAATTTCATGTAGACGATGCTATCA<br>CCCCATATGACCAAAAGAAATTCCCAATT<br>AAAGATTTCATCTTACTTCCCTCATG	0	0	0	0	0	0	0	0	0	0	0	0
>ath-miR1886.1 MI0008303<br>Arabidopsis thaliana miR1886<br>stem-loop												
TGAGAGAAGTGAGATGAAATC	10	26	28	0	2	12	7	0	0	0	0	0
>+1 ath-miR1886.1												
GTGAGAGAAGTGAGATGAAATC	0	0	0	0	0	0	0	0	0	0	0	0
>+2 ath-miR1886.1												
AGTGAGAGAAGTGAGATGAAATC	0	0	0	0	0	0	0	0	0	0	0	0
>+3 ath-miR1886.1												
GAGTGAGAGAAGTGAGATGAAATC	0	0	0	0	0	0	0	0	0	0	0	0
>ath-MIR1886 MI0008303<br>Arabidopsis thaliana miR1886<br>stem-loop												
GAGTGAGAGAAGTGAGATGAAATCTTTGA<br>TTGGAAATTTCATGTAGACGATGCTATCA<br>CCCCATATGACCAAAAGAAATTCCCAATT<br>AAAGATTTCATCTTACTTCCCTCATG	0	0	0	0	0	0	0	0	0	0	0	0
>ath-miR1886.2 MI0008303<br>Arabidopsis thaliana miR1886<br>stem-loop												
TGAGATGAAATCTTTGATTGG	34	138	181	54	74	159	146	0	0	0	0	0
>+1 ath-miR1886.2												
GTGAGATGAAATCTTTGATTGG	0	0	0	0	0	0	0	0	0	0	0	0
>+2 ath-miR1886.2												
AGTGAGATGAAATCTTTGATTGG	0	0	0	0	0	0	0	0	0	0	0	0
>+3 ath-miR1886.2												
AAGTGAGATGAAATCTTTGATTGG	0	0	0	0	0	1	1	0	0	0	0	0
>ath-MIR1886 MI0008303<br>Arabidopsis thaliana miR1886<br>stem-loop												
GAGTGAGAGAAGTGAGATGAAATCTTTGA<br>TTGGAAATTTCATGTAGACGATGCTATCA<br>CCCCATATGACCAAAAGAAATTCCCAATT<br>AAAGATTTCATCTTACTTCCCTCATG	0	0	0	0	0	0	0	0	0	0	0	0
>ath-miR1886.3 MI0008303<br>Arabidopsis thaliana miR1886<br>stem-loop												
AATTAAAGATTTCATCTTACT	3	2	4	0	2	14	11	0	0	0	0	0
>+1 ath-miR1886.3												
CAATTAAAGATTTCATCTTACT	0	0	0	0	0	0	0	0	0	0	0	0
>+2 ath-miR1886.3												
CCAATTAAAGATTTCATCTTACT	0	0	0	0	0	0	0	0	0	0	0	0
>+3 ath-miR1886.3												
CCCAATTAAAGATTTCATCTTACT	0	0	0	0	0	0	0	0	0	0	0	0
>ath-MIR1887 MI0008304<br>Arabidopsis thaliana miR1887<br>stem-loop												
TTGTTCACTTAGATTGTTCTTAGTATGAG<br>GTGTTCTCGTACTAAGTAGAGTCTAAGAG<br>AACAA	0	0	0	0	0	0	0	0	0	0	0	0
>ath-miR1887 MI0008304<br>Arabidopsis thaliana miR1887<br>stem-loop												
TACTAAGTAGAGTCTAAGAGA	1	50	49	0	0	0	0	0	0	0	0	0
>+1 ath-miR1887												
GTACTAAGTAGAGTCTAAGAGA	0	1	0	0	0	0	0	0	0	0	0	0
>+2 ath-miR1887												
CGTACTAAGTAGAGTCTAAGAGA	2	29	29	0	0	0	0	0	0	0	0	0
>+3 ath-miR1887												
TCGTACTAAGTAGAGTCTAAGAGA	0	4	3	0	0	0	0	1	0	0	0	0
>ath-MIR1888 MI0008305<br>Arabidopsis thaliana miR1888<br>stem-loop												
CCAAATTTTTAAGTTAAGATTTGTGAAGA<br>AAGAGCTTTTATATAAGAGTTTTTAGGCT<br>TATATTTAAATTTTTTCTTCATAAATCTT<br>AGCTAAGATTTGG	0	0	0	0	0	0	0	0	0	0	0	0
>ath-miR1888 MI0008305<br>Arabidopsis thaliana miR1888<br>stem-loop												
TAAGTTAAGATTTGTGAAGAA	14	60	38	1	1	68	85	0	0	0	0	0
>+1 ath-miR1888												
TTAAGTTAAGATTTGTGAAGAA	6	23	32	0	0	36	35	0	0	0	0	0
>+2 ath-miR1888												
TTTAAGTTAAGATTTGTGAAGAA	0	0	0	0	0	0	0	0	0	0	0	0
>+3 ath-miR1888												
TTTTAAGTTAAGATTTGTGAAGAA	0	0	0	0	0	0	0	0	0	0	0	0
>ath-MIR2111a MI0010630<br>Arabidopsis thaliana miR2111a<br>stem-loop												

CGATGATGAGTATTGGTGAGGACCGGGTA<br>ATCTGCATCCTGAGGTTTAAAGCTTAATT<br>TACGCAGGAAATTTGTATACGCATATACG<br>TATGTGTATTAGTATACCTTTTAGTCCTC<br>GGGATGCGGATTACCTCGTTCTTACTTAC<br>AATACACGTACTCG	0	0	0	0	0	0	0	0	0	0	0	0
>ath-miR2111a MI0010630<br>Arabidopsis thaliana miR2111a<br>stem-loop												
TAATCTGCATCCTGAGGTTTA	3	18	14	1003	29	812	28	0	3	0	0	0
>+1 ath-miR2111a												
GTAATCTGCATCCTGAGGTTTA	0	0	0	0	0	0	0	0	0	0	0	0
>+2 ath-miR2111a												
GGTAATCTGCATCCTGAGGTTTA	0	0	0	0	0	0	0	0	0	0	0	0
>+3 ath-miR2111a												
GGGTAATCTGCATCCTGAGGTTTA	0	0	0	0	0	0	0	0	0	0	0	0
>ath-MIR2111b MI0010631<br>Arabidopsis thaliana miR2111b<br>stem-loop												
CAACAACACGAAGAAGAAGATGAAGGTAC<br>TTGATGATGAACGGGTAATCTGCATCCTG<br>AGGTTTAAAACCAATTAAGCATATACATA<br>TGCGAATGTATAGTTTTTAATCCTCGGGA<br>TACAGTTTACCTCTTCCTTACTCAAGAAA<br>ACACTTAGCTTTAACGGTTTTG	0	0	0	0	0	0	0	0	0	0	0	0
>ath-miR2111b MI0010631<br>Arabidopsis thaliana miR2111b<br>stem-loop												
TAATCTGCATCCTGAGGTTTA	3	18	14	1003	29	812	28	0	3	0	0	0
>+1 ath-miR2111b												
GTAATCTGCATCCTGAGGTTTA	0	0	0	0	0	0	0	0	0	0	0	0
>+2 ath-miR2111b												
GGTAATCTGCATCCTGAGGTTTA	0	0	0	0	0	0	0	0	0	0	0	0
>+3 ath-miR2111b												
GGGTAATCTGCATCCTGAGGTTTA	0	0	0	0	0	0	0	0	0	0	0	0
>ath-MIR2112 MI0010632<br>Arabidopsis thaliana miR2112<br>stem-loop												
GTATGAAATGATGCGCAAATGCGGATATC<br>AATGTAAATCAGGACAACAAACCAATAGA<br>GGTGCTCAGCGAAAACAACAAAATGTGAA<br>AAGGGTTTATCAGAAACATTGGGGTCATG<br>ATATACCTTTATATCCGCATTTGCGCATC<br>ATCTCTGAC	0	0	0	0	0	0	0	0	0	0	0	0
>ath-miR2112-3p MI0010632<br>Arabidopsis thaliana miR2112<br>stem-loop												
CTTTATATCCGCATTTGCGCA	3	3	0	19	26	15	12	0	0	0	0	0
>+1 ath-miR2112-3p	0.0%	0.0%	######	0.0%	3.8%	0.0%	0.0%	#####	#####	#####	#DIV/0!	#####
CCTTTATATCCGCATTTGCGCA	0	0	0	0	1	0	0	0	0	0	0	0
>+2 ath-miR2112-3p												
ACCTTTATATCCGCATTTGCGCA	0	0	0	0	0	0	0	0	0	0	0	0
>+3 ath-miR2112-3p												
TACCTTTATATCCGCATTTGCGCA	0	0	0	0	0	0	0	0	0	0	0	0
>ath-MIR2112 MI0010632<br>Arabidopsis thaliana miR2112<br>stem-loop												
GTATGAAATGATGCGCAAATGCGGATATC<br>AATGTAAATCAGGACAACAAACCAATAGA<br>GGTGCTCAGCGAAAACAACAAAATGTGAA<br>AAGGGTTTATCAGAAACATTGGGGTCATG<br>ATATACCTTTATATCCGCATTTGCGCATC<br>ATCTCTGAC	0	0	0	0	0	0	0	0	0	0	0	0
>ath-miR2112-5p MI0010632<br>Arabidopsis thaliana miR2112<br>stem-loop												
CGCAAATGCGGATATCAATGT	5	5	2	0	3	0	2	0	0	0	0	0
>+1 ath-miR2112-5p												
GCGCAAATGCGGATATCAATGT	0	0	0	0	0	0	0	0	0	0	0	0
>+2 ath-miR2112-5p												
TGCGCAAATGCGGATATCAATGT	0	0	0	0	0	0	0	0	0	0	0	0
>+3 ath-miR2112-5p												
ATGCGCAAATGCGGATATCAATGT	0	0	0	0	0	0	0	0	0	0	0	0
>ath-MIR319a MI0000544<br>Arabidopsis thaliana miR319a<br>stem-loop												
AGAGAGAGCTTCCTTGAGTCCATTCACAG<br>GTCGTGATATGATTCAATTAGCTTCCGAC<br>TCATTCATCCAAATACCGAGTCGCCAAAA<br>TTCAAACTAGACTCGTTAAATGAATGAAT<br>GATGCGGTAGACAAATTGGATCATTGATT<br>CTCTTTGATTGGACTGAAGGGAGCTCCCT<br>CT	0	0	0	0	0	0	0	0	0	0	0	0
>ath-miR319a MI0000544<br>Arabidopsis thaliana miR319a<br>stem-loop												
TTGGACTGAAGGGAGCTCCCT	100	128	130	8	3	41	66	7	3	1	0	9
>+1 ath-miR319a												

ATTGGACTGAAGGGAGCTCCCT	0	1	0	0	0	0	0	0	0	0	0	0
>+2 ath-miR319a												
GATTGGACTGAAGGGAGCTCCCT	0	0	0	0	0	0	0	0	0	0	0	0
>+3 ath-miR319a												
TGATTGGACTGAAGGGAGCTCCCT	0	0	0	0	0	0	0	0	0	0	0	0
>ath-MIR319b MI0000545 Arabidopsis thaliana miR319b stem-loop												
AGAGAGCTTTCTTCGGTCCACTCATGGAGTAATATGTGAGATTTAATTGACTCTCGACTCATTCATCCAAATACCAAATGAAAGAATTTGTTCTCATATGGTAAATGAATGAATGATGCGAGAGACAAATTGAGTCTTCACTTCTCTATGCTTGGACTGAAGGGAGCTCCCT	0	0	0	0	0	0	0	0	0	0	0	0
>ath-miR319b MI0000545 Arabidopsis thaliana miR319b stem-loop												
TTGGACTGAAGGGAGCTCCCT	100	128	130	8	3	41	66	7	3	1	0	9
>+1 ath-miR319b	0.0%	0.0%	2.3%	12.5%	0.0%	0.0%	3.0%	28.6%	0.0%	#####	#DIV/0!	0.0%
CTTGGACTGAAGGGAGCTCCCT	0	0	3	1	0	0	2	2	0	1	0	0
>+2 ath-miR319b												
GCTTGGACTGAAGGGAGCTCCCT	0	0	0	0	0	0	0	0	0	0	0	0
>+3 ath-miR319b												
TGCTTGGACTGAAGGGAGCTCCCT	0	0	0	0	0	0	0	0	0	0	0	0
>ath-MIR319c MI0001086 Arabidopsis thaliana miR319c stem-loop												
TAGATATAGAAGGAGATTCTTTCAGTCCAGTCATGGATAGAAAAAGAAGAGGGTAGAAATATCTGCCGACTCATCCATCCAAACACTCGTGGTAGAGAAACGATAAATTTAAACCGCAGTGACTGTGTGAATGATGCGGGAGATATTTTTGATCCTTCTTTATCTGTGTTTGGACTGAAGGGAGCTCCTTCTTTTTCTA	0	0	0	0	0	0	0	0	0	0	0	0
>ath-miR319c MI0001086 Arabidopsis thaliana miR319c stem-loop												
TTGGACTGAAGGGAGCTCCTT	0	3	3	0	2	2	1	10	5	6	0	5
>+1 ath-miR319c												
TTTGGACTGAAGGGAGCTCCTT	2	30	28	0	0	1	6	6	3	0	0	5
>+2 ath-miR319c												
GTTTGGACTGAAGGGAGCTCCTT	0	0	0	0	0	0	0	0	0	0	0	0
>+3 ath-miR319c												
TGTTTGGACTGAAGGGAGCTCCTT	0	0	0	0	0	0	0	0	0	0	0	0
>ath-MIR390a MI0001000 Arabidopsis thaliana miR390a stem-loop												
GTAGAGAAGAATCTGTAAAGCTCAGGAGGGATAGCGCCATGATGATCACATTCGTTATCTATTTTTTGGCGCTATCCATCCTGAGTTTCATTGGCTCTTCTTACTAC	0	0	0	0	0	0	0	0	0	0	0	0
>ath-miR390a MI0001000 Arabidopsis thaliana miR390a stem-loop												
AAGCTCAGGAGGGATAGCGCC	6110	37867	35382	1197	1336	8687	7094	142	6	24	1	67
>+1 ath-miR390a												
AAAGCTCAGGAGGGATAGCGCC	119	1153	1100	32	24	115	113	12	0	3	0	16
>+2 ath-miR390a												
TAAAGCTCAGGAGGGATAGCGCC	0	0	0	0	0	0	0	0	0	0	0	0
>+3 ath-miR390a												
GTAAAGCTCAGGAGGGATAGCGCC	0	0	0	0	0	0	0	0	0	0	0	0
>ath-MIR390b MI0001001 Arabidopsis thaliana miR390b stem-loop												
GAGAAATAGCTATAAAGCTCAGGAGGGATAGCGCCATGGCTCACCAGTGCTGTATGTTTTGTATATGCGTACATGTATATCTGTTGGCGCTATCCATCCTGAGTTCCATAGCTTCTTCTT	0	0	0	0	0	0	0	0	0	0	0	0
>ath-miR390b MI0001001 Arabidopsis thaliana miR390b stem-loop												
AAGCTCAGGAGGGATAGCGCC	6110	37867	35382	1197	1336	8687	7094	142	6	24	1	67
>+1 ath-miR390b												
AAAGCTCAGGAGGGATAGCGCC	119	1153	1100	32	24	115	113	12	0	3	0	16
>+2 ath-miR390b												
TAAAGCTCAGGAGGGATAGCGCC	0	0	0	0	0	0	0	0	0	0	0	0
>+3 ath-miR390b												
ATAAAGCTCAGGAGGGATAGCGCC	0	0	1	0	0	0	0	0	0	0	0	0
>ath-MIR391 MI0001002 Arabidopsis thaliana miR391 stem-loop												

TAGAGTGATTACACATACAAGTGGTCTTG<br>CTTCTTCTATGGTTTTAAAACTGCAAATA<br>AAGATTTGCTTCGCAGGAGAGATAGCGCC<br>ATCACCTCTTCTAAGAAGTTAACTAGTGG<br>TGACGGTATCTCTCCTACGTAGCAATCCT<br>TATATATGCATCTTAATACGGAGAGATCA<br>AGGTATTTCAGTTGTTCAATAATCACCCT<br>CTA	0	0	0	0	0	0	0	0	0	0	0	0
>ath-miR391 MI0001002<br>Arabidopsis thaliana miR391<br>stem-loop												
TTCGCAGGAGAGATAGCGCCA	1329	5341	5189	27	71	5744	2585	2	0	5	0	4
>+1 ath-miR391												
CTTCGCAGGAGAGATAGCGCCA	1	2	1	0	0	3	1	0	0	0	0	0
>+2 ath-miR391												
GCTTCGCAGGAGAGATAGCGCCA	0	0	0	0	0	0	0	0	0	0	0	0
>+3 ath-miR391												
TGCTTCGCAGGAGAGATAGCGCCA	0	0	0	0	0	0	0	0	0	0	0	0
>ath-MIR393a MI0001003<br>Arabidopsis thaliana miR393a<br>stem-loop												
AGAGGAAGGATCCAAAGGGATCGCATTGA<br>TCCTAATTAAGGTGAATTCTCCCCATATT<br>TTCTTTATAATTGGCAAATAAATCACAAA<br>AATTTGCTTGGTTTTGGATCATGCTATCT<br>CTTTGGATTCATCCTTC	0	0	0	0	0	0	0	0	0	0	0	0
>ath-miR393a MI0001003<br>Arabidopsis thaliana miR393a<br>stem-loop												
TCCAAAGGGATCGCATTGATCC	24	8	11	15	15	66	76	8	31	4	0	10
>+1 ath-miR393a												
ATCCAAAGGGATCGCATTGATCC	0	0	0	0	0	0	0	0	0	0	0	0
>+2 ath-miR393a												
GATCCAAAGGGATCGCATTGATCC	0	0	0	0	0	0	0	0	0	0	0	0
>+3 ath-miR393a												
GGATCCAAAGGGATCGCATTGATCC	0	0	0	0	0	0	0	0	0	0	0	0
>ath-MIR393b MI0001004<br>Arabidopsis thaliana miR393b<br>stem-loop												
AGAGAAAGGATCCAAAGGGATCGCATTGA<br>TCCTAATTAAGCTGATTTATTCCCCAATA<br>ATTGTTTTTTTTTTCCTTCTCAATCGAAA<br>GATGGAAGAAAAACAAATTCCAAACATTT<br>TGCTTACTTTTCCGGATCATGCGATCTCT<br>TTGGATTCATTCTTT	0	0	0	0	0	0	0	0	0	0	0	0
>ath-miR393b MI0001004<br>Arabidopsis thaliana miR393b<br>stem-loop												
TCCAAAGGGATCGCATTGATCC	24	8	11	15	15	66	76	8	31	4	0	10
>+1 ath-miR393b												
ATCCAAAGGGATCGCATTGATCC	0	0	0	0	0	0	0	0	0	0	0	0
>+2 ath-miR393b												
GATCCAAAGGGATCGCATTGATCC	0	0	0	0	0	0	0	0	0	0	0	0
>+3 ath-miR393b												
GGATCCAAAGGGATCGCATTGATCC	0	0	0	0	0	0	0	0	0	0	0	0
>ath-MIR394a MI0001005<br>Arabidopsis thaliana miR394a<br>stem-loop												
CTTACAGTCATCTTTGGCATTCTGTCCAC<br>CTCCTTCTATACATATATGCATGTGTATA<br>TATATATGCGTTTCGTGTGAAAGAAGGAG<br>GTGGGTATACTGCCAATAGAGATCTGTTA<br>G	0	0	0	0	0	0	0	0	0	0	0	0
>ath-miR394a MI0001005<br>Arabidopsis thaliana miR394a<br>stem-loop												
TTGGCATTCTGTCCACCTCC	247	647	639	114	98	459	439	3	3	1	1	4
>+1 ath-miR394a	4.5%	1.7%	3.0%	7.0%	4.1%	4.1%	3.0%	0.0%	0.0%	0.0%	0.0%	0.0%
TTTGGCATTCTGTCCACCTCC	11	11	19	8	4	19	13	0	0	0	0	0
>+2 ath-miR394a												
CTTTGGCATTCTGTCCACCTCC	0	0	0	0	0	0	0	0	0	0	0	0
>+3 ath-miR394a												
TCTTTGGCATTCTGTCCACCTCC	0	0	0	0	0	0	0	0	0	0	0	0
>ath-MIR394b MI0001006<br>Arabidopsis thaliana miR394b<br>stem-loop												
CTTACAGAGATCTTTGGCATTCTGTCCAC<br>CTCCTCTCTCTATATTTATGTGTAATAAG<br>TGTACGTATCTACGGTGTGTTTCGTAAGA<br>GGAGGTGGGCATACTGCCAATAGAGATCT<br>GTTAG	0	0	0	0	0	0	0	0	0	0	0	0
>ath-miR394b MI0001006<br>Arabidopsis thaliana miR394b<br>stem-loop												
TTGGCATTCTGTCCACCTCC	247	647	639	114	98	459	439	3	3	1	1	4
>+1 ath-miR394b	4.5%	1.7%	3.0%	7.0%	4.1%	4.1%	3.0%	0.0%	0.0%	0.0%	0.0%	0.0%
TTTGGCATTCTGTCCACCTCC	11	11	19	8	4	19	13	0	0	0	0	0
>+2 ath-miR394b												

CTTTGGCATTCTGTCCACCTCC	0	0	0	0	0	0	0	0	0	0	0	0
>+3 ath-miR394b												
TCTTTGGCATTCTGTCCACCTCC	0	0	0	0	0	0	0	0	0	0	0	0
>ath-MIR395a MI0001007<br>Arabidopsis thaliana miR395a<br>stem-loop												
ATGTCTCCTAGAGTTCCTCTGAGCACTTC<br>ATTGGGGATACAATTTTTCTAAATGATTA<br>TCCACTGAAGTGTTTGGGGGAACTCCCGG<br>ACCCAT	0	0	0	0	0	0	0	0	0	0	0	0
>ath-miR395a MI0001007<br>Arabidopsis thaliana miR395a<br>stem-loop												
CTGAAGTGTTTGGGGGAACTC	3	11	8	4	15	10	32	0	0	1	0	0
>+1 ath-miR395a												
ACTGAAGTGTTTGGGGGAACTC	0	0	0	0	0	0	0	0	0	0	0	0
>+2 ath-miR395a												
CACTGAAGTGTTTGGGGGAACTC	0	0	0	0	0	0	0	0	0	0	0	0
>+3 ath-miR395a												
CCACTGAAGTGTTTGGGGGAACTC	0	0	0	0	0	0	1	0	0	0	0	0
>ath-MIR395b MI0001008<br>Arabidopsis thaliana miR395b<br>stem-loop												
ATGTCCCCATGAGTTCCCTTTAACGCTTC<br>ATTGTTAAATACTCAAAGCCACATTGGTT<br>TGTATACAACACTGAAGTGTTTGGGGGGA<br>CTCTTGGTGTCAT	0	0	0	0	0	0	0	0	0	0	0	0
>ath-miR395b MI0001008<br>Arabidopsis thaliana miR395b<br>stem-loop												
CTGAAGTGTTTGGGGGGACTC	8	28	19	3	11	3	43	0	0	0	0	0
>+1 ath-miR395b												
ACTGAAGTGTTTGGGGGGACTC	0	0	0	0	0	0	0	0	0	0	0	0
>+2 ath-miR395b												
CACTGAAGTGTTTGGGGGGACTC	0	0	0	0	0	0	0	0	0	0	0	0
>+3 ath-miR395b												
ACACTGAAGTGTTTGGGGGGACTC	0	0	0	0	0	0	0	0	0	0	0	0
>ath-MIR395c MI0001009<br>Arabidopsis thaliana miR395c<br>stem-loop												
ATGTCCACATGAGTTCCCTTTAACGCTTC<br>ATTGTTGAATACTCAAAGCCACATTGGTT<br>TGTATATAACACTGAAGTGTTTGGGGGGA<br>CTCTTGGTGTCAT	0	0	0	0	0	0	0	0	0	0	0	0
>ath-miR395c MI0001009<br>Arabidopsis thaliana miR395c<br>stem-loop												
CTGAAGTGTTTGGGGGGACTC	8	28	19	3	11	3	43	0	0	0	0	0
>+1 ath-miR395c												
ACTGAAGTGTTTGGGGGGACTC	0	0	0	0	0	0	0	0	0	0	0	0
>+2 ath-miR395c												
CACTGAAGTGTTTGGGGGGACTC	0	0	0	0	0	0	0	0	0	0	0	0
>+3 ath-miR395c												
ACACTGAAGTGTTTGGGGGGACTC	0	0	0	0	0	0	0	0	0	0	0	0
>ath-MIR395d MI0001010<br>Arabidopsis thaliana miR395d<br>stem-loop												
ATGTCCTCTAGAGTTCTCCTGAACACTTC<br>ATTGGAAATTTGTTATTCAGTAAGCTAAC<br>AGTTAATTCCACTGAAGTGTTTGGGGGAA<br>CTCCCGATGTCAT	0	0	0	0	0	0	0	0	0	0	0	0
>ath-miR395d MI0001010<br>Arabidopsis thaliana miR395d<br>stem-loop												
CTGAAGTGTTTGGGGGAACTC	3	11	8	4	15	10	32	0	0	1	0	0
>+1 ath-miR395d												
ACTGAAGTGTTTGGGGGAACTC	0	0	0	0	0	0	0	0	0	0	0	0
>+2 ath-miR395d												
CACTGAAGTGTTTGGGGGAACTC	0	0	0	0	0	0	0	0	0	0	0	0
>+3 ath-miR395d												
CCACTGAAGTGTTTGGGGGAACTC	0	0	0	0	0	0	1	0	0	0	0	0
>ath-MIR395e MI0001011<br>Arabidopsis thaliana miR395e<br>stem-loop												
ATGTTTTCTAGAGTTCCTCTGAGCACTTC<br>ATTGGAGATACAATTTTTTATAAAATAGT<br>TTTCTACTGAAGTGTTTGGGGGAACTCCC<br>GGGCTGAT	0	0	0	0	0	0	0	0	0	0	0	0
>ath-miR395e MI0001011<br>Arabidopsis thaliana miR395e<br>stem-loop												
CTGAAGTGTTTGGGGGAACTC	3	11	8	4	15	10	32	0	0	1	0	0
>+1 ath-miR395e												
ACTGAAGTGTTTGGGGGAACTC	0	0	0	0	0	0	0	0	0	0	0	0
>+2 ath-miR395e												
TACTGAAGTGTTTGGGGGAACTC	0	0	0	0	0	0	0	0	0	0	0	0
>+3 ath-miR395e												
CTACTGAAGTGTTTGGGGGAACTC	0	0	0	0	0	0	0	0	0	0	0	0

>ath-MIR395f MI0001012<br>Arabidopsis thaliana miR395f<br>stem-loop												
ATGTCCCCTTGAGTTCCCTTAAACGCTTC<br>ATTGTTCATACTTTGTTATCATCTATCGA<br>TCGATCAATCAATCTGATGAACACTGAAG<br>TGTTTGGGGGGACTCTAGGTGACAT	0	0	0	0	0	0	0	0	0	0	0	0
>ath-miR395f MI0001012<br>Arabidopsis thaliana miR395f<br>stem-loop												
CTGAAGTGTTTGGGGGGACTC	8	28	19	3	11	3	43	0	0	0	0	0
>+1 ath-miR395f												
ACTGAAGTGTTTGGGGGGACTC	0	0	0	0	0	0	0	0	0	0	0	0
>+2 ath-miR395f												
CACTGAAGTGTTTGGGGGGACTC	0	0	0	0	0	0	0	0	0	0	0	0
>+3 ath-miR395f												
ACACTGAAGTGTTTGGGGGGACTC	0	0	0	0	0	0	0	0	0	0	0	0
>ath-MIR396a MI0001013<br>Arabidopsis thaliana miR396a<br>stem-loop												
CTCTGTATTCTTCCACAGCTTTCTTGAAC<br>TGCAAAACTTCTTCAGATTTTTTTTTTT<br>TCTTTTGATATCTCTTACGCATAAAATAG<br>TGATTTTCTTCATATCTCTGCTCGATTGA<br>TTTGCGGTTCAATAAAGCTGTGGGAAGAT<br>ACAGAC	0	0	0	0	0	0	0	0	0	0	0	0
>ath-miR396a MI0001013<br>Arabidopsis thaliana miR396a<br>stem-loop												
TTCCACAGCTTTCTTGAACTG	228	1021	972	2147	1807	4961	4496	7	34	8	4	53
>+1 ath-miR396a												
CTTCCACAGCTTTCTTGAACTG	0	0	1	0	0	2	1	0	0	0	0	0
>+2 ath-miR396a												
TCTTCCACAGCTTTCTTGAACTG	0	0	0	0	0	0	0	0	0	0	0	0
>+3 ath-miR396a												
TTCTTCCACAGCTTTCTTGAACTG	0	0	0	0	0	0	0	0	0	0	0	0
>ath-MIR396b MI0001014<br>Arabidopsis thaliana miR396b<br>stem-loop												
GGTCATACTTTTCCACAGCTTTCTTGAAC<br>TTTCTTTTTCATTTCCATTGTTTTTTTCT<br>TAAACAAAAGTAAGAAGAAAAAAAACTTT<br>AAGATTAAGCATTTTGGAAGCTCAAGAAA<br>GCTGTGGGAAAACATGACA	0	0	0	0	0	0	0	0	0	0	0	0
>ath-miR396b MI0001014<br>Arabidopsis thaliana miR396b<br>stem-loop												
TTCCACAGCTTTCTTGAACTT	228	1021	972	2147	1807	4961	4496	7	34	8	4	53
>+1 ath-miR396b												
TTTCCACAGCTTTCTTGAACTT	0	0	1	0	0	0	1	0	0	0	0	0
>+2 ath-miR396b												
TTTTCCACAGCTTTCTTGAACTT	0	0	0	0	0	0	0	0	0	0	0	0
>+3 ath-miR396b												
CTTTTCCACAGCTTTCTTGAACTT	0	0	0	0	0	0	0	0	0	0	0	0
>ath-MIR397a MI0001015<br>Arabidopsis thaliana miR397a<br>stem-loop												
TGAATGAACATCATTGAGTGCAGCGTTGA<br>TGTAATTTCGTTTTGTTTTTCATTGTTGA<br>ATGGATTAAAAGAATTTATACCAGCGTTG<br>CGCTCAATTATGTTTTTCTA	0	0	0	0	0	0	0	0	0	0	0	0
>ath-miR397a MI0001015<br>Arabidopsis thaliana miR397a<br>stem-loop												
TCATTGAGTGCAGCGTTGATG	0	165	162	0	1	3	6	0	0	0	0	0
>+1 ath-miR397a												
ATCATTGAGTGCAGCGTTGATG	0	0	0	0	0	0	0	0	0	0	0	0
>+2 ath-miR397a												
CATCATTGAGTGCAGCGTTGATG	0	0	0	0	0	0	0	0	0	0	0	0
>+3 ath-miR397a												
ACATCATTGAGTGCAGCGTTGATG	0	0	0	0	0	0	0	0	0	0	0	0
>ath-MIR397b MI0001016<br>Arabidopsis thaliana miR397b<br>stem-loop												
TGAATGAACATCATTGAGTGCATCGTTGA<br>TGTAATTTTACTTATTTTATTCCATTGTT<br>GAATTAATTAAAGAAGTATATATCAGCGT<br>TGCATTCAATTATGTTTTTCTA	0	0	0	0	0	0	0	0	0	0	0	0
>ath-miR397b MI0001016<br>Arabidopsis thaliana miR397b<br>stem-loop												
TCATTGAGTGCATCGTTGATG	5	104	99	7	13	1	8	0	0	0	0	1
>+1 ath-miR397b												
ATCATTGAGTGCATCGTTGATG	0	0	1	0	0	0	0	0	0	0	0	0
>+2 ath-miR397b												
CATCATTGAGTGCATCGTTGATG	0	0	0	0	0	0	0	0	0	0	0	0
>+3 ath-miR397b												
ACATCATTGAGTGCATCGTTGATG	0	0	0	0	0	0	0	0	0	0	0	0

>ath-MIR398a MI0001017 Arabidopsis thaliana miR398a stem-loop												
TGAAATTTCAAAGGAGTGGCATGTGAACACATATCCTATGGTTTCTTCAAATTTCCATTGAAACCATTGAGTTTTGTGTTCTCAGGTCACCCCTTTGAATCTCCC	0	0	0	0	0	0	0	0	0	0	0	0
>ath-miR398a MI0001017 Arabidopsis thaliana miR398a stem-loop												
TGTGTTCTCAGGTCACCCCTT	0	4	5	1	7	9	32	2	17	1	0	35
>+1 ath-miR398a												
TTGTGTTCTCAGGTCACCCCTT	0	0	0	0	0	0	0	0	0	0	0	0
>+2 ath-miR398a												
TTTGTGTTCTCAGGTCACCCCTT	0	0	0	0	0	0	0	0	0	0	0	0
>+3 ath-miR398a												
TTTTGTGTTCTCAGGTCACCCCTT	0	0	0	0	0	0	0	0	0	0	0	0
>ath-MIR398b MI0001018 Arabidopsis thaliana miR398b stem-loop												
TGGATCTCGACAGGGTTGATATGAGAACACACGAGTAATCAACGGCTGTAATGACGCTACGTCATTGTTACAGCTCTCGTTTTCATGTGTTCTCAGGTCACCCCTGCTGAGCTCTT	0	0	0	0	0	0	0	0	0	0	0	0
>ath-miR398b MI0001018 Arabidopsis thaliana miR398b stem-loop												
TGTGTTCTCAGGTCACCCCTG	0	4	5	1	7	9	32	2	17	1	0	35
>+1 ath-miR398b												
ATGTGTTCTCAGGTCACCCCTG	0	0	1	0	1	0	2	0	0	0	0	0
>+2 ath-miR398b												
CATGTGTTCTCAGGTCACCCCTG	0	0	0	0	0	0	0	0	0	0	0	0
>+3 ath-miR398b												
TCATGTGTTCTCAGGTCACCCCTG	0	0	0	0	0	1	0	0	0	0	0	0
>ath-MIR398c MI0001019 Arabidopsis thaliana miR398c stem-loop												
TGGATCTCGACAGGGTTGATATGAGAACACACGAGCAATCAACGGCTATAACGACGCTACGTCATTGTTACAGCTCTCGTTTCATGTGTTCTCAGGTCACCCCTGCTGAGCTCTT	0	0	0	0	0	0	0	0	0	0	0	0
>ath-miR398c MI0001019 Arabidopsis thaliana miR398c stem-loop												
TGTGTTCTCAGGTCACCCCTG	0	4	5	1	7	9	32	2	17	1	0	35
>+1 ath-miR398c												
ATGTGTTCTCAGGTCACCCCTG	0	0	1	0	1	0	2	0	0	0	0	0
>+2 ath-miR398c												
CATGTGTTCTCAGGTCACCCCTG	0	0	0	0	0	0	0	0	0	0	0	0
>+3 ath-miR398c												
TCATGTGTTCTCAGGTCACCCCTG	0	0	0	0	0	1	0	0	0	0	0	0
>ath-MIR399a MI0001020 Arabidopsis thaliana miR399a stem-loop												
AAATGCATTACAGGGTAAGATCTCTATTGGCAGGAAACCATTACTTAGATCTTTGCATCTCTTTATGCATTGCTTTTAATTAGTGAGTTATCTGCCAAAGGAGATTTGCCCTGTAATTCTTCT	0	0	0	0	0	0	0	0	0	0	0	0
>ath-miR399a MI0001020 Arabidopsis thaliana miR399a stem-loop												
TGCCAAAGGAGATTTGCCCTG	0	1	2	182	6	205	6	0	1	0	0	0
>+1 ath-miR399a	#####	0.0%	0.0%	3.3%	0.0%	1.0%	0.0%	#####	0.0%	#####	#DIV/0!	#####
CTGCCAAAGGAGATTTGCCCTG	0	0	0	6	0	2	0	0	0	0	0	0
>+2 ath-miR399a												
TCTGCCAAAGGAGATTTGCCCTG	0	0	0	9	0	3	0	0	0	0	0	0
>+3 ath-miR399a												
ATCTGCCAAAGGAGATTTGCCCTG	0	0	0	8	0	1	0	0	0	0	0	0
>ath-MIR399b MI0001021 Arabidopsis thaliana miR399b stem-loop												
TCACTAGTTTTAGGGCGCCTCTCCATTGGCAGGTCCTTTACTTCCAAATATACACATACATATATGAATATCGAAAATTTCCGATGATCGATTTATAAATGACCTGCCAAAGGAGAGTTGCCCTGAAACTGGTTC	0	0	0	0	0	0	0	0	0	0	0	0
>ath-miR399b MI0001021 Arabidopsis thaliana miR399b stem-loop												
TGCCAAAGGAGAGTTGCCCTG	38	113	70	962	16	1238	39	0	3	0	0	1
>+1 ath-miR399b												
CTGCCAAAGGAGAGTTGCCCTG	0	0	0	4	0	0	0	0	0	0	0	0
>+2 ath-miR399b												
CCTGCCAAAGGAGAGTTGCCCTG	0	0	0	0	0	0	0	0	0	0	0	0
>+3 ath-miR399b												

ACCTGCCAAAGGAGAGTTGCCCTG	0	0	0	1	0	0	0	0	0	0	0	0
>ath-MIR399c MI0001022 Arabidopsis thaliana miR399c stem-loop												
GGAGCAGTAATAGGGCATCTTTCTATTGGCAGGCGACTTGGCTATTTGTATCTTTTGTGTTCTTGACTATTGGCTATGTCACTTGCCAAAGGAGAGTTGCCCTGTCACTGCTTC	0	0	0	0	0	0	0	0	0	0	0	0
>ath-miR399c MI0001022 Arabidopsis thaliana miR399c stem-loop												
TGCCAAAGGAGAGTTGCCCTG	38	113	70	962	16	1238	39	0	3	0	0	1
>+1 ath-miR399c	0.0%	0.9%	2.9%	0.8%	0.0%	0.9%	7.7%	#####	0.0%	#####	#DIV/0!	0.0%
TTGCCAAAGGAGAGTTGCCCTG	0	1	2	8	0	11	3	0	0	0	0	0
>+2 ath-miR399c												
CTTGCCAAAGGAGAGTTGCCCTG	0	0	0	0	1	0	0	0	0	0	0	0
>+3 ath-miR399c												
ACTTGCCAAAGGAGAGTTGCCCTG	0	0	0	9	0	1	0	0	0	0	0	0
>ath-MIR399d MI0001023 Arabidopsis thaliana miR399d stem-loop												
GGTTGGATTACTGGGCGAATACTCCTATGGCAGATCGCATTGGCTAGATATGCAAGTAAAATGCTTCTCTGCCAAAGGAGATTTGCCCCGCAATTCATCC	0	0	0	0	0	0	0	0	0	0	0	0
>ath-miR399d MI0001023 Arabidopsis thaliana miR399d stem-loop												
TGCCAAAGGAGATTTGCCCCG	0	0	0	636	0	217	0	0	0	0	0	0
>+1 ath-miR399d												
CTGCCAAAGGAGATTTGCCCCG	0	0	0	3	0	1	0	0	0	0	0	0
>+2 ath-miR399d												
TCTGCCAAAGGAGATTTGCCCCG	0	0	0	0	0	0	0	0	0	0	0	0
>+3 ath-miR399d												
CTCTGCCAAAGGAGATTTGCCCCG	0	0	0	0	0	0	0	0	0	0	0	0
>ath-MIR399e MI0001024 Arabidopsis thaliana miR399e stem-loop												
GAAAGCATTACAGGGCGAATCCTCTATTGGCAGTGGAAGTTGATGACCCTTATATGTTATTTTCTCATCATTTTCCTCTGCCAAAGGAGATTTGCCTCGCAATGCTTCA	0	0	0	0	0	0	0	0	0	0	0	0
>ath-miR399e MI0001024 Arabidopsis thaliana miR399e stem-loop												
TGCCAAAGGAGATTTGCCTCG	0	0	0	3	0	0	0	0	0	0	0	0
>+1 ath-miR399e												
CTGCCAAAGGAGATTTGCCTCG	0	0	0	0	0	0	0	0	0	0	0	0
>+2 ath-miR399e												
TCTGCCAAAGGAGATTTGCCTCG	0	0	0	0	0	0	0	0	0	0	0	0
>+3 ath-miR399e												
CTCTGCCAAAGGAGATTTGCCTCG	0	0	0	0	0	0	0	0	0	0	0	0
>ath-MIR399f MI0001025 Arabidopsis thaliana miR399f stem-loop												
ATATGCATTACAGGGCAAGATCACCATTGGCAGAGATCTATTACTTCATTCTTGCATCATATGCATAAATGTTTGTGGTGAGCTCTCTGCCAAAGGAGATTTGCCCGGTAATTCTCTT	0	0	0	0	0	0	0	0	0	0	0	0
>ath-miR399f MI0001025 Arabidopsis thaliana miR399f stem-loop												
TGCCAAAGGAGATTTGCCCGG	4	1	3	5630	7	566	5	0	3	0	0	0
>+1 ath-miR399f	0.0%	0.0%	0.0%	1.6%	0.0%	1.6%	0.0%	#####	33.3%	#####	#DIV/0!	#####
CTGCCAAAGGAGATTTGCCCGG	0	0	0	92	0	9	0	0	1	0	0	0
>+2 ath-miR399f												
TCTGCCAAAGGAGATTTGCCCGG	0	0	0	23	0	3	0	0	0	0	0	0
>+3 ath-miR399f												
CTCTGCCAAAGGAGATTTGCCCGG	0	0	0	7	0	1	0	0	0	0	0	0
>ath-MIR400 MI0001069 Arabidopsis thaliana miR400 stem-loop												
TGAGGATTGTTTATGAGAGTATTATAAGTCACTACATTTGGTAAGCAAAGTGTTGTTTCTCAAACGAAGTGACTTATGATAATCTCATGAATGGATTTTGCA	0	0	0	0	0	0	0	0	0	0	0	0
>ath-miR400 MI0001069 Arabidopsis thaliana miR400 stem-loop												
TATGAGAGTATTATAAGTCAC	10	6	7	60	52	192	215	0	17	0	1	0
>+1 ath-miR400												
TTATGAGAGTATTATAAGTCAC	0	0	0	0	2	0	2	0	0	0	0	0
>+2 ath-miR400												
TTTATGAGAGTATTATAAGTCAC	0	0	0	0	0	0	0	0	0	0	0	0
>+3 ath-miR400												
GTTTATGAGAGTATTATAAGTCAC	0	0	0	0	0	0	0	0	0	0	0	0

>ath-MIR401 MI0001070 Arabidopsis thaliana miR401 stem-loop												
TATATAGACCCTAAAAACGTCCAGGGACTAATAATGCAAATATGGAAATCTTCTGGGGCAAACTTGAGCTTCTGAAAACTTGAAAGCGTTTAGGGTTTTTGCTGGGCCGAAACTGGTGTCGACCGACACCAAGAGTGTGTCGCTCGACACTCATCGCTGATTCCTGAACCAAAATCGTTCTTAGTTTCCTTTTCCTTAGTTTTTGCTCCAAAATGTCTCCTTATCTCCATTGTTGTCCCATTGCATAGAA	0	0	0	0	0	0	0	0	0	0	0	0
>ath-miR401 MI0001070 Arabidopsis thaliana miR401 stem-loop												
CGAAACTGGTGTCGACCGACA	0	0	0	0	0	0	0	0	0	0	0	0
>+1 ath-miR401												
CCGAAACTGGTGTCGACCGACA	0	0	0	0	0	0	0	0	0	0	0	0
>+2 ath-miR401												
GCCGAAACTGGTGTCGACCGACA	0	0	0	0	0	0	0	0	0	0	0	0
>+3 ath-miR401												
GGCCGAAACTGGTGTCGACCGACA	0	0	0	0	0	0	0	0	0	0	0	0
>ath-MIR402 MI0001071 Arabidopsis thaliana miR402 stem-loop												
CGTGGTAGATAAGTTTGAGTTGCATAGTGGCAGTCTTCCTTTGTTTGTTAACCCATATTTTCATGATTCGAGGCCTATTAAACCTCTGTTACATCTGCTTTTTGAAAAGTTGTCATTTTTCTGAAATCTTCTTGCCTCAATTTCCAACAGCAGATTCATCTTTCATTGGATGATCCGTTAGTTTTTGATAGAAAAAATGAGGTGGGAAGAAATTAAGACTGACTTCGAAATAGCCTTTAAAATAGGGGTTTATAGACCTCGAATCATATCAAACGAGTCTGCTACTATGCTACTGAAAACTTTATCAAA	0	0	0	0	0	0	0	0	0	0	0	0
>ath-miR402 MI0001071 Arabidopsis thaliana miR402 stem-loop												
TTCGAGGCCTATTAAACCTCTG	16	5	4	2	14	17	28	0	0	0	0	0
>+1 ath-miR402												
ATTCGAGGCCTATTAAACCTCTG	1	0	0	0	0	0	1	0	0	0	0	0
>+2 ath-miR402												
GATTCGAGGCCTATTAAACCTCTG	0	0	0	0	0	0	0	0	0	0	0	0
>+3 ath-miR402												
TGATTCGAGGCCTATTAAACCTCTG	0	0	0	0	0	0	0	0	0	0	0	0
>ath-MIR403 MI0001072 Arabidopsis thaliana miR403 stem-loop												
TTGTCATTAGAAGAGTCGTATTACATGTTTTGTGCTTGAATCTAATTCAACAGGCTTTATGTAAGAGATTCTTTAACAATTCCTATAATCTTTGTTGTTGGATTAGATTCACGCACAAACTCGTAATCTGTCTTT	0	0	0	0	0	0	0	0	0	0	0	0
>ath-miR403 MI0001072 Arabidopsis thaliana miR403 stem-loop												
TTAGATTCACGCACAAACTCG	316	1192	1191	1541	1200	1609	1240	2	4	2	0	2
>+1 ath-miR403												
ATTAGATTCACGCACAAACTCG	0	0	0	0	1	0	1	0	0	0	0	0
>+2 ath-miR403												
GATTAGATTCACGCACAAACTCG	0	0	0	0	0	0	0	0	0	0	0	0
>+3 ath-miR403												
GGATTAGATTCACGCACAAACTCG	4	5	5	9	7	11	7	0	0	0	0	0
>ath-MIR404 MI0001073 Arabidopsis thaliana miR404 stem-loop												
TAGCATGTTCGTTTCATTAACGCTGGCGGTTGCGGCAGCGGCTGCGGTAGCGGTGGCGGCAAACACTACCGCAGGTTGTTGTTCGTTTTGTTGCCGCAGACGCTGCCGCAGCCGCTGCCGCAACCGCAGGTTTCTTTGTTCGTTTCGACG	0	0	0	0	0	0	0	0	0	0	0	0
>ath-miR404 MI0001073 Arabidopsis thaliana miR404 stem-loop												
ATTAACGCTGGCGGTTGCGGCAGC	0	0	0	0	0	0	0	0	0	0	0	0
>+1 ath-miR404												
CATTAACGCTGGCGGTTGCGGCAGC	0	0	0	0	0	0	0	0	0	0	0	0
>+2 ath-miR404												
TCATTAACGCTGGCGGTTGCGGCAGC	0	0	0	0	0	0	0	0	0	0	0	0
>+3 ath-miR404												
TTCATTAACGCTGGCGGTTGCGGCAGC	0	0	0	0	0	0	0	0	0	0	0	0
>ath-MIR405a MI0001074 Arabidopsis thaliana miR405a stem-loop												

TCAAAATGGGTAACCCAACCCAACCCAAC<br>TCATAATCAAATGAGTTTATGATTAAATG<br>AGTTATGGGTTGACCCAACTCATTTTGTT<br>AAATGAGTTGGGTCTAACCCATAACTCAT<br>TTCATTTGATGGGTTGAGTTGTTAAATGG<br>GTTAACCATTTA	0	0	0	0	0	0	0	0	0	0	0	0
>ath-miR405a MI0001074<br>Arabidopsis thaliana miR405a<br>stem-loop												
ATGAGTTGGGTCTAACCCATAACT	0	0	0	0	0	0	0	0	0	0	0	0
>+1 ath-miR405a												
AATGAGTTGGGTCTAACCCATAACT	0	0	0	0	0	0	0	0	0	0	0	0
>+2 ath-miR405a												
AAATGAGTTGGGTCTAACCCATAACT	0	0	0	0	0	0	0	0	0	0	0	0
>+3 ath-miR405a												
TAAATGAGTTGGGTCTAACCCATAACT	0	0	0	0	0	0	0	0	0	0	0	0
>ath-MIR405b MI0001075<br>Arabidopsis thaliana miR405b<br>stem-loop												
TTAACCCATTTAACAATTCAACCCATCAA<br>ATGAAATGAGTTATGGGTTAGACCCAACT<br>CATTTAACAAAATGAGTTGGGTCTAACCC<br>ATAACTCATTTAATTATAAACTCATTTGA<br>TTATGAGT	0	0	0	0	0	0	0	0	0	0	0	0
>ath-miR405b MI0001075<br>Arabidopsis thaliana miR405b<br>stem-loop												
ATGAGTTGGGTCTAACCCATAACT	0	0	0	0	0	0	0	0	0	0	0	0
>+1 ath-miR405b												
AATGAGTTGGGTCTAACCCATAACT	0	0	0	0	0	0	0	0	0	0	0	0
>+2 ath-miR405b												
AAATGAGTTGGGTCTAACCCATAACT	0	0	0	0	0	0	0	0	0	0	0	0
>+3 ath-miR405b												
AAAATGAGTTGGGTCTAACCCATAACT	0	0	0	0	0	0	0	0	0	0	0	0
>ath-MIR405d MI0001077<br>Arabidopsis thaliana miR405d<br>stem-loop												
ACCCATCAAATGAAATGAGTTATGGGTTG<br>ACCCAACTCATTTTGTTAAATGAGTTGGG<br>TCTAACCCATAACTCATTTAATCATAAA	0	0	0	0	0	0	0	0	0	0	0	0
>ath-miR405d MI0001077<br>Arabidopsis thaliana miR405d<br>stem-loop												
ATGAGTTGGGTCTAACCCATAACT	0	0	0	0	0	0	0	0	0	0	0	0
>+1 ath-miR405d												
AATGAGTTGGGTCTAACCCATAACT	0	0	0	0	0	0	0	0	0	0	0	0
>+2 ath-miR405d												
AAATGAGTTGGGTCTAACCCATAACT	0	0	0	0	0	0	0	0	0	0	0	0
>+3 ath-miR405d												
TAAATGAGTTGGGTCTAACCCATAACT	0	0	0	0	0	0	0	0	0	0	0	0
>ath-MIR406 MI0001078<br>Arabidopsis thaliana miR406<br>stem-loop												
TAAAGTCTTAATTTTTAGTTATCACGATT<br>TAGCACATATCAATCTATAGATTTGATTT<br>TTCTTTTATTAGTTCTTAAATTTTGATTT<br>GAGACAGTAACTATTCAAATAGAATGCTA<br>TTGTAATCCAGAATCCGAAAGAAACGAAT<br>CAAAAAAAAAAAAAACGAGTCTTCATAGT<br>AAAAATCGATTAACTAAAAAGGATGG	0	0	0	0	0	0	0	0	0	0	0	0
>ath-miR406 MI0001078<br>Arabidopsis thaliana miR406<br>stem-loop												
TAGAATGCTATTGTAATCCAG	0	0	0	0	0	0	0	0	0	0	0	0
>+1 ath-miR406												
ATAGAATGCTATTGTAATCCAG	0	0	0	0	0	1	0	0	0	0	0	0
>+2 ath-miR406												
AATAGAATGCTATTGTAATCCAG	0	0	0	1	1	2	2	0	0	0	0	0
>+3 ath-miR406												
AAATAGAATGCTATTGTAATCCAG	0	0	1	0	0	0	3	0	0	0	0	0
>ath-MIR407 MI0001079<br>Arabidopsis thaliana miR407<br>stem-loop												
TGGGAAAAATGTTAAAAAAATCGCCAACT<br>TTTAAAAATGGGACAAAAAAATCGCCAAC<br>TCCTGAAATGTCATTTAAATCATATACTT<br>TTGGTTGACTTTTCCAGAAAGATAATAAA<br>GCAAAGATTCGTAATCAACAAAACTTTGC<br>TCTGTTATCTTTCTAAAGAACTCAATCAA<br>AAGTATGTGATTAAAACGACATTTCAGGA<br>GTTGGTGATTTTTCATCCCATTTTTCAAA<br>GTTGGCAATTTTTTTGACATTTTTTCCAA<br>TG	0	0	0	0	0	0	0	0	0	0	0	0
>ath-miR407 MI0001079<br>Arabidopsis thaliana miR407<br>stem-loop												

TTTAAATCATATACTTTTGGT	0	0	0	0	0	0	0	0	0	0	0	0
>+1 ath-miR407												
ATTTAAATCATATACTTTTGGT	0	0	0	0	0	0	0	0	0	0	0	0
>+2 ath-miR407												
CATTTAAATCATATACTTTTGGT	0	0	0	0	0	0	0	0	0	0	0	0
>+3 ath-miR407												
TCATTTAAATCATATACTTTTGGT	0	0	0	0	0	0	0	0	0	0	0	0
>ath-MIR408 MI0001080<br>Arabidopsis thaliana miR408<br>stem-loop												
AAGGTTAGATTGGTATTGCAATGAAAGAA<br>GACAAAGCGGTAATGAGAGAGAGACAGGG<br>AACAAGCAGAGCATGGATTGAGTTTACTA<br>AAACATTAAACGACTCTGTTTTGTCTCTA<br>CCCATGCACTGCCTCTTCCCTGGCTCCCT<br>CTTTTTTTCTCTATATTTCTCTCTCTCCT<br>TTCATTTCACAGCTTTCAATGGAATTTTA<br>TTGCTACTGCTAACG	0	0	0	0	0	0	0	0	0	0	0	0
>ath-miR408 MI0001080<br>Arabidopsis thaliana miR408<br>stem-loop												
ATGCACTGCCTCTTCCCTGGC	36	537	512	28	81	435	298	1	1	0	3	6
>+1 ath-miR408												
CATGCACTGCCTCTTCCCTGGC	1	0	0	0	1	0	0	0	0	0	0	0
>+2 ath-miR408												
CCATGCACTGCCTCTTCCCTGGC	0	0	0	0	0	0	0	0	0	0	0	0
>+3 ath-miR408												
CCCATGCACTGCCTCTTCCCTGGC	0	0	0	0	0	0	0	0	0	0	0	0
>ath-MIR413 MI0001424<br>Arabidopsis thaliana miR413<br>stem-loop												
GATCCATAGTTTCTCTTGTTCTGCACATC<br>CACTAACTTCAGGAACCATGTCCCAGTTT<br>CAGGTTAGATCAAGTGGGAAAACAAATGG<br>AAGATTGTGGCTATGCAGAAACTGGAACT<br>ATTCGTC	0	0	0	0	0	0	0	0	0	0	0	0
>ath-miR413 MI0001424<br>Arabidopsis thaliana miR413<br>stem-loop												
ATAGTTTCTCTTGTTCTGCAC	0	0	0	0	0	0	0	0	0	0	0	0
>+1 ath-miR413												
CATAGTTTCTCTTGTTCTGCAC	0	0	0	0	0	0	0	0	0	0	0	0
>+2 ath-miR413												
CCATAGTTTCTCTTGTTCTGCAC	0	0	0	0	0	0	0	0	0	0	0	0
>+3 ath-miR413												
TCCATAGTTTCTCTTGTTCTGCAC	0	0	0	0	0	0	0	0	0	0	0	0
>ath-MIR414 MI0001425<br>Arabidopsis thaliana miR414<br>stem-loop												
TCATCATTATCATCATCATCATATTCATC<br>TTCATCATCATCGTCATCATCATCATCAT<br>CATCGTATGAGAAGATAGAGAAGAGTGAG<br>AGTATGAGATTTGAGTTGAGA	0	0	0	0	0	0	0	0	0	0	0	0
>ath-miR414 MI0001425<br>Arabidopsis thaliana miR414<br>stem-loop												
TCATCTTCATCATCATCGTCA	0	0	0	0	0	0	0	0	0	0	0	0
>+1 ath-miR414												
TTCATCTTCATCATCATCGTCA	0	0	0	0	0	0	0	0	0	0	0	0
>+2 ath-miR414												
ATTCATCTTCATCATCATCGTCA	0	0	0	0	0	0	0	0	0	0	0	0
>+3 ath-miR414												
TATTCATCTTCATCATCATCGTCA	0	0	0	0	0	0	0	0	0	0	0	0
>ath-MIR415 MI0001426<br>Arabidopsis thaliana miR415<br>stem-loop												
AGAACAGAGCAGAAACAGAACATATATTC<br>TCTGTCTTTTTTTGTGGCAAAAGTAAATG<br>GCGAGAAGACAACTCTTTTACCATCGAAT<br>GTTCTTGGTTTTAGCTCTTCTCT	0	0	0	0	0	0	0	0	0	0	0	0
>ath-miR415 MI0001426<br>Arabidopsis thaliana miR415<br>stem-loop												
AACAGAGCAGAAACAGAACAT	0	0	0	0	0	0	0	0	0	0	0	0
>+1 ath-miR415												
GAACAGAGCAGAAACAGAACAT	0	0	0	0	0	0	0	0	0	0	0	0
>+2 ath-miR415												
AGAACAGAGCAGAAACAGAACAT	0	0	0	0	0	0	0	0	0	0	0	0
>ath-MIR416 MI0001427<br>Arabidopsis thaliana miR416<br>stem-loop												
CGAAACTGAACCCGGTTTGTACGTATGGA<br>CCGCGTCGTTGGAATCCAAAAGAACCAGG<br>TTCGTACGTACACTGTTCATCG	0	0	0	0	0	0	0	0	0	0	0	0
>ath-miR416 MI0001427<br>Arabidopsis thaliana miR416<br>stem-loop												
GGTTCGTACGTACACTGTTCA	0	0	0	0	0	0	0	0	0	0	0	0

>+1 ath-miR416												
AGGTTCGTACGTACACTGTTCA	0	0	0	0	0	0	0	0	0	0	0	0
>+2 ath-miR416												
CAGGTTCGTACGTACACTGTTCA	0	0	0	0	0	0	0	0	0	0	0	0
>+3 ath-miR416												
CCAGGTTCGTACGTACACTGTTCA	0	0	0	0	0	0	0	0	0	0	0	0
>ath-MIR417 MI0001428 Arabidopsis thaliana miR417 stem-loop												
AAATATATTCAAAAGTGGTCAAAACACGTCACTATTTCCTTTATGTTTTCCCCTTATTGATGGAAATGGTTAAACATGAAGGTAGTGAATTTGTTCGAATAATATGTTGATATATTT	0	0	0	0	0	0	0	0	0	0	0	0
>ath-miR417 MI0001428 Arabidopsis thaliana miR417 stem-loop												
GAAGGTAGTGAATTTGTTCGA	0	0	0	0	0	0	0	0	0	0	0	0
>+1 ath-miR417												
TGAAGGTAGTGAATTTGTTCGA	0	0	0	0	0	0	0	0	0	0	0	0
>+2 ath-miR417												
ATGAAGGTAGTGAATTTGTTCGA	0	0	0	0	0	0	0	0	0	0	0	0
>+3 ath-miR417												
CATGAAGGTAGTGAATTTGTTCGA	0	0	0	0	0	0	0	0	0	0	0	0
>ath-MIR418 MI0001429 Arabidopsis thaliana miR418 stem-loop												
TTTAAATTTAGAATCTAGCGTAAAAAGAAAATCCGAATCAGGAACTCTAATGTGATGATGAACTGACCTTAGA	0	0	0	0	0	0	0	0	0	0	0	0
>ath-miR418 MI0001429 Arabidopsis thaliana miR418 stem-loop												
TAATGTGATGATGAACTGACC	0	0	0	0	0	0	0	0	0	0	0	0
>+1 ath-miR418												
CTAATGTGATGATGAACTGACC	0	0	0	0	0	0	0	0	0	0	0	0
>+2 ath-miR418												
TCTAATGTGATGATGAACTGACC	0	0	0	0	0	0	0	0	0	0	0	0
>+3 ath-miR418												
CTCTAATGTGATGATGAACTGACC	0	0	0	0	0	0	0	0	0	0	0	0
>ath-MIR419 MI0001430 Arabidopsis thaliana miR419 stem-loop												
AAATTATGAATGCTGAGGATGTTGTTATTACGAGCAATGAGATGTCTTTTTTTAAAAAAAAAAATTTGGTTGCTTGCTTGCAAGAGGACATCTTAGCATCAAATTT	0	0	0	0	0	0	0	0	0	0	0	0
>ath-miR419 MI0001430 Arabidopsis thaliana miR419 stem-loop												
TTATGAATGCTGAGGATGTTG	0	0	0	0	0	0	0	0	0	0	0	0
>+1 ath-miR419												
ATTATGAATGCTGAGGATGTTG	0	0	0	0	0	0	0	0	0	0	0	0
>+2 ath-miR419												
AATTATGAATGCTGAGGATGTTG	0	0	0	0	0	0	0	0	0	0	0	0
>+3 ath-miR419												
AAATTATGAATGCTGAGGATGTTG	0	0	0	0	0	0	0	0	0	0	0	0
>ath-MIR420 MI0001431 Arabidopsis thaliana miR420 stem-loop												
TTCTAAACTAATCACGGAAATGCAAAAATTGGATACTACACTATCAACTTTTTTCTTTTGTTCTCTTTATCTCGTGAATTTCCATTTAATGAGTTTAGTT	0	0	0	0	0	0	0	0	0	0	0	0
>ath-miR420 MI0001431 Arabidopsis thaliana miR420 stem-loop												
TAAACTAATCACGGAAATGCA	0	0	0	0	0	0	0	0	0	0	0	0
>+1 ath-miR420												
CTAAACTAATCACGGAAATGCA	0	0	0	0	0	0	0	0	0	0	0	0
>+2 ath-miR420												
TCTAAACTAATCACGGAAATGCA	0	0	0	0	0	0	0	0	0	0	0	0
>+3 ath-miR420												
TTCTAAACTAATCACGGAAATGCA	0	0	0	0	0	0	0	0	0	0	0	0
>ath-MIR426 MI0001441 Arabidopsis thaliana miR426 stem-loop												
GAGGGGGGACAATTTTTGGAAATTTGTCCTTACGGGTAGTACTAGAATACTTGTCCACATGACGATTTGAAATAACCGAAGGACATATGAGGAAAAAATAAAATTAAACTC	0	0	0	0	0	0	0	0	0	0	0	0
>ath-miR426 MI0001441 Arabidopsis thaliana miR426 stem-loop												
TTTTGGAAATTTGTCCTTACG	0	0	0	0	0	0	0	0	0	0	0	0
>+1 ath-miR426												
TTTTTGGAAATTTGTCCTTACG	0	0	0	0	0	0	0	0	0	0	0	0
>+2 ath-miR426												

ATTTTTGGAAATTTGTCCTTACG	0	0	0	0	0	0	0	0	0	0	0	0
>+3 ath-miR426												
AATTTTTGGAAATTTGTCCTTACG	0	0	0	0	0	0	0	0	0	0	0	0
>ath-MIR447a MI0002407 Arabidopsis thaliana miR447a stem-loop												
CATTCTTAATATATAATACTACTTTTTCA<br>TCCATTAAACCCCTTACAATGTCGAGTAA<br>ACGAAGCATCTGTCCCCTGGTATTGTCTT<br>CGAGCTTGGTGTTTTTTTCTAGCCAACTC<br>CAAGTTCTCGAGTTGATCATTGTTTGTAT<br>TCTTGAGACATTATTTGGGGACGAGATGT<br>TTTGTTGACTCGATATAAGAAGGGGCTTT<br>ATGGAAGAAATTGTAGTATTATATATCGA<br>GAGTG	0	0	0	0	0	0	0	0	0	0	0	0
>ath-miR447a MI0002407 Arabidopsis thaliana miR447a stem-loop												
TTGGGGACGAGATGTTTTGTTG	29	324	324	35	30	59	66	0	0	1	0	0
>+1 ath-miR447a												
TTTGGGGACGAGATGTTTTGTTG	3	30	24	3	1	4	9	0	0	0	0	0
>+2 ath-miR447a												
ATTTGGGGACGAGATGTTTTGTTG	0	0	0	0	0	0	0	0	0	0	0	0
>+3 ath-miR447a												
TATTTGGGGACGAGATGTTTTGTTG	0	0	0	0	0	0	0	0	0	0	0	0
>ath-MIR447b MI0002408 Arabidopsis thaliana miR447b stem-loop												
CATTCTTAATATACAATACTACTTTTTCA<br>TCCATTAATCCCCTTACAATGTCGAGTAA<br>ACGAAGCATCTGTCCCCTGGTATTGTCTT<br>CGAGCTTGGTGTGTTTTTCTAGCCAGCCC<br>CAAGTTCTCGAGTTGATCATTGTTTGTAT<br>TCTGACACATTATTTGGGGACGAGATGTT<br>TTGTTGACTCGATATAAGAAGGGGCTTTA<br>TGGAAGAAATTGTAGTATTATATATTGAG<br>AATG	0	0	0	0	0	0	0	0	0	0	0	0
>ath-miR447b MI0002408 Arabidopsis thaliana miR447b stem-loop												
TTGGGGACGAGATGTTTTGTTG	29	324	324	35	30	59	66	0	0	1	0	0
>+1 ath-miR447b												
TTTGGGGACGAGATGTTTTGTTG	3	30	24	3	1	4	9	0	0	0	0	0
>+2 ath-miR447b												
ATTTGGGGACGAGATGTTTTGTTG	0	0	0	0	0	0	0	0	0	0	0	0
>+3 ath-miR447b												
TATTTGGGGACGAGATGTTTTGTTG	0	0	0	0	0	0	0	0	0	0	0	0
>ath-MIR447c MI0002409 Arabidopsis thaliana miR447c stem-loop												
CATTCTTAATATACAATACTTCTTTTTCA<br>TGCATTAAGCCCCTTACAATGTCGAGTAA<br>ACAAAGCATGTGTCCGCTAATATTGTCTT<br>CGAGCTTGGTATTTTTGTATTCTGATACG<br>GTATTTGGGGACGACATCTTTTGTTGACT<br>CGATATAAGAAGGGGGTTTGTGGAAGAAA<br>TTGTAGTATTATATATCAAGAATG	0	0	0	0	0	0	0	0	0	0	0	0
>ath-miR447c MI0002409 Arabidopsis thaliana miR447c stem-loop												
TTGGGGACGACATCTTTTGTTG	1	0	0	0	0	0	0	0	0	0	0	0
>+1 ath-miR447c												
TTTGGGGACGACATCTTTTGTTG	0	0	0	0	0	0	0	0	0	0	0	0
>+2 ath-miR447c												
ATTTGGGGACGACATCTTTTGTTG	0	0	0	0	0	0	0	0	0	0	0	0
>+3 ath-miR447c												
TATTTGGGGACGACATCTTTTGTTG	0	0	0	0	0	0	0	0	0	0	0	0
>ath-MIR472 MI0005102 Arabidopsis thaliana miR472 stem-loop												
TCTGTATGTATGTATGGTCGAAGTAGGCA<br>AAATCTCACCTTTCTAGCAGATCAACAAT<br>GAATTTTGTGGAATAGATGTTGGATTTGT<br>AAGGTTTTGGTGTTTGCAAGTGTTGAGAT<br>GCGCAAGATTTGAGTAAGATTTTTCCTAC<br>TCCGCCCATACCATACATACACC	0	0	0	0	0	0	0	0	0	0	0	0
>ath-miR472 MI0005102 Arabidopsis thaliana miR472 stem-loop												
TTTTTCCTACTCCGCCCATACC	7	3	6	5	3	7	9	0	1	0	0	1
>+1 ath-miR472												
ATTTTTCCTACTCCGCCCATACC	0	0	0	0	0	0	0	0	0	0	0	0
>+2 ath-miR472												
GATTTTTCCTACTCCGCCCATACC	0	0	0	0	0	0	0	0	0	0	0	0
>+3 ath-miR472												
AGATTTTTCCTACTCCGCCCATACC	0	0	0	0	0	0	0	0	0	0	0	0

>ath-MIR771 MI0005101<br>Arabidopsis thaliana miR771<br>stem-loop												
TAAATTCGTCATGAGCCTCTGTGGTAGCC<br>CTCACAATGATCTCAATAGAGTGCATGCC<br>ACGATCTTGTAAAGACGTAGATGAGGGCT<br>CCTCAGGTGTTCATGTCGAATGTTAG	0	0	0	0	0	0	0	0	0	0	0	0
>ath-miR771 MI0005101<br>Arabidopsis thaliana miR771<br>stem-loop												
TGAGCCTCTGTGGTAGCCCTCA	188	336	300	0	0	0	1	3	0	2	0	5
>+1 ath-miR771												
ATGAGCCTCTGTGGTAGCCCTCA	0	0	0	0	0	0	0	0	0	0	0	0
>+2 ath-miR771												
CATGAGCCTCTGTGGTAGCCCTCA	0	0	0	0	0	0	0	0	0	0	0	0
>+3 ath-miR771												
TCATGAGCCTCTGTGGTAGCCCTCA	0	0	0	0	0	0	0	0	0	0	0	0
>ath-MIR773 MI0005103<br>Arabidopsis thaliana miR773<br>stem-loop												
AGGAGGCAATAGCTTGAGCAAATAATTGA<br>TTGCAGAAGTCCATCGACTAAAGCTGTCA<br>CCTGTTTGCTTCCAGCTTTTGTCTCCT	0	0	0	0	0	0	0	0	0	0	0	0
>ath-miR773 MI0005103<br>Arabidopsis thaliana miR773<br>stem-loop												
TTTGCTTCCAGCTTTTGTCTC	7	17	21	214	173	45	83	0	3	0	0	6
>+1 ath-miR773												
GTTTGCTTCCAGCTTTTGTCTC	0	0	0	0	0	0	0	0	0	0	0	0
>+2 ath-miR773												
TGTTTGCTTCCAGCTTTTGTCTC	0	0	0	1	0	0	0	0	0	0	0	0
>+3 ath-miR773												
CTGTTTGCTTCCAGCTTTTGTCTC	0	0	0	0	0	0	0	0	0	0	0	0
>ath-MIR774 MI0005104<br>Arabidopsis thaliana miR774<br>stem-loop												
TCATTTCAGATGGCTGTTTGGGTAACTAA<br>TATTTAAGATTTTGGTCAATTTAAGTTAC<br>CAATTTATATATTGGTTACCCATATGGCC<br>ATCTCAAATTT	0	0	0	0	0	0	0	0	0	0	0	0
>ath-miR774 MI0005104<br>Arabidopsis thaliana miR774<br>stem-loop												
TTGGTTACCCATATGGCCATC	3	0	3	0	2	0	0	0	0	2	0	1
>+1 ath-miR774												
ATTGGTTACCCATATGGCCATC	0	0	0	0	0	0	0	0	0	0	0	0
>+2 ath-miR774												
TATTGGTTACCCATATGGCCATC	0	0	0	0	0	0	0	0	0	0	0	0
>+3 ath-miR774												
ATATTGGTTACCCATATGGCCATC	0	0	0	0	0	0	0	0	0	0	0	0
>ath-MIR775 MI0005105<br>Arabidopsis thaliana miR775<br>stem-loop												
TTTAAACGTTGCACTACGTGACATTGAAA<br>CTGTCTTTCAACATTCCAATATTTCAACT<br>TTCGAATACCCAATATTTGGTTTGTTCAA<br>AGACATTTTCGATGTCTAGCAGTGCCAAT<br>GTTTAAA	0	0	0	0	0	0	0	0	0	0	0	0
>ath-miR775 MI0005105<br>Arabidopsis thaliana miR775<br>stem-loop												
TTCGATGTCTAGCAGTGCCA	130	1750	1745	2830	2671	653	973	7	9	6	0	9
>+1 ath-miR775												
TTTCGATGTCTAGCAGTGCCA	1	22	21	46	41	16	23	0	0	0	0	1
>+2 ath-miR775												
TTTTCGATGTCTAGCAGTGCCA	57	593	567	1081	927	426	562	3	0	3	0	15
>+3 ath-miR775												
ATTTTCGATGTCTAGCAGTGCCA	0	0	0	0	1	1	0	0	0	0	0	0
>ath-MIR776 MI0005106<br>Arabidopsis thaliana miR776<br>stem-loop												
TTGGGATTATAGCCACGAACTCAATAGAA<br>TTCTTAAGATCATGTGAAAACGTTGACAA<br>GATCGTCTACGATTTTCCAAGATTCTAAG<br>TCTTCTATTGATGTTCATGGCTTTAACCT<br>GA	0	0	0	0	0	0	0	0	0	0	0	0
>ath-miR776 MI0005106<br>Arabidopsis thaliana miR776<br>stem-loop												
TCTAAGTCTTCTATTGATGTT	3	2	2	0	0	0	0	4	0	2	0	19
>+1 ath-miR776												
TTCTAAGTCTTCTATTGATGTT	0	0	0	0	0	0	0	0	0	0	0	0
>+2 ath-miR776												
ATTCTAAGTCTTCTATTGATGTT	0	0	0	0	0	0	0	0	0	0	0	0
>+3 ath-miR776												
GATTCTAAGTCTTCTATTGATGTT	0	0	0	0	0	0	0	0	0	0	0	0

>ath-MIR777 MI0005107<br>Arabidopsis thaliana miR777<br>stem-loop												
AGAGAGCAACAAAATCAGTGTGTATTTGT<br>TAAGACAAATGTTTATATAAGTCGTCCAT<br>GCGTTGTACATTTAATCTTAACTAATACG<br>CATTGAGTTTCGTTGCTTTCT	0	0	0	0	0	0	0	0	0	0	0	0
>ath-miR777 MI0005107<br>Arabidopsis thaliana miR777<br>stem-loop												
TACGCATTGAGTTTCGTTGCTT	22	29	12	25	42	29	35	0	0	1	0	1
>+1 ath-miR777												
ATACGCATTGAGTTTCGTTGCTT	1	1	0	0	0	0	1	0	0	0	0	0
>+2 ath-miR777												
AATACGCATTGAGTTTCGTTGCTT	0	0	1	0	0	0	0	0	0	0	0	0
>+3 ath-miR777												
TAATACGCATTGAGTTTCGTTGCTT	0	0	0	0	0	0	0	0	0	0	0	0
>ath-MIR778 MI0005108<br>Arabidopsis thaliana miR778<br>stem-loop												
CCGCCTTGGCTTGGTTTATGTACACCGAG<br>TATGTTCAGCTCCATCCTATACAGATTTT<br>GGGACATGTGCCTAGGGTTGAGGTTTCAT<br>ATATAGAAAGAACTGAACAAACTCGGTGT<br>ACATAGACCAAACCAAGGCGGT	0	0	0	0	0	0	0	0	0	0	0	0
>ath-miR778 MI0005108<br>Arabidopsis thaliana miR778<br>stem-loop												
TGGCTTGGTTTATGTACACCG	12	5	3	25	0	0	0	0	0	0	0	0
>+1 ath-miR778												
TTGGCTTGGTTTATGTACACCG	13	4	7	93	0	0	0	0	0	0	0	0
>+2 ath-miR778												
CTTGGCTTGGTTTATGTACACCG	0	0	0	0	0	0	0	0	0	0	0	0
>+3 ath-miR778												
CCTTGGCTTGGTTTATGTACACCG	0	0	0	0	0	0	0	0	0	0	0	0
>ath-MIR779 MI0005109<br>Arabidopsis thaliana miR779<br>stem-loop												
AATGAGAGTAATATAGTTGAATCTTCGAA<br>AGTATTTCAAGAAGTCAGCTGAGCTTTCT<br>CGTCATCACTTATTAGTAAATATAGTCTC<br>TATATTTTTGATGAGTGATGATTGGAAAT<br>TTCGTTGACTCATTACTCTTGATGATTCT<br>GCTATGTTGCTGCTCATT	0	0	0	0	0	0	0	0	0	0	0	0
>ath-miR779.1 MI0005109<br>Arabidopsis thaliana miR779<br>stem-loop												
TTCTGCTATGTTGCTGCTCAT	0	1	1	1	1	0	0	0	0	0	0	1
>+1 ath-miR779.1												
ATTCTGCTATGTTGCTGCTCAT	0	0	0	0	0	0	0	0	0	0	0	0
>+2 ath-miR779.1												
GATTCTGCTATGTTGCTGCTCAT	0	0	0	0	0	0	0	0	0	0	0	0
>+3 ath-miR779.1												
TGATTCTGCTATGTTGCTGCTCAT	0	0	0	0	0	0	0	0	0	0	0	0
>ath-MIR779 MI0005109<br>Arabidopsis thaliana miR779<br>stem-loop												
AATGAGAGTAATATAGTTGAATCTTCGAA<br>AGTATTTCAAGAAGTCAGCTGAGCTTTCT<br>CGTCATCACTTATTAGTAAATATAGTCTC<br>TATATTTTTGATGAGTGATGATTGGAAAT<br>TTCGTTGACTCATTACTCTTGATGATTCT<br>GCTATGTTGCTGCTCATT	0	0	0	0	0	0	0	0	0	0	0	0
>ath-miR779.2 MI0005109<br>Arabidopsis thaliana miR779<br>stem-loop												
TGATTGGAAATTTCGTTGACT	3	67	65	21	28	25	27	0	0	0	0	1
>+1 ath-miR779.2	0.0%	6.0%	1.5%	0.0%	0.0%	0.0%	0.0%	#####	#####	#####	#DIV/0!	0.0%
ATGATTGGAAATTTCGTTGACT	0	4	1	0	0	0	0	0	0	0	0	0
>+2 ath-miR779.2												
GATGATTGGAAATTTCGTTGACT	0	0	0	0	0	0	0	0	0	0	0	0
>+3 ath-miR779.2												
TGATGATTGGAAATTTCGTTGACT	0	0	0	0	0	0	0	0	0	0	0	0
>ath-MIR780 MI0005110<br>Arabidopsis thaliana miR780<br>stem-loop												
CAAGATATCAGATATTTCACGAAGATATC<br>TGCATAACAGCTACGAGGAAATTGTGATT<br>TTATCGTTTCACAAACATCAACAGCTCCA<br>TGGATCGTTATGGACGGATAAATCACAAT<br>CACTATCCTTTTTCTAGCAGCTGTTGAGC<br>AGGTTTCTTCGTGAATATCTGGCATTTTG	0	0	0	0	0	0	0	0	0	0	0	0
>ath-miR780.1 MI0005110<br>Arabidopsis thaliana miR780<br>stem-loop												
TCTAGCAGCTGTTGAGCAGGT	845	2706	2517	4	9	16	12	2	0	1	0	0
>+1 ath-miR780.1	1.5%	2.0%	1.4%	0.0%	0.0%	6.3%	0.0%	0.0%	#####	0.0%	#DIV/0!	#####

TTCTAGCAGCTGTTGAGCAGGT	13	53	36	0	0	1	0	0	0	0	0	1
>+2 ath-miR780.1												
TTTCTAGCAGCTGTTGAGCAGGT	0	0	0	0	0	0	0	0	0	0	0	0
>+3 ath-miR780.1												
TTTTCTAGCAGCTGTTGAGCAGGT	0	0	0	0	0	0	0	0	0	0	0	0
>ath-MIR780 MI0005110 Arabidopsis thaliana miR780 stem-loop												
CAAGATATCAGATATTTCACGAAGATATCTGCATAACAGCTACGAGGAAATTGTGATTTTATCGTTTCACAAACATCAACAGCTCCATGGATCGTTATGGACGGATAAATCACAATCACTATCCTTTTTCTAGCAGCTGTTGAGCAGGTTTCTTCGTGAATATCTGGCATTTTG	0	0	0	0	0	0	0	0	0	0	0	0
>ath-miR780.2 MI0005110 Arabidopsis thaliana miR780 stem-loop												
TTCTTCGTGAATATCTGGCAT	2399	7545	7294	26	17	79	45	1	0	3	0	5
>+1 ath-miR780.2												
TTTCTTCGTGAATATCTGGCAT	459	1424	1435	4	1	19	8	0	0	2	0	0
>+2 ath-miR780.2												
GTTTCTTCGTGAATATCTGGCAT	0	0	0	0	0	0	0	0	0	0	0	0
>+3 ath-miR780.2												
GGTTTCTTCGTGAATATCTGGCAT	28	22	18	0	0	1	0	0	0	0	0	0
>ath-MIR781 MI0005111 Arabidopsis thaliana miR781 stem-loop												
TCAAATTAGAGTTTTCTGGATACTTATTAGTTACTAACAAAGAGAGGGCTTCTTCGATATTAACTTTTAAGTATCCAGAAAACTCTAAATTTGA	0	0	0	0	0	0	0	0	0	0	0	0
>ath-miR781 MI0005111 Arabidopsis thaliana miR781 stem-loop												
TTAGAGTTTTCTGGATACTTA	6	7	9	5	5	8	11	1	0	1	1	1
>+1 ath-miR781												
ATTAGAGTTTTCTGGATACTTA	0	0	0	0	0	0	0	0	0	0	0	0
>+2 ath-miR781												
AATTAGAGTTTTCTGGATACTTA	0	0	0	0	0	0	0	0	0	0	0	0
>+3 ath-miR781												
AAATTAGAGTTTTCTGGATACTTA	0	0	0	0	0	0	0	0	0	0	0	0
>ath-MIR782 MI0005112 Arabidopsis thaliana miR782 stem-loop												
AACCTTTTTCTACAAACACCTTGGATGTTCTTTAAATAAGATGAAAAGGCTATTATTCTAATGAACAACATGAAGCCAAAAGAAAATCCAAGGCGTTTGCAGAAAGAGGTT	0	0	0	0	0	0	0	0	0	0	0	0
>ath-miR782 MI0005112 Arabidopsis thaliana miR782 stem-loop												
ACAAACACCTTGGATGTTCTT	2	0	0	0	0	0	1	0	0	0	0	0
>+1 ath-miR782												
TACAAACACCTTGGATGTTCTT	0	0	0	0	0	0	0	0	0	0	0	0
>+2 ath-miR782												
CTACAAACACCTTGGATGTTCTT	0	0	0	0	0	0	0	0	0	0	0	0
>+3 ath-miR782												
TCTACAAACACCTTGGATGTTCTT	0	0	0	0	0	0	0	0	0	0	0	0
>ath-MIR783 MI0005113 Arabidopsis thaliana miR783 stem-loop												
GACACTAGTCTTAACCATATCTTTTGTCGGAAGATTCAGGAGAACATGGAAGAGCAAAGATTCTAGCAAATCTCTTACCCAATCAACTTCATCACCAGATCTTTTGTCGGAAAATTCAGGAGAACATGATCGTTTGGTACGAATACAAGATCTGGTGAGAATGGACGAAGAAGGAAAGAGAGACGTGTTCGTACCAAACGATCATGTTCTCCTGAATCTTCCGACAAAAGATCTGGTGATGAAGTTGATTGGTTAAGTGATTTACCAGAAGCTTTGCTCGTTCATGTTCTCCTGAATCTTCCGACAAAAAATGTGGTTAAGACTAGTGTCT	0	0	0	0	0	0	0	0	0	0	0	0
>ath-miR783 MI0005113 Arabidopsis thaliana miR783 stem-loop												
AAGCTTTGCTCGTTCATGTTC	3	0	0	0	0	0	0	0	0	0	0	0
>+1 ath-miR783												
GAAGCTTTGCTCGTTCATGTTC	0	1	1	0	0	1	0	0	0	0	0	0
>+2 ath-miR783												
AGAAGCTTTGCTCGTTCATGTTC	1	0	1	0	0	1	3	0	0	0	0	0
>+3 ath-miR783												
CAGAAGCTTTGCTCGTTCATGTTC	0	0	0	0	0	0	0	0	0	0	0	0
>ath-MIR822 MI0005379 Arabidopsis thaliana miR822 stem-loop												

CGACCTTAAGTATAAGTAGATATATGGGG<br>ATGTAACGCATGTTGTTTTCTGCGGGAAG<br>CATTTGCACATGTTTCGTGGAGAATGAAA<br>TCACATTCCATACATGAATAATAATTACC<br>TTTTAGATAGAACATCGTACTGCTTGAAA<br>AAACATGTTAATGTCATAAACTTTATGAT<br>GAAAACACCTATAAAAAGCGTTTTTTCAA<br>GCATGTGCTATCTATGAAGGTAATTACTA<br>TTCATGTATAAAATATGATTTTATCCTCC<br>ATAAAACATGTGCAAATGCTTTCTACAGG<br>AAACAATATACGTTGCATCCCCATCTACT<br>TACACTTAAGGTCGTTGT	0	0	0	0	0	0	0	0	0	0	0	0
>ath-miR822 MI0005379<br>Arabidopsis thaliana miR822<br>stem-loop												
TGCGGGAAGCATTTGCACATG	43	241	298	6038	3799	1391	1379	1	5	0	0	0
>+1 ath-miR822	2.3%	5.0%	4.4%	2.6%	1.6%	1.8%	3.0%	0.0%	0.0%	#####	#DIV/0!	#####
CTGCGGGAAGCATTTGCACATG	1	12	13	157	59	25	41	0	0	0	0	0
>+2 ath-miR822												
TCTGCGGGAAGCATTTGCACATG	1	1	2	45	26	0	4	0	0	0	0	0
>+3 ath-miR822												
TTCTGCGGGAAGCATTTGCACATG	0	0	0	3	2	0	0	0	0	0	0	0
>ath-MIR823 MI0005380<br>Arabidopsis thaliana miR823<br>stem-loop												
AATTTCAGAGATACTAATCCAAGAGATGG<br>CGGATACGTTTTACAATCACGAATCTTGT<br>ATGATCACTAACCATTGGAACAATAAGAG<br>TATATAATGAGTATATCACCATTTAGTTC<br>TAGTGGGTGGTGATCATATAAGATTGGTA<br>ATGGTAACAGTGAAACATATCCCCCATCT<br>ATTGGGATTAATTAGTC	0	0	0	0	0	0	0	0	0	0	0	0
>ath-miR823 MI0005380<br>Arabidopsis thaliana miR823<br>stem-loop												
TGGGTGGTGATCATATAAGAT	46	81	87	121	149	222	377	0	2	1	0	0
>+1 ath-miR823												
GTGGGTGGTGATCATATAAGAT	0	0	0	0	0	0	0	0	0	0	0	0
>+2 ath-miR823												
AGTGGGTGGTGATCATATAAGAT	0	0	0	0	0	0	0	0	0	0	0	0
>+3 ath-miR823												
TAGTGGGTGGTGATCATATAAGAT	0	0	0	0	0	0	0	0	0	0	0	0
>ath-MIR824 MI0005409<br>Arabidopsis thaliana miR824<br>stem-loop												
TATCACCATTTGTACTTGAGTTGTCTCTC<br>ATGTCTAGACCATTTGTGAGAAGGGAGTT<br>TTTGTTTACACCAATACCCCCCAGTCTCT<br>AAATTTGTAAGAAGTATTATGCTCAATTA<br>AGGAACATAGCTAGTTCGACATACCATAC<br>TACCCTTTTTAAAACTATCCTATATGTTT<br>GATGCTAGCATAGCGTAATTTTGTGTTCT<br>CATGGTGCAGCAGGGTTAGTTTATTGTGT<br>ACCTCTAGATATATATTCTCTTGCTGCGT<br>AGGGTTCCCAAGCTGCCAAAAACTTTTAA<br>AAATTAGTGATCTGTTCCCCAAACCCCCA<br>TTCAATAAGAAAGGTCTACCTGTAGCTCA<br>CAGTCACAGCTATTAGAGCTGTCCTTGCT<br>TCTTTGGGTTTACTGTTTTAGTTAATTAG<br>TTACCCTATGAAAGTCTGATCCTTCAGAA<br>GTTAGGTATAGAAGTAAGTCGATTAAGTT<br>CATTCATGACTTTTCCAGATGTTGCATAC<br>ATAACTTTTTTTATTTGTATTCGAACTAG<br>TCCAAGCCGATTCTCAAAATCATATAAAA<br>TCATTTACACCTTGGTCATGGTAGCTAAG<br>AATATTGTATCTAAAATTGGGGAGTGGGG<br>AGATGTTTGGTTATATTCCCTTCTCATCG<br>ATGGTCTAGATGTGCGAGGTGACTCTCAT<br>GGAGGTAAAGAACAATGGTGAT	0	0	0	0	0	0	0	0	0	0	0	0
>ath-miR824 MI0005409<br>Arabidopsis thaliana miR824<br>stem-loop												
TAGACCATTTGTGAGAAGGGA	124	445	384	2805	976	528	452	53	49	17	0	132
>+1 ath-miR824												
CTAGACCATTTGTGAGAAGGGA	0	0	0	3	1	2	1	0	0	0	0	0
>+2 ath-miR824												
TCTAGACCATTTGTGAGAAGGGA	0	0	0	0	0	1	0	0	0	0	0	0
>+3 ath-miR824												
GTCTAGACCATTTGTGAGAAGGGA	0	0	0	0	0	0	0	0	0	0	0	0
>ath-MIR825 MI0005381<br>Arabidopsis thaliana miR825<br>stem-loop												

CTTTCTATAATTACTTTTTGTATAGAATT<br>CACTAGTTGATTCCATCGACTCGTTCAAG<br>CACCAGCTCGAAGAAGCTTAGCTAATTTA<br>TCTTAGAAAATAATGAAAAAGCTATGCTT<br>CTCAAGAAGGTGCATGAACAAGTTGATGT<br>ATATTTTGATGTGTAAAGACCAAAATTGG<br>ATCTCACATGTG	0	0	0	0	0	0	0	0	0	0	0	0
>ath-miR825 MI0005381<br>Arabidopsis thaliana miR825<br>stem-loop												
TTCTCAAGAAGGTGCATGAAC	6	62	72	0	0	80	89	0	0	0	0	0
>+1 ath-miR825												
CTTCTCAAGAAGGTGCATGAAC	0	0	0	0	0	0	0	0	0	0	0	0
>+2 ath-miR825												
GCTTCTCAAGAAGGTGCATGAAC	0	0	0	0	0	0	0	0	0	0	0	0
>+3 ath-miR825												
TGCTTCTCAAGAAGGTGCATGAAC	0	0	0	0	0	0	0	0	0	0	0	0
>ath-MIR826 MI0005382<br>Arabidopsis thaliana miR826<br>stem-loop												
AGAGACGTGGATGCTTCTCGTCCACAAGT<br>TCTTTGGTGCCTCTATGATATAGTCCGGT<br>TTTGGATACGTGAAAATCATATCATATTG<br>CTGCTGTGAAAAGTTAATTTTCACGTGTC<br>CAAAACCAGATTATCTCATATTGCTGCTG<br>TGTATCTTATCATCTTTTTCCTGCTACTC<br>TTAC	0	0	0	0	0	0	0	0	0	0	0	0
>ath-miR826 MI0005382<br>Arabidopsis thaliana miR826<br>stem-loop												
TAGTCCGGTTTTGGATACGTG	1	1	2	0	0	0	0	1	0	0	0	0
>+1 ath-miR826												
ATAGTCCGGTTTTGGATACGTG	0	0	0	0	0	0	0	0	0	0	0	0
>+2 ath-miR826												
TATAGTCCGGTTTTGGATACGTG	0	0	0	0	0	0	0	0	0	0	0	0
>+3 ath-miR826												
ATATAGTCCGGTTTTGGATACGTG	0	0	0	0	0	0	0	0	0	0	0	0
>ath-MIR827 MI0005383<br>Arabidopsis thaliana miR827<br>stem-loop												
TTCCATGAAACGTTATAGGTTTTTTTCTT<br>TCTCTCTTGCAACCCTTGAATGTGTTTGT<br>TGATTGATATCTACACATGTTGATCATCC<br>TTGTGTTGATCGATTGGTTTAGATGACCA<br>TCAACAAACTCTTTCGTGGTTTTGCATCG<br>CTTCTTGGAATCTCGATCATGTTTTTAGC<br>ATC	0	0	0	0	0	0	0	0	0	0	0	0
>ath-miR827 MI0005383<br>Arabidopsis thaliana miR827<br>stem-loop												
TTAGATGACCATCAACAAACT	0	58	46	5026	35	1950	72	0	1	0	0	0
>+1 ath-miR827	#####	0.0%	2.2%	2.0%	0.0%	1.3%	0.0%	#####	0.0%	#####	#DIV/0!	#####
TTTAGATGACCATCAACAAACT	0	0	1	103	0	26	0	0	0	0	0	0
>+2 ath-miR827												
GTTTAGATGACCATCAACAAACT	0	0	0	0	0	0	0	0	0	0	0	0
>+3 ath-miR827												
GGTTTAGATGACCATCAACAAACT	0	0	0	0	0	0	0	0	0	0	0	0
>ath-MIR828 MI0005384<br>Arabidopsis thaliana miR828<br>stem-loop												
GTGACAAAGTCACAAAAGTGATAAACTCT<br>CCTTTCCACTTTCTAATGTTTCTTGCTTA<br>AATGAGTATTCCATGTAAAAATGATTCAC<br>TCACTCGTATGGGTGGGTTGGGTTTTCTC<br>TTCATGAGATGCTCATTTGAGCAAGCAAT<br>GTTTGGAAGTGGGACTATTTAATATCATG<br>TATGAGTTTTATCTTTT	0	0	0	0	0	0	0	0	0	0	0	0
>ath-miR828 MI0005384<br>Arabidopsis thaliana miR828<br>stem-loop												
TCTTGCTTAAATGAGTATTCCA	0	0	0	0	0	24	11	0	0	0	0	0
>+1 ath-miR828												
TTCTTGCTTAAATGAGTATTCCA	0	0	0	0	0	0	0	0	0	0	0	0
>+2 ath-miR828												
TTTCTTGCTTAAATGAGTATTCCA	0	0	0	0	0	0	0	0	0	0	0	0
>+3 ath-miR828												
GTTTCTTGCTTAAATGAGTATTCCA	0	0	0	0	0	0	0	0	0	0	0	0
>ath-MIR829 MI0005385<br>Arabidopsis thaliana miR829<br>stem-loop												
ACGTCAAAATTGATTAACAAAAAGTCAAT<br>GAATCATTCTACCAATTGACTTGTACTTT<br>GAAGCTTTGATTTGAACCTGTCAATTGGT<br>ATCAAAGCTTCTAAATCTTTGAAGCTCTG<br>ATACCAAATGATGGAATCAAATTAAAGCT<br>TCAAGGTAGTAGTCATCTTGGCAGAGAAA<br>AGAAAAAGCACTTCGAGTTTACCATCTTG<br>AT	0	0	0	0	0	0	0	0	0	0	0	0

>ath-miR829.1 MI0005385 Arabidopsis thaliana miR829 stem-loop												
AGCTCTGATACCAAATGATGGAAT	102	77	104	773	676	79	91	0	0	0	0	0
>+1 ath-miR829.1												
AAGCTCTGATACCAAATGATGGAAT	0	0	0	2	0	1	0	0	0	0	0	0
>+2 ath-miR829.1												
GAAGCTCTGATACCAAATGATGGAAT	0	0	0	0	0	0	0	0	0	0	0	0
>+3 ath-miR829.1												
TGAAGCTCTGATACCAAATGATGGAAT	0	0	0	0	0	0	0	0	0	0	0	0
>ath-MIR829 MI0005385 Arabidopsis thaliana miR829 stem-loop												
ACGTCAAAATTGATTAACAAAAAGTCAAT<br>GAATCATTCTACCAATTGACTTGTACTTT<br>GAAGCTTTGATTTGAACCTGTCAATTGGT<br>ATCAAAGCTTCTAAATCTTTGAAGCTCTG<br>ATACCAAATGATGGAATCAAATTAAAGCT<br>TCAAGGTAGTAGTCATCTTGGCAGAGAAA<br>AGAAAAAGCACTTCGAGTTTACCATCTTG<br>AT	0	0	0	0	0	0	0	0	0	0	0	0
>ath-miR829.2 MI0005385 Arabidopsis thaliana miR829 stem-loop												
CAAATTAAAGCTTCAAGGTAG	0	2	0	38	30	0	1	0	0	0	0	0
>+1 ath-miR829.2												
TCAAATTAAAGCTTCAAGGTAG	0	0	0	0	0	0	0	0	0	0	0	0
>+2 ath-miR829.2												
ATCAAATTAAAGCTTCAAGGTAG	0	0	0	0	0	0	0	0	0	0	0	0
>+3 ath-miR829.2												
AATCAAATTAAAGCTTCAAGGTAG	0	0	0	0	0	0	0	0	0	0	0	0
>ath-MIR830 MI0005386 Arabidopsis thaliana miR830 stem-loop												
CAGAGTCTCGCTAGTGTCTTTACTAAACA<br>CAAAATCTGTAAACGCCTCGATCTCCTCT<br>TCTCCAAATAGTTTAGGTTAGCTGACATA<br>TAATAATTCAATAGACCCCAGTTTCTCCA<br>GATAACCTAACTATTTTGAGAAGAAGTGA<br>ACGAAGAGCGATTACAAGTTAGGAGAGTG<br>CTTGAATTTTGAAGAACATCG	0	0	0	0	0	0	0	0	0	0	0	0
>ath-miR830 MI0005386 Arabidopsis thaliana miR830 stem-loop												
TAACTATTTTGAGAAGAAGTG	5	5	7	2	4	10	7	0	0	0	0	0
>+1 ath-miR830												
CTAACTATTTTGAGAAGAAGTG	0	0	0	0	0	0	0	0	0	0	0	0
>+2 ath-miR830												
CCTAACTATTTTGAGAAGAAGTG	0	0	0	0	0	0	0	0	0	0	0	0
>+3 ath-miR830												
ACCTAACTATTTTGAGAAGAAGTG	0	0	0	0	0	0	0	0	0	0	0	0
>ath-MIR831 MI0005387 Arabidopsis thaliana miR831 stem-loop												
GCACAATGCGGACAGTGCTACAAGAAGAG<br>ATTTATAGAGTCTTAAGAGTCTCAAGAAG<br>CGTACAAGGAGATGAGGAGTGAATCACCA<br>AAACAAGTGGTTTTGGTTTGTGAATCACC<br>AAAACAAGTGGTTTTGGTTTGTGAATCAG<br>TATGGTTTACCCAAAACACTTGTTTGGTG<br>TGTTTACTTCTGATCTCTTCGTACTCTTC<br>TTGCGATTCTATGACACTCTACATGTAAC<br>ATCGGCATGGTTTACCAAAAAGCCT	0	0	0	0	0	0	0	0	0	0	0	0
>ath-miR831 MI0005387 Arabidopsis thaliana miR831 stem-loop												
TGATCTCTTCGTACTCTTCTTG	5	4	11	1	3	0	0	0	0	0	0	0
>+1 ath-miR831												
CTGATCTCTTCGTACTCTTCTTG	0	0	0	0	0	0	0	0	0	0	0	0
>+2 ath-miR831												
TCTGATCTCTTCGTACTCTTCTTG	0	0	0	0	0	0	0	0	0	0	0	0
>+3 ath-miR831												
TTCTGATCTCTTCGTACTCTTCTTG	0	0	0	0	0	0	0	0	0	0	0	0
>ath-MIR832 MI0005388 Arabidopsis thaliana miR832 stem-loop												

AAGAAAGAAAAAATAAGTGACAAGTAACAAACATGAGAAAGGTGAAACTTGCTGGGATCGGGAATCGAAAGATCCGAAAGCCAGTCTTTAGAGGATTCTGAACGGGCTAGTGAAAGTAAACTTGTGATGTGACCATATTGTGCAATATGAATGTAAGTTGGTGGTGTGGTCATCTCACAAGGTGGCTAAGTCATCTCACTTATTTTCTTAACGAAAGCATCAGCCCGTTTAGCATCATCTTCTTGAAGCTTGCCTTTCGCATCTTTTGATTCCCAATCCAAGCAAGGTTCACCTTTTTTTTTGCCATGACCTTTACAAAATATCCAAATTTTG	0	0	0	0	0	0	0	0	0	0	0	0
>ath-miR832-3p MI0005388 Arabidopsis thaliana miR832 stem-loop												
TTGATTCCCAATCCAAGCAAG	0	0	0	0	0	0	0	0	0	0	0	0
>+1 ath-miR832-3p												
TTTGATTCCCAATCCAAGCAAG	0	0	0	0	0	0	0	0	0	0	0	0
>+2 ath-miR832-3p												
TTTTGATTCCCAATCCAAGCAAG	0	0	0	0	0	0	0	0	0	0	0	0
>+3 ath-miR832-3p												
CTTTTGATTCCCAATCCAAGCAAG	0	0	0	0	0	0	0	0	0	0	0	0
>ath-MIR832 MI0005388 Arabidopsis thaliana miR832 stem-loop												
AAGAAAGAAAAAATAAGTGACAAGTAACAAACATGAGAAAGGTGAAACTTGCTGGGATCGGGAATCGAAAGATCCGAAAGCCAGTCTTTAGAGGATTCTGAACGGGCTAGTGAAAGTAAACTTGTGATGTGACCATATTGTGCAATATGAATGTAAGTTGGTGGTGTGGTCATCTCACAAGGTGGCTAAGTCATCTCACTTATTTTCTTAACGAAAGCATCAGCCCGTTTAGCATCATCTTCTTGAAGCTTGCCTTTCGCATCTTTTGATTCCCAATCCAAGCAAGGTTCACCTTTTTTTTTGCCATGACCTTTACAAAATATCCAAATTTTG	0	0	0	0	0	0	0	0	0	0	0	0
>ath-miR832-5p MI0005388 Arabidopsis thaliana miR832 stem-loop												
TGCTGGGATCGGGAATCGAAA	0	4	4	0	0	0	0	0	0	0	0	0
>+1 ath-miR832-5p												
TTGCTGGGATCGGGAATCGAAA	0	0	0	0	0	0	0	0	0	0	0	0
>+2 ath-miR832-5p												
CTTGCTGGGATCGGGAATCGAAA	0	0	0	0	0	0	0	0	0	0	0	0
>+3 ath-miR832-5p												
ACTTGCTGGGATCGGGAATCGAAA	0	0	0	0	0	0	0	0	0	0	0	0
>ath-MIR833 MI0005389 Arabidopsis thaliana miR833 stem-loop												
GGACGTAGATGGTACTCTTACAGATGGTGATCGTGAAAACAAACGGAGGAGTTTGTTTGTTGTACTCGGTCTAGTTCAAACCAAAACATCTTTGCAAATATGTATGGTTTGAGCTAGACCGATGTCAACAAACAAGCTGCTTCGTTTGTATTCACCATCAACATCTTTAAGAAACGCTGCACCACTC	0	0	0	0	0	0	0	0	0	0	0	0
>ath-miR833-3p MI0005389 Arabidopsis thaliana miR833 stem-loop												
TAGACCGATGTCAACAAACAAG	2	3	6	3	2	3	9	0	0	0	0	0
>+1 ath-miR833-3p												
CTAGACCGATGTCAACAAACAAG	0	0	0	0	0	0	0	0	0	0	0	0
>+2 ath-miR833-3p												
GCTAGACCGATGTCAACAAACAAG	0	0	0	0	0	0	0	0	0	0	0	0
>+3 ath-miR833-3p												
AGCTAGACCGATGTCAACAAACAAG	0	0	0	0	0	0	0	0	0	0	0	0
>ath-MIR833 MI0005389 Arabidopsis thaliana miR833 stem-loop												
GGACGTAGATGGTACTCTTACAGATGGTGATCGTGAAAACAAACGGAGGAGTTTGTTTGTTGTACTCGGTCTAGTTCAAACCAAAACATCTTTGCAAATATGTATGGTTTGAGCTAGACCGATGTCAACAAACAAGCTGCTTCGTTTGTATTCACCATCAACATCTTTAAGAAACGCTGCACCACTC	0	0	0	0	0	0	0	0	0	0	0	0
>ath-miR833-5p MI0005389 Arabidopsis thaliana miR833 stem-loop												
TGTTTGTTGTACTCGGTCTAGT	2	7	10	3	8	8	7	0	0	0	0	0
>+1 ath-miR833-5p												
TTGTTTGTTGTACTCGGTCTAGT	0	11	12	3	5	17	13	0	1	0	0	0
>+2 ath-miR833-5p												
TTTGTTTGTTGTACTCGGTCTAGT	0	0	0	0	0	0	0	0	0	0	0	0
>+3 ath-miR833-5p												
GTTTGTTTGTTGTACTCGGTCTAGT	0	0	0	0	0	0	0	0	0	0	0	0

>ath-MIR834 MI0005390<br>Arabidopsis thaliana miR834<br>stem-loop												
TACAACATATGTTACCAGTTGTGCTATCA<br>TTTTTTTCAGCGACAGCCAAAATCACCAC<br>CGCTTCTGCTACGAACATGTTAAGCCATC<br>CTTTTAACCGTTGCTGCCACCACTATTAC<br>GGCCATCACAAGAAAATTCATGCAAGTGG<br>AAACGGTGATAGTAGCAGCTGGTATGACA<br>CGTTGGTAGCAGTAGCGGTGGTAATGCCG<br>ATAGCGTGAGCGGCAATGGAAGCATTGGT<br>GGTGTCAGAAGCAGTCA	0	0	0	0	0	0	0	0	0	0	0	0
>ath-miR834 MI0005390<br>Arabidopsis thaliana miR834<br>stem-loop												
TGGTAGCAGTAGCGGTGGTAA	1	0	0	0	0	0	2	0	0	0	0	0
>+1 ath-miR834												
TTGGTAGCAGTAGCGGTGGTAA	0	0	0	0	0	0	0	0	0	0	0	0
>+2 ath-miR834												
GTTGGTAGCAGTAGCGGTGGTAA	0	0	0	0	0	0	0	0	0	0	0	0
>+3 ath-miR834												
CGTTGGTAGCAGTAGCGGTGGTAA	0	0	0	0	0	0	0	0	0	0	0	0
>ath-MIR835 MI0005391<br>Arabidopsis thaliana miR835<br>stem-loop												
TGCTCGATTCTTCAGATAGAAATTCTGGT<br>CATCTTTGTGTTTAGGCTTTTTCTTGCAT<br>ATGTTCTTTATCTCTATTGATTGATATTG<br>TTCAATGAGCAACATACTTTTCTCTTCTA<br>CCTGGATTTGTTTGATGGTTTTAAGAACT<br>CTGTTCTTTTTGACGTTTTACCCAACAAT<br>ATAACTTCTGTTGTAGTCATCTTCTGCTG<br>CAACCCATCCACAAAGCTTATCATGCAAG<br>ATTAAACCCTTTCTCGATGGGTTGCAAGA<br>GAACATCACTAAAATAGAAGTAACATTGT<br>TGGGGAAAAACGTCACAAATCCAGGTATT<br>AGAGAATAGGATGCTGATCGTTGAAAATA<br>ACTATCTATAAAGATGGAGAAGATACGCA<br>AGAAAGAGTTGTAAACACAAAGC	0	0	0	0	0	0	0	0	0	0	0	0
>ath-miR835-3p MI0005391<br>Arabidopsis thaliana miR835<br>stem-loop												
TGGAGAAGATACGCAAGAAAG	2	4	6	0	0	1	1	0	0	0	0	0
>+1 ath-miR835-3p												
ATGGAGAAGATACGCAAGAAAG	0	0	4	0	0	0	0	0	0	0	0	0
>+2 ath-miR835-3p												
GATGGAGAAGATACGCAAGAAAG	0	0	0	0	0	0	0	0	0	0	0	0
>+3 ath-miR835-3p												
AGATGGAGAAGATACGCAAGAAAG	0	1	0	0	0	0	0	0	0	0	0	0
>ath-MIR835 MI0005391<br>Arabidopsis thaliana miR835<br>stem-loop												
TGCTCGATTCTTCAGATAGAAATTCTGGT<br>CATCTTTGTGTTTAGGCTTTTTCTTGCAT<br>ATGTTCTTTATCTCTATTGATTGATATTG<br>TTCAATGAGCAACATACTTTTCTCTTCTA<br>CCTGGATTTGTTTGATGGTTTTAAGAACT<br>CTGTTCTTTTTGACGTTTTACCCAACAAT<br>ATAACTTCTGTTGTAGTCATCTTCTGCTG<br>CAACCCATCCACAAAGCTTATCATGCAAG<br>ATTAAACCCTTTCTCGATGGGTTGCAAGA<br>GAACATCACTAAAATAGAAGTAACATTGT<br>TGGGGAAAAACGTCACAAATCCAGGTATT<br>AGAGAATAGGATGCTGATCGTTGAAAATA<br>ACTATCTATAAAGATGGAGAAGATACGCA<br>AGAAAGAGTTGTAAACACAAAGC	0	0	0	0	0	0	0	0	0	0	0	0
>ath-miR835-5p MI0005391<br>Arabidopsis thaliana miR835<br>stem-loop												
TTCTTGCATATGTTCTTTATC	0	0	0	0	0	1	0	0	0	0	0	0
>+1 ath-miR835-5p												
TTTCTTGCATATGTTCTTTATC	0	0	0	0	0	0	0	0	0	0	0	0
>+2 ath-miR835-5p												
TTTTCTTGCATATGTTCTTTATC	0	0	0	0	0	0	0	0	0	0	0	0
>+3 ath-miR835-5p												
TTTTTCTTGCATATGTTCTTTATC	0	0	0	0	0	0	0	0	0	0	0	0
>ath-MIR836 MI0005392<br>Arabidopsis thaliana miR836<br>stem-loop												

GCAAAGCTGCCATTTTATAGTCCAAAACT<br>TAAATTTCGTGTTTGCAAGTCTCCACCCA<br>TCGAAGGAAACACTAGAAGAGATATAATT<br>TATTTGAGGGGAGAAATATTTGACACGGA<br>AGCATAGCTCCATATCCTTCAATGGAGGT<br>GTGGTCCTTCAACAAAAATACCCCCCTCT<br>TGAAACTCTGTTTCACCACACCTCCATTG<br>AAGGACCTGAAGCTATGCTTCCTTGTCAT<br>ATTCCTTACCATCAAATAAATGCTCTGTT<br>TTCCTGTGTTTCCTTTGATGCGTGGAGAC<br>TTTTAAAAACGAAATTTAAGTTTGGGCTA<br>AAATAAACGGCAGTTTTG	0	0	0	0	0	0	0	0	0	0	0	0
>ath-miR836 MI0005392<br>Arabidopsis thaliana miR836<br>stem-loop												
TCCTGTGTTTCCTTTGATGCGTGG	0	0	0	0	0	0	0	0	0	0	0	0
>+1 ath-miR836												
TTCCTGTGTTTCCTTTGATGCGTGG	0	0	0	0	0	0	0	0	0	0	0	0
>+2 ath-miR836												
TTTCCTGTGTTTCCTTTGATGCGTGG	0	0	0	0	0	0	0	0	0	0	0	0
>+3 ath-miR836												
TTTTCCTGTGTTTCCTTTGATGCGTGG	0	0	0	0	0	0	0	0	0	0	0	0
>ath-MIR837 MI0005393<br>Arabidopsis thaliana miR837<br>stem-loop												
TTTTTGATAAATACTTTTGTTTGCTTACA<br>AGTATAAAGTCATCATGTTTTTTTCATCA<br>GTTTCTTGTTCGTTTCAAAATTATGATTT<br>TTGTTTACTTACACTCATAATCTTGAAAC<br>GAACAAAAAACTGATGGAAAAACATGAGG<br>ACTTTATACTTGTAAATAAATAAAAATAT<br>TTATTAGAAA	0	0	0	0	0	0	0	0	0	0	0	0
>ath-miR837-3p MI0005393<br>Arabidopsis thaliana miR837<br>stem-loop												
AAACGAACAAAAAACTGATGG	1	56	65	908	1004	76	71	0	0	0	0	0
>+1 ath-miR837-3p												
GAAACGAACAAAAAACTGATGG	0	0	0	0	1	0	0	0	0	0	0	0
>+2 ath-miR837-3p												
TGAAACGAACAAAAAACTGATGG	0	0	0	1	0	0	0	0	0	0	0	0
>+3 ath-miR837-3p												
TTGAAACGAACAAAAAACTGATGG	0	0	0	3	2	0	0	0	0	0	0	0
>ath-MIR837 MI0005393<br>Arabidopsis thaliana miR837<br>stem-loop												
TTTTTGATAAATACTTTTGTTTGCTTACA<br>AGTATAAAGTCATCATGTTTTTTTCATCA<br>GTTTCTTGTTCGTTTCAAAATTATGATTT<br>TTGTTTACTTACACTCATAATCTTGAAAC<br>GAACAAAAAACTGATGGAAAAACATGAGG<br>ACTTTATACTTGTAAATAAATAAAAATAT<br>TTATTAGAAA	0	0	0	0	0	0	0	0	0	0	0	0
>ath-miR837-5p MI0005393<br>Arabidopsis thaliana miR837<br>stem-loop												
ATCAGTTTCTTGTTCGTTTCA	0	2	0	86	72	2	4	0	0	0	0	0
>+1 ath-miR837-5p												
CATCAGTTTCTTGTTCGTTTCA	0	0	0	0	1	0	0	0	0	0	0	0
>+2 ath-miR837-5p												
TCATCAGTTTCTTGTTCGTTTCA	0	0	0	0	0	0	0	0	0	0	0	0
>+3 ath-miR837-5p												
TTCATCAGTTTCTTGTTCGTTTCA	0	0	0	0	0	0	0	0	0	0	0	0
>ath-MIR838 MI0005394<br>Arabidopsis thaliana miR838<br>stem-loop												
TACTTTTCTAATATCACGAGGACTTACAT<br>GGCCTCAAGTCACCTGTGGTGTTGTGCAA<br>GAAGGAGAAGCAAAGTCTGTCTATGTATT<br>ATGAGATAGCTACTTCTATGGCTAGGATA<br>TATGTTGTACAAGACCGGCTTTTCTTCTA<br>CTTCTTGCACAACCTGAGGTTATTGAGGC<br>TATACAAGTCTTCTTCTATAATGTTATTT<br>ATTA	0	0	0	0	0	0	0	0	0	0	0	0
>ath-miR838 MI0005394<br>Arabidopsis thaliana miR838<br>stem-loop												
TTTTCTTCTACTTCTTGCACA	0	2	1	1	2	9	5	0	0	0	0	0
>+1 ath-miR838												
CTTTTCTTCTACTTCTTGCACA	0	0	0	0	0	0	0	0	0	0	0	0
>+2 ath-miR838												
GCTTTTCTTCTACTTCTTGCACA	0	0	0	0	0	0	0	0	0	0	0	0
>+3 ath-miR838												
GGCTTTTCTTCTACTTCTTGCACA	0	0	0	0	0	0	0	0	0	0	0	0
>ath-MIR839 MI0005395<br>Arabidopsis thaliana miR839<br>stem-loop												

CTCACTCATGTGAGCAGAAAGAGTAGCAT<br>GATATTTTCTTCAAGGTCTTTACCAACCT<br>TTCATCGTTCCCTTCTTTGCAATAACGCT<br>GTTTTGCAAAACCGTGATAGTGCTGAGCC<br>GATGAGCCTCTAATGAAATTATAGACTCA<br>TCGGCTCAGGACCATTGCGGTTGTGCAAA<br>ACGGTGTTATTGCTGAGAAGGGAACGCAT<br>GAGAGGTTGGTAAAGACCTCAATGGAATC<br>TTATGGTACTCTTTTTGCTCACATGAGTG<br>AGT	0	0	0	0	0	0	0	0	0	0	0	0
>ath-miR839 MI0005395<br>Arabidopsis thaliana miR839<br>stem-loop												
TACCAACCTTTCATCGTTCCC	58	9	17	4	4	18	6	0	0	0	1	0
>+1 ath-miR839	1.7%	0.0%	0.0%	0.0%	75.0%	0.0%	0.0%	#####	#####	#####	0.0%	#####
TTACCAACCTTTCATCGTTCCC	1	0	0	0	3	0	0	0	0	0	0	0
>+2 ath-miR839												
TTTACCAACCTTTCATCGTTCCC	2	0	1	0	0	0	0	0	0	0	0	0
>+3 ath-miR839												
CTTTACCAACCTTTCATCGTTCCC	0	0	0	0	0	0	0	0	0	0	0	0
>ath-MIR840 MI0005396<br>Arabidopsis thaliana miR840<br>stem-loop												
CGTAAAGCAGGTGATTCACCAATTTAGGT<br>TTACATCCACAGTGTGGAAGACACTGAAG<br>GACCTAAACTAACAAAGGTAAACGGCTCA<br>GTGTGCGGGGTATTACACTCGGTTTAATG<br>TCTGAATGCGATAATCCGCACGATGATCT<br>CTTTATCTTTGTTTGTTTAGGTCCCTTAG<br>TTTCTTCTATACCGTGAATCCAATCCTGT<br>ATTGGATGAGCTGGTTTATGACC	0	0	0	0	0	0	0	0	0	0	0	0
>ath-miR840 MI0005396<br>Arabidopsis thaliana miR840<br>stem-loop												
ACACTGAAGGACCTAAACTAAC	1	10	7	0	0	8	10	0	0	0	0	0
>+1 ath-miR840												
GACACTGAAGGACCTAAACTAAC	0	0	0	0	0	0	0	0	0	0	0	0
>+2 ath-miR840												
AGACACTGAAGGACCTAAACTAAC	1	0	0	0	0	0	0	0	0	0	0	0
>+3 ath-miR840												
AAGACACTGAAGGACCTAAACTAAC	0	0	0	0	0	0	0	0	0	0	0	0
>ath-MIR841 MI0005397<br>Arabidopsis thaliana miR841<br>stem-loop												
GCACCAACACTACTATGTGCAGAAACTCT<br>GTTCTTAAGTTGCTTGTGAATACGAGCCA<br>CTTGAAACTGAAAGAAACAAAGAAACAAT<br>ATCAGTATAAAATTTTATCAAATTATACT<br>ACAAAAAATGAAATTTCTTTGTTTATTTC<br>AATTTCTAGTGGGTCGTATTCACATGCAA<br>CTCAAGACTAGAGTATCTACAACATGGTC<br>GACTTGGTG	0	0	0	0	0	0	0	0	0	0	0	0
>ath-miR841 MI0005397<br>Arabidopsis thaliana miR841<br>stem-loop												
TACGAGCCACTTGAAACTGAA	0	2	1	0	0	28	35	0	0	0	0	0
>+1 ath-miR841												
ATACGAGCCACTTGAAACTGAA	0	0	1	0	0	0	0	0	0	0	0	0
>+2 ath-miR841												
AATACGAGCCACTTGAAACTGAA	0	0	0	0	0	0	0	0	0	0	0	0
>+3 ath-miR841												
GAATACGAGCCACTTGAAACTGAA	0	0	0	0	0	0	0	0	0	0	0	0
>ath-MIR842 MI0005398<br>Arabidopsis thaliana miR842<br>stem-loop												
CAGTACCGTTCAGGGTGACAGAAACATTT<br>TCGAAAAGAGAGGCTATAAGCGGGATGAT<br>GAATCCGACCATGATGGTGTTGCAGAAAT<br>TTATGTATGATGTAGTTATTGTCCAGGGA<br>TCGAAACACCATCATGGTCAGATCCGTCA<br>TCCCACTTATGTTTTCTTAGGTCAACAAA<br>TATGGACAATTGAAAAGTGAAGCTC	0	0	0	0	0	0	0	0	0	0	0	0
>ath-miR842 MI0005398<br>Arabidopsis thaliana miR842<br>stem-loop												
TCATGGTCAGATCCGTCATCC	1	0	0	113	78	7	8	0	0	0	0	0
>+1 ath-miR842												
ATCATGGTCAGATCCGTCATCC	0	0	0	2	0	0	0	0	0	0	0	0
>+2 ath-miR842												
CATCATGGTCAGATCCGTCATCC	0	0	0	1	1	0	0	0	0	0	0	0
>+3 ath-miR842												
CCATCATGGTCAGATCCGTCATCC	0	0	0	0	0	0	0	0	0	0	0	0
>ath-MIR843 MI0005399<br>Arabidopsis thaliana miR843<br>stem-loop												

AATGCCCAACAACTTCGCGTTATTTCTGA<br>AAACTTCATATCTATTTTCTTTTAGGTCG<br>AGCTTCATTGGAACCAAATGGTGGCTCAT<br>CCATCTATTACTAAAGCTATACAGCAGGT<br>GGACAAGCCATCATCATGTTCCTGTGAAG<br>CTCGATCTAAAAGACTACAGATTTGAGAT<br>TTTCAAAAATAACATGAAGTACATTCTCC<br>AC	0	0	0	0	0	0	0	0	0	0	0	0
>ath-miR843 MI0005399<br>Arabidopsis thaliana miR843<br>stem-loop												
TTTAGGTCGAGCTTCATTGGA	3	54	44	0	0	95	39	0	0	0	0	0
>+1 ath-miR843												
TTTTAGGTCGAGCTTCATTGGA	0	0	0	0	0	1	0	0	0	0	0	0
>+2 ath-miR843												
CTTTTAGGTCGAGCTTCATTGGA	0	0	0	0	0	0	0	0	0	0	0	0
>+3 ath-miR843												
TCTTTTAGGTCGAGCTTCATTGGA	0	0	0	0	0	0	0	0	0	0	0	0
>ath-MIR844 MI0005400<br>Arabidopsis thaliana miR844<br>stem-loop												
GGAAGGAGATGTTTGCAGTTGGTTTAATG<br>AATCTTTTACATTGAAGAGAAGGAATGGT<br>AAGATTGCTTATAAGCTGGATCTTGAGGG<br>TGAGTGGTCTCTTCTACGCATTGGGCTTA<br>TAAGCCATCTTACTAGTTCTTCTTTTCAA<br>TGTTGTCTAAGGACGCTGCTTTTATCAGG<br>TGATCATTTGT	0	0	0	0	0	0	0	0	0	0	0	0
>ath-miR844 MI0005400<br>Arabidopsis thaliana miR844<br>stem-loop												
TGGTAAGATTGCTTATAAGCT	0	2	7	2	2	13	8	0	0	0	0	0
>+1 ath-miR844												
ATGGTAAGATTGCTTATAAGCT	0	1	0	0	0	2	0	0	1	0	0	0
>+2 ath-miR844												
AATGGTAAGATTGCTTATAAGCT	0	0	0	0	0	0	0	0	0	0	0	0
>+3 ath-miR844												
GAATGGTAAGATTGCTTATAAGCT	0	0	0	0	0	0	0	0	0	0	0	0
>ath-MIR845a MI0005401<br>Arabidopsis thaliana miR845a<br>stem-loop												
AGATAAGGGATTGCACGATTTCTCAATAG<br>TTCCGTCGCTGCGTTTCCATCACGTCGAT<br>TGCTGTCAGAGCCACGCCTATGAAGAATT<br>TGTGCGGCTCTGATACCAATTGATGCTAT<br>GGAAACATCGATATTGCCTGATTCCGTGT<br>TTCCTTTTACCTTGTTGT	0	0	0	0	0	0	0	0	0	0	0	0
>ath-miR845a MI0005401<br>Arabidopsis thaliana miR845a<br>stem-loop												
CGGCTCTGATACCAATTGATG	35	60	39	1	0	1	0	0	0	0	0	0
>+1 ath-miR845a												
GCGGCTCTGATACCAATTGATG	0	0	0	0	0	0	0	0	0	0	0	0
>+2 ath-miR845a												
TGCGGCTCTGATACCAATTGATG	0	0	0	0	0	0	0	0	0	0	0	0
>+3 ath-miR845a												
GTGCGGCTCTGATACCAATTGATG	0	0	0	0	0	0	0	0	0	0	0	0
>ath-MIR845b MI0005444<br>Arabidopsis thaliana miR845b<br>stem-loop												
AGTCGGTTTCCATCACGTCAATTGCTATC<br>AGAGCAACGCATGATGTATAATAACTAAG<br>CGTCGCTCTGATACCAAATTGATGCAATG<br>GAAACATCGATT	0	0	0	0	0	0	0	0	0	0	0	0
>ath-miR845b MI0005444<br>Arabidopsis thaliana miR845b<br>stem-loop												
TCGCTCTGATACCAAATTGATG	6	13	12	0	0	0	0	0	0	0	0	0
>+1 ath-miR845b												
GTCGCTCTGATACCAAATTGATG	0	0	0	0	0	0	0	0	0	0	0	0
>+2 ath-miR845b												
CGTCGCTCTGATACCAAATTGATG	0	0	0	0	0	0	0	0	0	0	0	0
>+3 ath-miR845b												
GCGTCGCTCTGATACCAAATTGATG	0	0	0	0	0	0	0	0	0	0	0	0
>ath-MIR846 MI0005402<br>Arabidopsis thaliana miR846<br>stem-loop												

CAAACATCTTGAATCCGTTGAGGTTGATC<br>ACGATGATGCCACATCCGGTTTCATTCAA<br>GGACTTCTATTCAGAACAAACTTCATGAT<br>TTCTGAACTAATTGGATATGATAAATGGT<br>AACAAGTATTCACTTGCATTCAAGGGACA<br>AAAAATCATTGGGATATATGATTATGACA<br>AACACGATTGGAAGCTGAATGGTTGCGGG<br>AGGCAAGCAGTGGGATAATGATCTGCAAG<br>TGGAAACTTCTTACTTTTATCATATCCCA<br>TCAGCTCGAAAGTCTTGATGTTAGTTTTG<br>AATTGAAGTGCTTGAATTACACCAGATTT<br>ATTGTGCTCGTGTATCCCGGTAAATTCGA<br>CTAATGACCAA	0	0	0	0	0	0	0	0	0	0	0	0
>ath-miR846 MI0005402<br>Arabidopsis thaliana miR846<br>stem-loop												
TTGAATTGAAGTGCTTGAATT	1	7	6	657	289	42	43	0	0	0	0	1
>+1 ath-miR846												
TTTGAATTGAAGTGCTTGAATT	0	21	26	849	705	60	59	0	0	0	0	0
>+2 ath-miR846												
TTTTGAATTGAAGTGCTTGAATT	0	0	0	1	1	0	0	0	0	0	0	0
>+3 ath-miR846												
GTTTTGAATTGAAGTGCTTGAATT	0	0	0	0	0	0	0	0	0	0	0	0
>ath-MIR847 MI0005410<br>Arabidopsis thaliana miR847<br>stem-loop												
CATTTTCTTGTATTCAAAGTATCTTAAGT<br>TAAGAAGAGAATAAGATTCTTACATCTTG<br>ATGAAGAGGAATGGGAAGCCGAAATCTAA<br>ACTGAAACAGAGGCGTCACCCAATTATCA<br>ATTGGTAAGTGATATCTATGTTTCGATCT<br>GTATCTCGGCCTTTCACTCCTCTTCTTCT<br>TGATGTAAGAATTTGTTATTCGACACATT<br>AGATGGAGTGAAAAATCTGTTAGATAT	0	0	0	0	0	0	0	0	0	0	0	0
>ath-miR847 MI0005410<br>Arabidopsis thaliana miR847<br>stem-loop												
TCACTCCTCTTCTTCTTGATG	0	1	1	0	0	8	3	0	0	0	0	0
>+1 ath-miR847												
TTCACTCCTCTTCTTCTTGATG	0	0	0	0	0	0	0	0	0	0	0	0
>+2 ath-miR847												
TTTCACTCCTCTTCTTCTTGATG	0	0	0	0	0	0	0	0	0	0	0	0
>+3 ath-miR847												
CTTTCACTCCTCTTCTTCTTGATG	0	0	0	0	0	0	0	0	0	0	0	0
>ath-MIR848 MI0005403<br>Arabidopsis thaliana miR848<br>stem-loop												
AAAACTTTCAGATTGCACAAGAGAATATG<br>AAAAAGTATTAACACAAGCCTTATAGCTT<br>GGCAATCTCATGTCAAATAAGGAAAAGAA<br>GAGGAACCCAAGGTATGTTTCTCATCTAC<br>CTTCTTTGACATGGGACTGCCTAAGCTAA<br>AGGCTTGCCATGTTATCACATCCTTCTTT<br>CATTTCGTCGCAACCACTTTA	0	0	0	0	0	0	0	0	0	0	0	0
>ath-miR848 MI0005403<br>Arabidopsis thaliana miR848<br>stem-loop												
TGACATGGGACTGCCTAAGCTA	24	40	31	56	72	76	43	0	0	0	0	0
>+1 ath-miR848												
TTGACATGGGACTGCCTAAGCTA	0	0	1	0	0	0	0	0	0	0	0	0
>+2 ath-miR848												
TTTGACATGGGACTGCCTAAGCTA	0	0	0	0	0	0	0	0	0	0	0	0
>+3 ath-miR848												
CTTTGACATGGGACTGCCTAAGCTA	0	0	0	0	0	0	0	0	0	0	0	0
>ath-MIR849 MI0005404<br>Arabidopsis thaliana miR849<br>stem-loop												
TAATTTGGTTAAATGTTTTCAACATTTTT<br>TATTTATTTATGATTGGACAACTACTAGA<br>CCAATGTTTAGTTAGTACATCCAATGTTA<br>TAAGTACAAAATAATATGTGTTGGATCTA<br>CTAACTAAACATTGGTGTAGTAGTTGTCC<br>GACTCTATTGTTGGGAGCGAGAAGATTGA<br>TCCCTGGTTTAATTG	0	0	0	0	0	0	0	0	0	0	0	0
>ath-miR849 MI0005404<br>Arabidopsis thaliana miR849<br>stem-loop												
TAACTAAACATTGGTGTAGTA	0	0	2	0	0	16	25	0	0	0	0	0
>+1 ath-miR849												
CTAACTAAACATTGGTGTAGTA	0	0	0	0	0	0	0	0	0	0	0	0
>+2 ath-miR849												
ACTAACTAAACATTGGTGTAGTA	0	0	0	0	0	0	0	0	0	0	0	0
>+3 ath-miR849												
TACTAACTAAACATTGGTGTAGTA	0	0	0	0	0	0	0	0	0	0	0	0
>ath-MIR850 MI0005405<br>Arabidopsis thaliana miR850<br>stem-loop												

TTTTAGATAGTGTTATGTATTTATTTCTTACCTATAGAAACCTGTCAGCTAAGATCCGGACTACAACAAAGCACGTCTACTCTACTATATTCTTTTGCGATAATCAATAGATTCTTCTAACATCGTAAAGGAAGACAAAAGAGTATACATACTTAGTTGTCGTCGTATCTTTGTTGAGAAGTTTCTAAATGGCACATAGAAAGTACATAAAACTATATGAA	0	0	0	0	0	0	0	0	0	0	0	0
>ath-miR850 MI0005405 Arabidopsis thaliana miR850 stem-loop												
TAAGATCCGGACTACAACAAAG	0	2	2	0	0	7	2	0	0	0	0	0
>+1 ath-miR850												
CTAAGATCCGGACTACAACAAAG	0	0	1	0	0	0	0	0	0	0	0	0
>+2 ath-miR850												
GCTAAGATCCGGACTACAACAAAG	0	0	0	0	0	0	0	0	0	0	0	0
>+3 ath-miR850												
AGCTAAGATCCGGACTACAACAAAG	0	0	0	0	0	0	0	0	0	0	0	0
>ath-MIR851 MI0005406 Arabidopsis thaliana miR851 stem-loop												
TTTTCTTTCTTTTCCTTTTCGTTTGATTTTGGAAAGCAAGGGACTGTCGTCTCGGTTCGCGATCCACAAGTAATCTTTTGTGGAGATTATGAAAACAATCATCACGAGAGGCTACGTGTGGGTGGCAAACAAAGACGACAATCCCTATTCCAGTTCCATCAGAACTTGAAATATCATGTGAACT	0	0	0	0	0	0	0	0	0	0	0	0
>ath-miR851-3p MI0005406 Arabidopsis thaliana miR851 stem-loop												
TGGGTGGCAAACAAAGACGAC	15	324	277	0	0	0	2	0	0	0	0	0
>+1 ath-miR851-3p												
GTGGGTGGCAAACAAAGACGAC	0	0	0	0	0	0	0	0	0	0	0	0
>+2 ath-miR851-3p												
TGTGGGTGGCAAACAAAGACGAC	0	4	4	0	0	0	0	0	0	0	0	0
>+3 ath-miR851-3p												
GTGTGGGTGGCAAACAAAGACGAC	2	0	2	0	0	0	0	0	0	0	0	0
>ath-MIR851 MI0005406 Arabidopsis thaliana miR851 stem-loop												
TTTTCTTTCTTTTCCTTTTCGTTTGATTTTGGAAAGCAAGGGACTGTCGTCTCGGTTCGCGATCCACAAGTAATCTTTTGTGGAGATTATGAAAACAATCATCACGAGAGGCTACGTGTGGGTGGCAAACAAAGACGACAATCCCTATTCCAGTTCCATCAGAACTTGAAATATCATGTGAACT	0	0	0	0	0	0	0	0	0	0	0	0
>ath-miR851-5p MI0005406 Arabidopsis thaliana miR851 stem-loop												
TCTCGGTTCGCGATCCACAAG	4	52	46	0	0	1	2	2	0	0	1	2
>+1 ath-miR851-5p												
GTCTCGGTTCGCGATCCACAAG	0	2	0	0	0	0	0	0	0	0	0	0
>+2 ath-miR851-5p												
CGTCTCGGTTCGCGATCCACAAG	0	0	0	0	0	0	0	0	0	0	0	0
>+3 ath-miR851-5p												
TCGTCTCGGTTCGCGATCCACAAG	0	0	0	0	0	0	0	0	0	0	0	0
>ath-MIR852 MI0005407 Arabidopsis thaliana miR852 stem-loop												
TTTATCCTAAACATCCTATTAAGTTTTTATGCTGAAACAAAGCTCAAAGCTCAGAGCTAAGGCGCTTATCTTCTTTGATATTGCATGGAGTATGCTTCTACTTCTCTAGGAGATGCGTTTTATGGAAATATCAAAGAAGATAAGCGCCTTAGTTCTGAAGATAAGCGCCTTATTCACAAAATGCATAAGGTTGTATTCTTTGTTAGGT	0	0	0	0	0	0	0	0	0	0	0	0
>ath-miR852 MI0005407 Arabidopsis thaliana miR852 stem-loop												
AAGATAAGCGCCTTAGTTCTG	13	67	53	9	15	21	29	0	0	0	0	0
>+1 ath-miR852												
GAAGATAAGCGCCTTAGTTCTG	0	1	2	1	4	0	0	0	0	0	0	0
>+2 ath-miR852												
AGAAGATAAGCGCCTTAGTTCTG	0	0	0	0	0	0	0	0	0	0	0	0
>+3 ath-miR852												
AAGAAGATAAGCGCCTTAGTTCTG	0	0	0	0	0	0	0	0	0	0	0	0
>ath-MIR853 MI0005408 Arabidopsis thaliana miR853 stem-loop												

GAGAGGAACAATTAGGGTTGTTTCAAACA<br>GGGTAAAGTGTTCCTGCTGCTCCCCTCTT<br>TAGCTTGGAGAAGCCAGTAAATATCTTTC<br>TCTTCAAGGAACTTGTGGGCCTCAAGAAA<br>ACCTGCTATAATTCTAGTTTTTCAACATT<br>CCTCCTTCTGATATCAGCAACAACTCCTT<br>ACCCTTAAGAATGGAGAATGGAACTTCCT<br>CAAAGATATTTACTTGGCCTCTGCAACCG<br>GAAAGGGGAGCAGCATGAAACTCTTTTAA<br>CCCTTACCCTGTATTTGTCAAAAACAGAT	0	0	0	0	0	0	0	0	0	0	0	0
>ath-miR853 MI0005408<br>Arabidopsis thaliana miR853<br>stem-loop												
TCCCCTCTTTAGCTTGGAGAAG	0	1	0	2	3	3	2	0	0	0	0	0
>+1 ath-miR853												
CTCCCCTCTTTAGCTTGGAGAAG	0	0	0	0	0	0	0	0	0	0	0	0
>+2 ath-miR853												
GCTCCCCTCTTTAGCTTGGAGAAG	0	0	0	0	0	0	0	0	0	0	0	0
>+3 ath-miR853												
TGCTCCCCTCTTTAGCTTGGAGAAG	0	0	0	0	0	0	0	0	0	0	0	0
>ath-MIR854a MI0005412<br>Arabidopsis thaliana miR854a<br>stem-loop												
CGGATGAGGATAGGGAGGAGGAGTATGAT<br>ACGAGCATGTATCATTTCAGTGAGCACGT<br>ACCTCCAGCGCGGGAGAGCAAGAGCTTGA<br>GTGAAGCTCATAGAAACAACAGTAAGTTG<br>CAGAGGTGGTGCAAGAAACAAGATAGGCT<br>ACTTATCAAGTGCTTCAAAGCCATCACGT<br>TTCTGACGGACAAGATAAGCTGCTTCTCT<br>TCTACTACAGCTATTCCG	0	0	0	0	0	0	0	0	0	0	0	0
>ath-miR854a MI0005412<br>Arabidopsis thaliana miR854a<br>stem-loop												
GATGAGGATAGGGAGGAGGAG	3	1	0	0	0	1	0	0	0	0	0	0
>+1 ath-miR854a												
GGATGAGGATAGGGAGGAGGAG	0	0	0	0	0	2	0	0	0	0	0	0
>+2 ath-miR854a												
CGGATGAGGATAGGGAGGAGGAG	4	0	0	0	0	1	5	0	0	0	0	0
>ath-MIR854b MI0005413<br>Arabidopsis thaliana miR854b<br>stem-loop												
CGGATGAGGATAGGGAGGAGGAGTATGAT<br>ACGAGCATGTATCATTTCAGTGAGCACGT<br>ACCTCCAGCGCGGGAGAGCAAGAGCTTGA<br>GTGAAGCTCACAGAAACAACAGTAAGTTG<br>CAGAGGTGGTGCAAGAAACAAGATAGGTT<br>ACTTATCAAGTGCTTCAAGGCCATCACGT<br>TTCTGACGGACAAGATAAGTTGCTTCTCT<br>TCTACCACAGCTATTCCG	0	0	0	0	0	0	0	0	0	0	0	0
>ath-miR854b MI0005413<br>Arabidopsis thaliana miR854b<br>stem-loop												
GATGAGGATAGGGAGGAGGAG	3	1	0	0	0	1	0	0	0	0	0	0
>+1 ath-miR854b												
GGATGAGGATAGGGAGGAGGAG	0	0	0	0	0	2	0	0	0	0	0	0
>+2 ath-miR854b												
CGGATGAGGATAGGGAGGAGGAG	4	0	0	0	0	1	5	0	0	0	0	0
>ath-MIR854c MI0005414<br>Arabidopsis thaliana miR854c<br>stem-loop												
CGGATGAGGATAGGGAGGAGGAGTATGAT<br>ACGAGCATGTATCATTTCAGTGAGCACGT<br>ACCTCCAGCGCGGGAGAGCAAGAGCTTGA<br>GTGAAGCTCACAGAACCAACAGTAAGTTG<br>CAGAGGTGGTGCAAGAAACAAGATAGGCT<br>ACTTATCAAGTGCTTCAAAGCCATCACGT<br>TTCTGACAGACAAGATAAGCTGCTTCTCT<br>TCTACTACAGCTATTCCG	0	0	0	0	0	0	0	0	0	0	0	0
>ath-miR854c MI0005414<br>Arabidopsis thaliana miR854c<br>stem-loop												
GATGAGGATAGGGAGGAGGAG	3	1	0	0	0	1	0	0	0	0	0	0
>+1 ath-miR854c												
GGATGAGGATAGGGAGGAGGAG	0	0	0	0	0	2	0	0	0	0	0	0
>+2 ath-miR854c												
CGGATGAGGATAGGGAGGAGGAG	4	0	0	0	0	1	5	0	0	0	0	0
>ath-MIR854d MI0005415<br>Arabidopsis thaliana miR854d<br>stem-loop												

CGGATGAGGATAGGGAGGAGGAGTATGATACGAGCATGTATCATTTCAGTGAGCACGTACCTCCAGCGCGGGAGAGCAAGAGCTTGAGTGAAGCTCACAGAAACAACAGTAAGTTGCAGAGGTGGTGCAAGAAACAAGATAGGTTACTTATCAAGTGCTTCAAGGCCATCACGTTTCTGACGGACAAGATAAGTTGCTTCTCTTCTACCACAGCTATTCCG	0	0	0	0	0	0	0	0	0	0	0	0
>ath-miR854d MI0005415 Arabidopsis thaliana miR854d stem-loop												
GATGAGGATAGGGAGGAGGAG	3	1	0	0	0	1	0	0	0	0	0	0
>+1 ath-miR854d												
GGATGAGGATAGGGAGGAGGAG	0	0	0	0	0	2	0	0	0	0	0	0
>+2 ath-miR854d												
CGGATGAGGATAGGGAGGAGGAG	4	0	0	0	0	1	5	0	0	0	0	0
>ath-MIR855 MI0005411 Arabidopsis thaliana miR855 stem-loop												
TTTTGGAGCAAAAGCTAAGGAAAAGGAAGATAAGGACGATTTTGGTTCTGGAATTAGCGAGGAGTGTCGATCGACACACTCTTGGTGTCGATCGACACCAGTTTCGGCCCAGCAAAAACCCTAGACGCTTTCGAGTTTACAAGAGCTCAAGTTTGCCCTAGAAGATTTCATTATTTGCATTATTAGTCCGTGGACGTTTTTAGGGTCTATATATATTGTTTTTAGACCTAAGTTTCTTTTATCAAGTCTTTTATCAAGCTTTTGCAAAACCA	0	0	0	0	0	0	0	0	0	0	0	0
>ath-miR855 MI0005411 Arabidopsis thaliana miR855 stem-loop												
AGCAAAAGCTAAGGAAAAGGAA	0	0	0	0	0	0	0	0	0	0	0	0
>+1 ath-miR855												
GAGCAAAAGCTAAGGAAAAGGAA	0	0	0	0	0	0	0	0	0	0	0	0
>+2 ath-miR855												
GGAGCAAAAGCTAAGGAAAAGGAA	0	0	0	0	1	0	0	0	0	0	0	0
>+3 ath-miR855												
TGGAGCAAAAGCTAAGGAAAAGGAA	0	0	0	0	0	0	0	0	0	0	0	0
>ath-MIR856 MI0005433 Arabidopsis thaliana miR856 stem-loop												
ATGTATAAGAGGTGAGACTGTTGGCTTTAATCCTACCAATAACTTCAGCTGCATCATGAAGAGTAGACAGAGATTCTGGCGGTTGAGGGTTTCTTCGTAGAGGTTGATCGATTCTTAATGTCCTGTGGATTTTAAGTTCCCAAAACTGTTGCTTAGGACATACAAGATCGTTCAATGTCCAAGAAGAAACCTGAGCCGCTACAATCATCGGTCTAATCTCTTTAGCAGATGAAGTTATTTGTGGTATAAAACCCACAGTTTTATCATGCAT	0	0	0	0	0	0	0	0	0	0	0	0
>ath-miR856 MI0005433 Arabidopsis thaliana miR856 stem-loop												
TAATCCTACCAATAACTTCAGC	2	9	8	0	0	1	0	0	0	0	0	0
>+1 ath-miR856												
TTAATCCTACCAATAACTTCAGC	0	0	0	0	0	0	0	0	0	0	0	0
>+2 ath-miR856												
TTTAATCCTACCAATAACTTCAGC	0	0	0	0	0	0	0	0	0	0	0	0
>+3 ath-miR856												
CTTTAATCCTACCAATAACTTCAGC	0	0	0	0	0	0	0	0	0	0	0	0
>ath-MIR857 MI0005434 Arabidopsis thaliana miR857 stem-loop												
TTCGACTCCTACAACAAACTTTCACCATACAAAATAATGAAGAAAACACCCAAAAAGGTTTGAATGTGTGAGGTTAGTCTCATTTTTAAAATAGTGACTTGTTTTTTTTAAAATGTTTTTTAAGAATGTTTTTTCAGCTTTTTATTTTATTTTAAACCTAAAGGTAGCGTGACTATTGTGGAGAAATAAAGAAACATAAGAAAAAATATTTTTGGTTTATAGTAAGATGTTGTTTTAAAATATATATTTAAAAACAATGTCACTATTTTAAACGGAATATAGACATTTTCTATTAATGAGATTAACGTCGAGCATTTGAATTTCTAGATTTTTTTCTAAGTTATTTTGTATGTTGAAGGTGTATTGTAGGAATCGGA	0	0	0	0	0	0	0	0	0	0	0	0
>ath-miR857 MI0005434 Arabidopsis thaliana miR857 stem-loop												
TTTTGTATGTTGAAGGTGTAT	1	59	50	1	5	0	1	0	0	0	0	0
>+1 ath-miR857												
ATTTTGTATGTTGAAGGTGTAT	0	3	3	0	0	0	0	0	0	0	0	0
>+2 ath-miR857												
TATTTTGTATGTTGAAGGTGTAT	0	0	0	0	0	0	0	0	0	0	0	0
>+3 ath-miR857												

TTATTTTGTATGTTGAAGGTGTAT	0	0	1	0	0	0	0	0	0	0	0	0
>ath-MIR858 MI0005435 Arabidopsis thaliana miR858 stem-loop												
CTCATCTTTGTTTCGTTGTCTGTTCGACCTTGGTCTCGATCTCTCCTCAAAACCCTAATACGCGCTTTATCGTTTATTTCATTCATCCATCCTTCTTATGATCATCATAAACTAACGTACATGGTTTATCGGTTTGTTATTGATGGGGATTGGGGTAGATAGACGATCGAAACTTTTGATTTCTGAG	0	0	0	0	0	0	0	0	0	0	0	0
>ath-miR858 MI0005435 Arabidopsis thaliana miR858 stem-loop												
TTTCGTTGTCTGTTCGACCTT	2	13	12	0	2	12	9	20	0	10	6	48
>+1 ath-miR858												
GTTTCGTTGTCTGTTCGACCTT	0	0	0	0	0	0	0	0	0	0	0	0
>+2 ath-miR858												
TGTTTCGTTGTCTGTTCGACCTT	0	0	0	0	0	0	0	0	0	0	0	0
>+3 ath-miR858												
TTGTTTCGTTGTCTGTTCGACCTT	0	0	0	0	0	0	0	0	0	0	0	0
>ath-MIR859 MI0005436 Arabidopsis thaliana miR859 stem-loop												
AAAGGTAGATGTCGAAATCTCTCTGTTGTGAAGTCAAACATGAGTATGAATTAACATTAATGGATCTTTTTGATTCATAATACTCATGTTTGGTTTCACAGTAGATAGATATCGACGTCTATCTTT	0	0	0	0	0	0	0	0	0	0	0	0
>ath-miR859 MI0005436 Arabidopsis thaliana miR859 stem-loop												
TCTCTCTGTTGTGAAGTCAAA	4	24	19	4	1	0	0	1	0	0	0	0
>+1 ath-miR859												
ATCTCTCTGTTGTGAAGTCAAA	0	0	0	0	0	0	0	0	0	0	0	0
>+2 ath-miR859												
AATCTCTCTGTTGTGAAGTCAAA	0	0	0	0	0	0	0	0	0	0	0	0
>+3 ath-miR859												
AAATCTCTCTGTTGTGAAGTCAAA	0	0	0	0	0	0	0	0	0	0	0	0
>ath-MIR860 MI0005437 Arabidopsis thaliana miR860 stem-loop												
ATGTCTTGGTTAAATTCAATATATATAGTCCAATCTATTGAAGTACTAGTACACCAGCTCTAATAAGCTGATGTGGGTAAGTAGTTCAATAGATTGGACTATGTATATTAAATTTGACTAAGAGAT	0	0	0	0	0	0	0	0	0	0	0	0
>ath-miR860 MI0005437 Arabidopsis thaliana miR860 stem-loop												
TCAATAGATTGGACTATGTAT	34	52	51	62	114	22	38	1	0	0	0	0
>+1 ath-miR860												
TTCAATAGATTGGACTATGTAT	0	0	0	0	0	0	0	0	0	0	0	0
>+2 ath-miR860												
GTTCAATAGATTGGACTATGTAT	0	0	0	0	0	0	0	0	0	0	0	0
>+3 ath-miR860												
AGTTCAATAGATTGGACTATGTAT	0	0	0	0	0	0	0	0	0	0	0	0
>ath-MIR861 MI0005438 Arabidopsis thaliana miR861 stem-loop												
GTATCATTGTTTTCGAACGATGTCCTTGGAGAAATATGCGTCAATGGAGTCCGTCTTCTTGAGAAGCATTTTTGAGTGAGAATGATTCTCATGATGGATATGTCTTCAAGGACTTCTTTCAAACAATGGTAC	0	0	0	0	0	0	0	0	0	0	0	0
>ath-miR861-3p MI0005438 Arabidopsis thaliana miR861 stem-loop												
GATGGATATGTCTTCAAGGAC	0	1	0	1	0	2	4	0	0	0	0	0
>+1 ath-miR861-3p												
TGATGGATATGTCTTCAAGGAC	0	0	0	0	0	0	0	0	0	0	0	0
>+2 ath-miR861-3p												
ATGATGGATATGTCTTCAAGGAC	0	0	0	0	0	0	0	0	0	0	0	0
>+3 ath-miR861-3p												
CATGATGGATATGTCTTCAAGGAC	0	0	0	0	0	0	0	0	0	0	0	0
>ath-MIR861 MI0005438 Arabidopsis thaliana miR861 stem-loop												
GTATCATTGTTTTCGAACGATGTCCTTGGAGAAATATGCGTCAATGGAGTCCGTCTTCTTGAGAAGCATTTTTGAGTGAGAATGATTCTCATGATGGATATGTCTTCAAGGACTTCTTTCAAACAATGGTAC	0	0	0	0	0	0	0	0	0	0	0	0
>ath-miR861-5p MI0005438 Arabidopsis thaliana miR861 stem-loop												
CCTTGGAGAAATATGCGTCAA	2	16	11	0	0	1	4	0	0	0	0	0
>+1 ath-miR861-5p												

TCCTTGGAGAAATATGCGTCAA	1	1	1	0	0	2	0	0	0	0	0	0
>+2 ath-miR861-5p												
GTCCTTGGAGAAATATGCGTCAA	0	0	0	0	0	0	0	0	0	0	0	0
>+3 ath-miR861-5p												
TGTCCTTGGAGAAATATGCGTCAA	0	0	0	0	0	0	0	0	0	0	0	0
>ath-MIR862 MI0005439<br>Arabidopsis thaliana miR862<br>stem-loop												
ACAGTAGTTTTCCAATAGGTCGAGCATGT<br>GCTGAAACTGTGTGTACAGAGTTCTCATA<br>AACACACAGTTTCAGCATATGCTGGATCT<br>ACTTGAAGACTACTGT	0	0	0	0	0	0	0	0	0	0	0	0
>ath-miR862-3p MI0005439<br>Arabidopsis thaliana miR862<br>stem-loop												
ATATGCTGGATCTACTTGAAG	0	2	2	0	1	0	0	0	0	0	0	0
>+1 ath-miR862-3p												
CATATGCTGGATCTACTTGAAG	0	0	1	0	0	0	0	0	0	0	0	0
>+2 ath-miR862-3p												
GCATATGCTGGATCTACTTGAAG	0	0	0	0	0	0	0	0	0	0	0	0
>+3 ath-miR862-3p												
AGCATATGCTGGATCTACTTGAAG	0	0	0	0	0	0	0	0	0	0	0	0
>ath-MIR862 MI0005439<br>Arabidopsis thaliana miR862<br>stem-loop												
ACAGTAGTTTTCCAATAGGTCGAGCATGT<br>GCTGAAACTGTGTGTACAGAGTTCTCATA<br>AACACACAGTTTCAGCATATGCTGGATCT<br>ACTTGAAGACTACTGT	0	0	0	0	0	0	0	0	0	0	0	0
>ath-miR862-5p MI0005439<br>Arabidopsis thaliana miR862<br>stem-loop												
TCCAATAGGTCGAGCATGTGC	0	17	13	3	2	3	1	0	0	0	0	0
>+1 ath-miR862-5p												
TTCCAATAGGTCGAGCATGTGC	0	1	1	0	0	0	0	0	0	0	0	0
>+2 ath-miR862-5p												
TTTCCAATAGGTCGAGCATGTGC	0	0	0	0	0	0	0	0	0	0	0	0
>+3 ath-miR862-5p												
TTTTCCAATAGGTCGAGCATGTGC	0	0	0	0	0	0	0	0	0	0	0	0
>ath-MIR863 MI0005440<br>Arabidopsis thaliana miR863<br>stem-loop												
CTCATAAGATGTAGAAACTAGAGAAGAGT<br>TTGGGGGAAAACTCTTTCTTATGTCTTGT<br>TGATCTCAATAGCATTGATACAATGTATA<br>TATATGCAATTTAGAATATGTTTTTCCTA<br>ATTTGAATAAGAAATAGATAAACATGAAT<br>AAGTATATATATATTTCCTATTTAGAATA<br>TTTATTTATTCAAATTAGGTGAAACATAA<br>TCTAGGTTGTATATATATATGTTATACCA<br>ATGCGATTGAGAGCAACAAGACATAATAA<br>AGAGTTTTCGAAAACTTTTTTCTAGTTTC<br>TACATAAGAGGAG	0	0	0	0	0	0	0	0	0	0	0	0
>ath-miR863-3p MI0005440<br>Arabidopsis thaliana miR863<br>stem-loop												
TTGAGAGCAACAAGACATAAT	4	17	15	2	1	87	82	0	0	0	0	0
>+1 ath-miR863-3p												
ATTGAGAGCAACAAGACATAAT	0	0	0	0	0	0	1	0	0	0	0	0
>+2 ath-miR863-3p												
GATTGAGAGCAACAAGACATAAT	0	0	0	0	0	1	0	0	0	0	0	0
>+3 ath-miR863-3p												
CGATTGAGAGCAACAAGACATAAT	0	0	0	0	0	0	0	0	0	0	0	0
>ath-MIR863 MI0005440<br>Arabidopsis thaliana miR863<br>stem-loop												
CTCATAAGATGTAGAAACTAGAGAAGAGT<br>TTGGGGGAAAACTCTTTCTTATGTCTTGT<br>TGATCTCAATAGCATTGATACAATGTATA<br>TATATGCAATTTAGAATATGTTTTTCCTA<br>ATTTGAATAAGAAATAGATAAACATGAAT<br>AAGTATATATATATTTCCTATTTAGAATA<br>TTTATTTATTCAAATTAGGTGAAACATAA<br>TCTAGGTTGTATATATATATGTTATACCA<br>ATGCGATTGAGAGCAACAAGACATAATAA<br>AGAGTTTTCGAAAACTTTTTTCTAGTTTC<br>TACATAAGAGGAG	0	0	0	0	0	0	0	0	0	0	0	0
>ath-miR863-5p MI0005440<br>Arabidopsis thaliana miR863<br>stem-loop												
TTATGTCTTGTTGATCTCAAT	0	0	0	0	0	0	0	0	0	0	0	0
>+1 ath-miR863-5p												
CTTATGTCTTGTTGATCTCAAT	0	0	0	0	0	0	0	0	0	0	0	0
>+2 ath-miR863-5p												
TCTTATGTCTTGTTGATCTCAAT	0	0	0	0	0	0	1	0	0	0	0	0
>+3 ath-miR863-5p												
TTCTTATGTCTTGTTGATCTCAAT	0	0	0	0	0	0	0	0	0	0	0	0

>ath-MIR864 MI0005441 Arabidopsis thaliana miR864 stem-loop												
GCTTCAGGTATGATTGACTTCAAAAAATACCTTGAAACTATAAACCTCAGTTTCTTTGAATTTGATTTTTAAAGTCAATAATACCTTGAAGC	0	0	0	0	0	0	0	0	0	0	0	0
>ath-miR864-3p MI0005441 Arabidopsis thaliana miR864 stem-loop												
TAAAGTCAATAATACCTTGAAG	0	1	0	0	0	0	3	0	0	0	0	0
>+1 ath-miR864-3p												
TTAAAGTCAATAATACCTTGAAG	0	1	1	0	0	1	1	0	0	0	0	0
>+2 ath-miR864-3p												
TTTAAAGTCAATAATACCTTGAAG	0	0	0	0	0	0	1	0	0	0	0	0
>+3 ath-miR864-3p												
TTTTAAAGTCAATAATACCTTGAAG	0	0	0	0	0	0	0	0	0	0	0	0
>ath-MIR864 MI0005441 Arabidopsis thaliana miR864 stem-loop												
GCTTCAGGTATGATTGACTTCAAAAAATACCTTGAAACTATAAACCTCAGTTTCTTTGAATTTGATTTTTAAAGTCAATAATACCTTGAAGC	0	0	0	0	0	0	0	0	0	0	0	0
>ath-miR864-5p MI0005441 Arabidopsis thaliana miR864 stem-loop												
TCAGGTATGATTGACTTCAAA	7	51	53	8	11	27	31	0	0	0	0	0
>+1 ath-miR864-5p												
TTCAGGTATGATTGACTTCAAA	0	1	2	1	0	0	1	0	0	0	0	0
>+2 ath-miR864-5p												
CTTCAGGTATGATTGACTTCAAA	0	0	0	0	0	0	0	0	0	0	0	0
>+3 ath-miR864-5p												
GCTTCAGGTATGATTGACTTCAAA	0	0	0	0	0	0	0	0	0	0	0	0
>ath-MIR865 MI0005442 Arabidopsis thaliana miR865 stem-loop												
GATCTGGGATGAATTTGGATCTAATTGAGCAAAAAATTGTGTTTTTTCAATCTATTGAATTTCACATCCTTAAACCCTTGCATATTCAATCTATTGAATTTCGCATATTTTTCCTCAAATTTATCCAAAATCATCCCAAATC	0	0	0	0	0	0	0	0	0	0	0	0
>ath-miR865-3p MI0005442 Arabidopsis thaliana miR865 stem-loop												
TTTTTCCTCAAATTTATCCAA	0	0	0	0	0	0	1	0	0	0	0	0
>+1 ath-miR865-3p												
ATTTTTCCTCAAATTTATCCAA	0	0	0	0	0	0	0	0	0	0	0	0
>+2 ath-miR865-3p												
TATTTTTCCTCAAATTTATCCAA	0	0	0	0	0	0	0	0	0	0	0	0
>+3 ath-miR865-3p												
ATATTTTTCCTCAAATTTATCCAA	0	0	0	0	0	0	0	0	0	0	0	0
>ath-MIR865 MI0005442 Arabidopsis thaliana miR865 stem-loop												
GATCTGGGATGAATTTGGATCTAATTGAGCAAAAAATTGTGTTTTTTCAATCTATTGAATTTCACATCCTTAAACCCTTGCATATTCAATCTATTGAATTTCGCATATTTTTCCTCAAATTTATCCAAAATCATCCCAAATC	0	0	0	0	0	0	0	0	0	0	0	0
>ath-miR865-5p MI0005442 Arabidopsis thaliana miR865 stem-loop												
ATGAATTTGGATCTAATTGAG	0	0	1	0	0	0	0	0	0	0	0	0
>+1 ath-miR865-5p												
GATGAATTTGGATCTAATTGAG	0	0	0	0	0	0	0	0	0	0	0	0
>+2 ath-miR865-5p												
GGATGAATTTGGATCTAATTGAG	0	0	0	0	0	0	0	0	0	0	0	0
>+3 ath-miR865-5p												
GGGATGAATTTGGATCTAATTGAG	0	0	0	0	0	0	0	0	0	0	0	0
>ath-MIR866 MI0005443 Arabidopsis thaliana miR866 stem-loop												
TCATCTATTCTTACTTTTCAAGGAACGGATTTTGTTAACTAAATGATTCACACCCTTGAAGAAACACGATCTCGTGTGAATCATTTCGTTAACAAAATCCGTCTTTGAAGAGTAAGTATATTGTGA	0	0	0	0	0	0	0	0	0	0	0	0
>ath-miR866-3p MI0005443 Arabidopsis thaliana miR866 stem-loop												
ACAAAATCCGTCTTTGAAGA	0	2	2	1	0	0	2	0	0	0	0	0
>+1 ath-miR866-3p												
AACAAAATCCGTCTTTGAAGA	0	0	0	1	0	0	0	0	0	0	0	0
>+2 ath-miR866-3p												
TAACAAAATCCGTCTTTGAAGA	0	0	0	0	1	0	1	0	0	0	0	0

>+3 ath-miR866-3p												
TTAACAAAATCCGTCTTTGAAGA	0	0	0	0	0	0	0	0	0	0	0	0
>ath-MIR866 MI0005443 Arabidopsis thaliana miR866 stem-loop												
TCATCTATTCTTACTTTTCAAGGAACGGATTTTGTTAACTAAATGATTCACACCCTTGAAGAAACACGATCTCGTGTGAATCATTTCGTTAACAAAATCCGTCTTTGAAGAGTAAGTATATTGTGA	0	0	0	0	0	0	0	0	0	0	0	0
>ath-miR866-5p MI0005443 Arabidopsis thaliana miR866 stem-loop												
TCAAGGAACGGATTTTGTTAA	12	38	46	18	25	23	25	0	0	0	0	0
>+1 ath-miR866-5p												
TTCAAGGAACGGATTTTGTTAA	6	9	20	7	7	9	11	0	0	0	0	0
>+2 ath-miR866-5p												
TTTCAAGGAACGGATTTTGTTAA	0	0	0	0	0	0	0	0	0	0	0	0
>+3 ath-miR866-5p												
TTTTCAAGGAACGGATTTTGTTAA	0	0	0	0	0	0	0	0	0	0	0	0
>ath-MIR867 MI0005445 Arabidopsis thaliana miR867 stem-loop												
ACACATAAAACTAGTATATTGCCTTGAACATGGTTTATTAGGAAAGACATGAGAACATATCTGAACATAGTTTTTCCTTATATACAATGTTCAAAGTAATGAATTAGTTTTATTTTGT	0	0	0	0	0	0	0	0	0	0	0	0
>ath-miR867 MI0005445 Arabidopsis thaliana miR867 stem-loop												
TTGAACATGGTTTATTAGGAA	13	29	26	0	0	0	0	0	0	0	0	0
>+1 ath-miR867												
CTTGAACATGGTTTATTAGGAA	0	0	0	0	0	0	0	0	0	0	0	0
>+2 ath-miR867												
CCTTGAACATGGTTTATTAGGAA	0	0	0	0	0	0	0	0	0	0	0	0
>+3 ath-miR867												
GCCTTGAACATGGTTTATTAGGAA	0	0	0	0	0	0	0	0	0	0	0	0
>ath-MIR868 MI0005446 Arabidopsis thaliana miR868 stem-loop												
GAATAAGCATTGTCGGCACTTGAGAATGTAAAGTGATGATCAGCTATCGTATTTCATGTCGTAATAGTAGTCACGTTAGGGTGATATAACGAGGCGAGAGTTATATCATCATAGTGCGATTTCTATTATGACATAAAATACGTTTTGGATTTTAAAACACTTGACCTTCTTAAGTGCTGATAATGCTTCTTTC	0	0	0	0	0	0	0	0	0	0	0	0
>ath-miR868 MI0005446 Arabidopsis thaliana miR868 stem-loop												
CTTCTTAAGTGCTGATAATGC	0	0	0	0	0	0	0	0	0	0	0	0
>+1 ath-miR868												
CCTTCTTAAGTGCTGATAATGC	0	0	0	0	0	0	0	0	0	0	0	0
>+2 ath-miR868												
ACCTTCTTAAGTGCTGATAATGC	0	0	0	0	0	0	0	0	0	0	0	0
>+3 ath-miR868												
GACCTTCTTAAGTGCTGATAATGC	0	0	0	0	0	0	0	0	0	0	0	0
>ath-MIR869 MI0005447 Arabidopsis thaliana miR869 stem-loop												
GCCCACCATACAGATTTCCATACGGTAGAAGAAAGTATCACCTATCTCAACTCCAGGATTGGACCAGTACTAGGCTCATCATAGTTAAGCCACAATCCACGATTCCTCATCAGCTATAAAAGCAAAACCTTCATATCTATCCTTGTTTCTAAACCAGTCTCATAGATATGAAGGCTTTGTTTCTGATGAAGGATTGTGGGATTGTTGCTTAACACTGATGAGCATATTATTGGTTCAATTCTGGTGTTGAGATAGTTGACACTTCTTATACCGTATGAAAACATGTGTGGTGGGC	0	0	0	0	0	0	0	0	0	0	0	0
>ath-miR869.1 MI0005447 Arabidopsis thaliana miR869 stem-loop												
ATTGGTTCAATTCTGGTGTTG	0	0	0	1	2	0	0	0	0	0	0	0
>+1 ath-miR869.1												
TATTGGTTCAATTCTGGTGTTG	0	0	1	2	2	0	0	0	0	0	0	0
>+2 ath-miR869.1												
TTATTGGTTCAATTCTGGTGTTG	0	0	0	0	0	0	0	0	0	0	0	0
>+3 ath-miR869.1												
ATTATTGGTTCAATTCTGGTGTTG	0	0	0	0	0	0	0	0	0	0	0	0
>ath-MIR869 MI0005447 Arabidopsis thaliana miR869 stem-loop												

GCCCACCATACAGATTTCCATACGGTAGA AGAAAGTATCACCTATCTCAACTCCAGGA TTGGACCAGTACTAGGCTCATCATAGTTA AGCCACAATCCACGATTCCTCATCAGCTA TAAAAGCAAAACCTTCATATCTATCCTTG TTTCTAAACCAGTCTCATAGATATGAAGG CTTTGTTTCTGATGAAGGATTGTGGGATT GTTGCTTAACACTGATGAGCATATTATTG GTTCAATTCTGGTGTTGAGATAGTTGACA CTTCTTATACCGTATGAAAACATGTGTGG TGGGC	0	0	0	0	0	0	0	0	0	0	0	0
>ath-miR869.2 MI0005447 Arabidopsis thaliana miR869 stem-loop												
TCTGGTGTTGAGATAGTTGAC	43	356	331	635	669	39	42	0	0	0	0	0
>+1 ath-miR869.2	0.0%	3.1%	2.1%	0.9%	2.1%	2.6%	0.0%	#####	#####	#####	#DIV/0!	#####
TTCTGGTGTTGAGATAGTTGAC	0	11	7	6	14	1	0	0	0	0	0	0
>+2 ath-miR869.2												
ATTCTGGTGTTGAGATAGTTGAC	0	0	0	0	0	0	0	0	0	0	0	0
>+3 ath-miR869.2												
AATTCTGGTGTTGAGATAGTTGAC	0	0	0	0	0	0	0	0	0	0	0	0
>ath-MIR870 MI0005448 Arabidopsis thaliana miR870 stem-loop												
GATCGAAGAACATCAAATTAGAATGTGAT GCAAAACTTAGACTCCTAACATTTGCAAC ACAATCTAATTTGGTGTTTCTTCGATC	0	0	0	0	0	0	0	0	0	0	0	0
>ath-miR870 MI0005448 Arabidopsis thaliana miR870 stem-loop												
TAATTTGGTGTTTCTTCGATC	0	1	0	0	0	0	0	0	0	0	0	0
>+1 ath-miR870												
CTAATTTGGTGTTTCTTCGATC	0	0	0	0	0	0	0	0	0	0	0	0
>+2 ath-miR870												
TCTAATTTGGTGTTTCTTCGATC	0	0	0	0	0	0	0	0	0	0	0	0
>+3 ath-miR870												
ATCTAATTTGGTGTTTCTTCGATC	0	0	0	0	0	0	0	0	0	0	0	0
