# Supplementary material for: Naturally occurring variations in sequence length creates microRNA isoforms that differ in argonaute effector complex specificity
Source: Silence. 2010 Jun 9;1:12. doi: 10.1186/1758-907X-1-12 (PMC2901367; doi:10.1186/1758-907X-1-12)
Supplement: Additional file 2 — Validated miRNA isoforms and their AGO association. All validated miRNA isoforms from Additional file 1 were scanned across various argonaute complexes. [file 1758-907X-1-12-S2.PDF]

Additional File 2

Naturally Occurring Variations in Sequence Length Creates MiRNA Isoforms that Differ in Argonaute Effector Complex Specificity.

H. Alexander Ebhardt, Amber Fedynak, Richard P. Fahlman

In Silico Northern analysis to determine the frequency that all A. thaliana miRNAs and their +1, +2 and +3 isoforms are observed in four different argonaute complexes.

For each miRNA the pre-miRNA hairpin as well as the mature miRNA and length isoforms of are given. Values list the number of occurances each sequence exists in a given dataset. Sequences with no observed occurances are omitted from the list.

	Sequences isolated With:			
	AGO1	AGO2	AGO4	AGO5
**>ath-miR157d MI0000187 Arabidopsis thaliana miR157d stem-loop**				
**TGACAGAAGATAGAGAGCAC**	325	1	9	4
**>+1 ath-miR157d**				
**CTGACAGAAGATAGAGAGCAC**	118	0	3	54
**>ath-miR156h MI0001083 Arabidopsis thaliana miR156h stem-loop**				
**TGACAGAAGAAAGAGAGCAC**	7	0	2	4
**>+1 ath-miR156h**				
**TTGACAGAAGAAAGAGAGCAC**	14	1	13	148
**>ath-miR156d MI0000181 Arabidopsis thaliana miR156d stem-loop**				
**TGACAGAAGAGAGTGAGCAC**	3007	1	62	8
**>+1 ath-miR156d**				
**TTGACAGAAGAGAGTGAGCAC**	1881	2	28	4
**>ath-miR158a MI0000188 Arabidopsis thaliana miR158a stem-loop**				
**TCCCAAATGTAGACAAAGCA**	14422	29	102	167
**>+1 ath-miR158a**				
**TTCCCAAATGTAGACAAAGCA**	861	1	2	9
**>ath-miR158b MI0001084 Arabidopsis thaliana miR158b stem-loop**				
**CCCCAAATGTAGACAAAGCA**	91	1	1	4
**>+1 ath-miR158b**				
**TCCCCAAATGTAGACAAAGCA**	52	0	0	0
**>ath-miR160a MI0000190 Arabidopsis thaliana miR160a stem-loop**				

TGCCTGGCTCCCTGTATGCCA	7771	41	6	9
>+1 ath-miR160a				
ATGCCTGGCTCCCTGTATGCCA	229	223	1	0

>ath-miR160c MI0000192 Arabidopsis thaliana miR160c stem-loop				
TGCCTGGCTCCCTGTATGCCA	7771	41	6	9
>+1 ath-miR160c				
ATGCCTGGCTCCCTGTATGCCA	229	223	1	0

>ath-miR161.1 MI0000193 Arabidopsis thaliana miR161 stem-loop				
TGAAAGTGACTACATCGGGGT	122349	13	88	142
>+1 ath-miR161.1				
TTGAAAGTGACTACATCGGGGT	39366	71	100	154

>ath-miR162a MI0000194 Arabidopsis thaliana miR162a stem-loop				
TCGATAAACCTCTGCATCCAG	3314	3	12	13
>+1 ath-miR162a				
ATCGATAAACCTCTGCATCCAG	52	29	0	2

>ath-miR162b MI0000195 Arabidopsis thaliana miR162b stem-loop				
TCGATAAACCTCTGCATCCAG	3314	3	12	13
>+1 ath-miR162b				
ATCGATAAACCTCTGCATCCAG	52	29	0	2

>ath-miR164b MI0000198 Arabidopsis thaliana miR164b stem-loop				
TGGAGAAGCAGGGCACGTGCA	388368	13	396	11
>+1 ath-miR164b				
ATGGAGAAGCAGGGCACGTGCA	1285	0	0	0

>ath-miR164c MI0001087 Arabidopsis thaliana miR164c stem-loop				
TGGAGAAGCAGGGCACGTGCG	388368	13	396	11
>+1 ath-miR164c				
ATGGAGAAGCAGGGCACGTGCG	1285	0	0	0

>ath-miR166a MI0000201 Arabidopsis thaliana miR166a stem-loop				
TCGGACCAGGCTTCATTCCCC	4427	21	52	45
>+1 ath-miR166a				
TTCGGACCAGGCTTCATTCCCC	126	1	15	91

>ath-miR166b MI0000202 Arabidopsis thaliana miR166b stem-loop				
TCGGACCAGGCTTCATTCCCC	4427	21	52	45
>+1 ath-miR166b				
GTCGGACCAGGCTTCATTCCCC	62	0	2	21

>ath-miR166c MI0000203 Arabidopsis thaliana miR166c stem-loop				
TCGGACCAGGCTTCATTCCCC	4427	21	52	45
>+1 ath-miR166c				
CTCGGACCAGGCTTCATTCCCC	82	0	0	10

>ath-miR166d MI0000204 Arabidopsis thaliana miR166d stem-loop				
TCGGACCAGGCTTCATTCCCC	4427	21	52	45
>+1 ath-miR166d				
CTCGGACCAGGCTTCATTCCCC	82	0	0	10

>ath-miR166e MI0000205 Arabidopsis thaliana miR166e stem-loop				
TCGGACCAGGCTTCATTCCCC	4427	21	52	45
>+1 ath-miR166e				
GTCGGACCAGGCTTCATTCCCC	62	0	2	21

>ath-miR166f MI0000206 Arabidopsis thaliana miR166f stem-loop				
TCGGACCAGGCTTCATTCCCC	4427	21	52	45
>+1 ath-miR166f				
GTCGGACCAGGCTTCATTCCCC	62	0	2	21

>ath-miR166g MI0000207 Arabidopsis thaliana miR166g stem-loop				
TCGGACCAGGCTTCATTCCCC	4427	21	52	45
>+1 ath-miR166g				
CTCGGACCAGGCTTCATTCCCC	82	0	0	10

>ath-miR167a MI0000208 Arabidopsis thaliana miR167a stem-loop				
TGAAGCTGCCAGCATGATCTA	365764	210	1152	1160
>+1 ath-miR167a				
ATGAAGCTGCCAGCATGATCTA	623	0	0	3

>ath-miR167c MI0001088 Arabidopsis thaliana miR167c stem-loop				
TAAGCTGCCAGCATGATCTTG	93	0	21	9
>+1 ath-miR167c				

TTAAGCTGCCAGCATGATCTTG	6	0	0	1
>ath-miR167d MI0000975 Arabidopsis thaliana miR167d stem-loop				
TGAAGCTGCCAGCATGATCTGG	2727	55	347	21
>+1 ath-miR167d				
CTGAAGCTGCCAGCATGATCTGG	15	0	0	0
>ath-miR168a MI0000210 Arabidopsis thaliana miR168a stem-loop				
TCGCTTGGTGCAGGTCGGGAA	85948	60	571	55
>+1 ath-miR168a				
TTCGCTTGGTGCAGGTCGGGAA	29	0	5	0
>ath-miR168b MI0000211 Arabidopsis thaliana miR168b stem-loop				
TCGCTTGGTGCAGGTCGGGAA	85948	60	571	55
>+1 ath-miR168b				
TTCGCTTGGTGCAGGTCGGGAA	29	0	5	0
>ath-miR172a MI0000215 Arabidopsis thaliana miR172a stem-loop				
AGAATCTTGATGATGCTGCAT	9796	470	1793	717
>+1 ath-miR172a				
GAGAATCTTGATGATGCTGCAT	31	0	45	3
>ath-miR172b MI0000216 Arabidopsis thaliana miR172b stem-loop				
AGAATCTTGATGATGCTGCAT	9796	470	1793	717
>+1 ath-miR172b				
GAGAATCTTGATGATGCTGCAT	31	0	45	3
>ath-miR172c MI0000991 Arabidopsis thaliana miR172c stem-loop				
AGAATCTTGATGATGCTGCAG	9796	470	1793	717
>+1 ath-miR172c				
GAGAATCTTGATGATGCTGCAG	31	0	45	3
>ath-miR172d MI0000992 Arabidopsis thaliana miR172d stem-loop				
AGAATCTTGATGATGCTGCAG	9796	470	1793	717
>+1 ath-miR172d				
GAGAATCTTGATGATGCTGCAG	31	0	45	3

>ath-miR319a MI0000544 Arabidopsis thaliana miR319a stem-loop				
TTGGACTGAAGGGAGCTCCCT	856	112	195	6
>+1 ath-miR319a				
ATTGGACTGAAGGGAGCTCCCT	10	166	7	0
>+2 ath-miR319a				
GATTGGACTGAAGGGAGCTCCCT	137	1	9	0

>ath-miR319b MI0000545 Arabidopsis thaliana miR319b stem-loop				
TTGGACTGAAGGGAGCTCCCT	856	112	195	6
>+1 ath-miR319b				
CTTGGACTGAAGGGAGCTCCCT	37	0	4	1

>ath-miR319c MI0001086 Arabidopsis thaliana miR319c stem-loop				
TTGGACTGAAGGGAGCTCCTT	27	6	35	7
>+1 ath-miR319c				
TTTGGACTGAAGGGAGCTCCTT	66	0	13	1

>ath-miR390a MI0001000 Arabidopsis thaliana miR390a stem-loop				
AAGCTCAGGAGGGATAGCGCC	343	5934	1921	11
>+1 ath-miR390a				
AAAGCTCAGGAGGGATAGCGCC	9	51	66	0

>ath-miR390b MI0001001 Arabidopsis thaliana miR390b stem-loop				
AAGCTCAGGAGGGATAGCGCC	343	5934	1921	11
>+1 ath-miR390b				
AAAGCTCAGGAGGGATAGCGCC	9	51	66	0

>ath-miR396a MI0001013 Arabidopsis thaliana miR396a stem-loop				
TTCCACAGCTTTCTTGAACTG	2334	36	115	54
>+1 ath-miR396a				
CTTCCACAGCTTTCTTGAACTG	254	1	0	2

>ath-miR398a MI0001017 Arabidopsis thaliana miR398a stem-loop				
TGTGTTCTCAGGTCACCCCTT	111885	12	43	245
>+1 ath-miR398a				
TTGTGTTCTCAGGTCACCCCTT	796	9	0	0
>+2 ath-miR398a				
TTTGTGTTCTCAGGTCACCCCTT	32	0	0	0

>ath-miR398b MI0001018 Arabidopsis thaliana miR398b stem-loop				
TGTGTTCTCAGGTCACCCCTG	111885	12	43	245
>+1 ath-miR398b				
ATGTGTTCTCAGGTCACCCCTG	152	8	3	1

>ath-miR398c MI0001019 Arabidopsis thaliana miR398c stem-loop				
TGTGTTCTCAGGTCACCCCTG	111885	12	43	245
>+1 ath-miR398c				
ATGTGTTCTCAGGTCACCCCTG	152	8	3	1

>ath-miR399b MI0001021 Arabidopsis thaliana miR399b stem-loop				
TGCCAAAGGAGAGTTGCCCTG	1154	0	16	1
>+1 ath-miR399b				
CTGCCAAAGGAGAGTTGCCCTG	34	0	0	0

>ath-miR399c MI0001022 Arabidopsis thaliana miR399c stem-loop				
TGCCAAAGGAGAGTTGCCCTG	1154	0	16	1
>+1 ath-miR399c				
TTGCCAAAGGAGAGTTGCCCTG	77	0	0	15

>ath-miR447a MI0002407 Arabidopsis thaliana miR447a stem-loop				
TTGGGGACGAGATGTTTTGTTG	179	0	3	4
>+1 ath-miR447a				
TTTGGGGACGAGATGTTTTGTTG	65	0	1	1

>ath-miR447b MI0002408 Arabidopsis thaliana miR447b stem-loop				
TTGGGGACGAGATGTTTTGTTG	179	0	3	4
>+1 ath-miR447b				
TTTGGGGACGAGATGTTTTGTTG	65	0	1	1

>ath-miR775 MI0005105 Arabidopsis thaliana miR775 stem-loop				
TTCGATGTCTAGCAGTGCCA	1788	4	22	44
>+1 ath-miR775				
TTTCGATGTCTAGCAGTGCCA	38	0	7	81
>+2 ath-miR775				
TTTTCGATGTCTAGCAGTGCCA	409	0	86	1092

>ath-miR777 MI0005107 Arabidopsis thaliana miR777 stem-loop				
TACGCATTGAGTTTCGTTGCTT	2	2	0	0
>+1 ath-miR777				

ATACGCATTGAGTTTCGTTGCTT	0	10	0	0
>ath-miR780.2 MI0005110 Arabidopsis thaliana miR780 stem-loop				
TTCTTCGTGAATATCTGGCAT	0	0	2	151
>+1 ath-miR780.2				
TTTCTTCGTGAATATCTGGCAT	2	0	0	37
>+3 ath-miR780.2				
GGTTTCTTCGTGAATATCTGGCAT	0	0	15	0
>ath-miR823 MI0005380 Arabidopsis thaliana miR823 stem-loop				
TGGGTGGTGATCATATAAGAT	1544	0	10	0
>+1 ath-miR823				
GTGGGTGGTGATCATATAAGAT	26	1	2	0
>ath-miR844 MI0005400 Arabidopsis thaliana miR844 stem-loop				
TGGTAAGATTGCTTATAAGCT	1	0	1	0
>+1 ath-miR844				
ATGGTAAGATTGCTTATAAGCT	4	4	0	0
>+2 ath-miR844				
AATGGTAAGATTGCTTATAAGCT	0	97	1	1
>ath-miR846 MI0005402 Arabidopsis thaliana miR846 stem-loop				
TTGAATTGAAGTGCTTGAATT	176	0	5	14
>+1 ath-miR846				
TTTGAATTGAAGTGCTTGAATT	13	0	0	0
>ath-miR863-3p MI0005440 Arabidopsis thaliana miR863 stem-loop				
TTGAGAGCAACAAGACATAAT	103	0	1	0
>+1 ath-miR863-3p				
ATTGAGAGCAACAAGACATAAT	1	0	0	0
>+2 ath-miR863-3p				
GATTGAGAGCAACAAGACATAAT	0	0	28	0
Total Sequences	1945650	15734	16551	7734
